# Medicinal Chemistry Progression of Sapanisertib, the Anticancer and Dual *Plasmodium* Phosphatidylinositol 4‑Kinase Beta and cGMP-Dependent Protein Kinase Inhibitor, for Malaria

**DOI:** 10.1021/acs.jmedchem.4c02799

**Published:** 2025-05-16

**Authors:** Samuel Gachuhi, Stephanie Kamunya, Stephen Fienberg, Lynn Wambua, Nicolaas Salomane, Godfrey Mayoka, Dale Taylor, Dina Coertzen, Mariette van der Watt, Janette Reader, Lyn-Marié Birkholtz, Sergio Wittlin, Liezl Krugmann, Lauren B. Coulson, Kelly Chibale

**Affiliations:** † Department of Chemistry, 37716 University of Cape Town, Rondebosch ,7701 Cape Town, South Africa; ‡ Holistic Drug Discovery and Development (H3D) Centre, 37716 University of Cape Town, Rondebosch, 7701 Cape Town, South Africa; § Institute of Infectious Disease and Molecular Medicine, 37716 University of Cape Town, Observatory, 7925 Cape Town, South Africa; ∥ Department of Biochemistry, Genetics and Microbiology, Institute for Sustainable Malaria Control, 56410 University of Pretoria, Hatfield, 0028 Pretoria, South Africa; ⊥ Department of Biochemistry, Stellenbosch University, Matieland, Stellenbosch 7602, South Africa; # 30247 Swiss Tropical and Public Health Institute, Kreuzstrasse 2, 4123 Allschwil, Switzerland; ∇ University of Basel, 4001 Basel, Switzerland; ○ South African Medical Research Council Drug Discovery and Development Research Unit, 37716 University of Cape Town, Rondebosch, 7701 Cape Town, South Africa

## Abstract

We recently demonstrated that the anticancer human mTOR inhibitor sapanisertib displays antimalarial activity in a malaria mouse model of infection and inhibits multiple *Plasmodium* kinases, including the high-value targets phosphatidylinositol 4-kinase type III beta (PI4Kβ) and cGMP-dependent protein kinase (PKG). Herein, we explore structure–activity relationships for sapanisertib analogues with benzyl and pyridyl substituents at the 7-position of the pyrazolopyrimidine core. New analogues with improved safety profiles were identified, including analogues with dual *Plasmodium* PI4Kβ and PKG inhibitory activity (exemplified by **19**), as well as potent *Plasmodium* PI4Kβ inhibitors with minimal inhibitory activity against PKG (exemplified by **20**). Compound **19** displayed potent antiplasmodium activity, high microsomal metabolic stability, and a good safety profile (hERG IC_50_ > 30; cytotoxicity selectivity index = 99). In vivo proof-of-concept, where a 4 × 50 mg kg^–1^ oral dose of **19** resulted in an 80% reduction in parasitemia in P. berghei-infected mice, further demonstrated the lead potential of this series.

## Graphical abstract

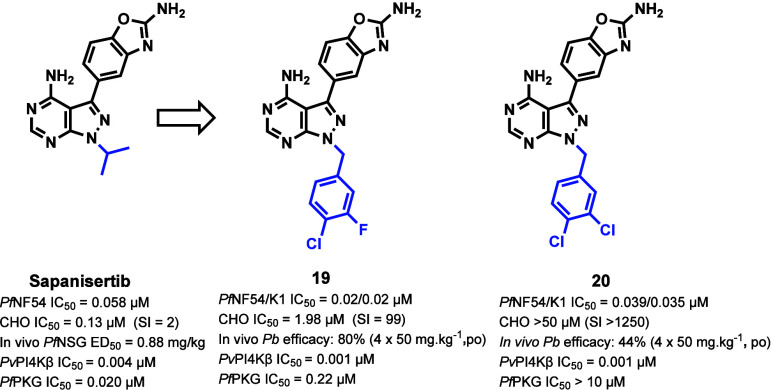

## Introduction

Human malaria is a deadly parasitic infectious disease caused by protozoan species in the genus *Plasmodium. Plasmodium* species that cause human disease include P. vivax, P. malariae, P. ovale, P. falciparum, and P. knowlesi. Of these P. falciparum causes the deadliest form of the disease. The World Health Organization (WHO) estimates that 263 million incidences occurred globally in 2023 with P. falciparum responsible for the majority of the 597,000 global malaria fatalities in the same year.^1^ Worryingly, this is an increase of over 10 million cases relative to the previous year. Although significant progress has been made to reduce malaria mortality and morbidity over the last few decades, the emergence and systematic spread of parasite resistance to previously and currently useful drugs for treatment including quinine, chloroquine, and artemisinin, the core component in the current frontline artemisinin-combination therapies (ACTs), continues to threaten advances made thus far.

Developing compounds with novel mechanisms of action relative to clinically used antimalarials remains crucial in the fight against malaria. Considering the success of kinase inhibitors for cancer and other diseases, with 80 kinase-targeting drugs now approved by the FDA for clinical use since the first approval in 2001,^2^ there is an opportunity to repurpose or reposition human kinase inhibitors for infectious diseases. Such approaches have resulted in kinase-targeted research for parasitic diseases including schistosomiasis, cryptosporidiosis, trypanosomiasis and malaria.^3–6^ Recently, we reported the multistage in vitro antiplasmodium activity and in vivo antimalarial efficacy of the human “mammalian target of rapamycin” (mTOR) inhibitor sapanisertib (also known as MLN0128, INK128, and TAK-228) currently under Phase II clinical evaluation for treatment of cancer.^7^ We demonstrated that *Pf*PI4Kβ was the primary efficacious target in asexual blood stage parasites and that in addition to *Pf*PI4Kβ, sapanisertib inhibits other *Plasmodium* kinases including cGMP-dependent protein kinase (PKG), another vulnerable *Plasmodium* kinase target essential to multiple stages of the parasite lifecycle.^7^

The antimalarial potential of targeting *Plasmodium* PI4Kβ has been demonstrated by the clinical candidate **MMV390048** (Figure 1), which displayed multistage activity in vitro and in vivo and reached Phase II clinical trials for the treatment of malaria.^8,9^ Despite good efficacy in the clinical studies, the development of **MMV390048** was stopped due to toxicity signals in rodents posing a potential teratogenicity risk.^10^ New *Plasmodium* PI4Kβ inhibitor chemotypes with distinct off-target profiles are now being explored as alternatives to **MMV390048**.

**Figure 1:**
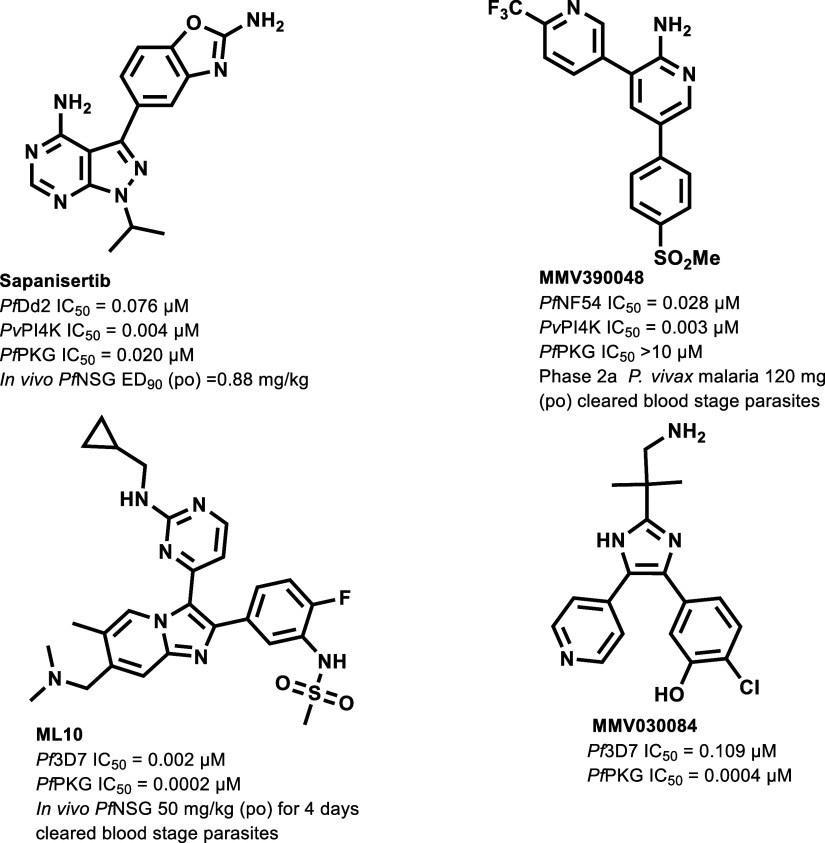
Sapanisertib and examples of previously explored *Plasmodium* PI4Kβ and PKG inhibitors.^7–12^ NSG, *NOD-scid IL-2R*γ*null* mouse model of P. falciparum infection*; po,* per oral.

*Pf*PKG also shows promise as a drug target with the potential to yield compounds with multistage antimalarial activity as demonstrated by the potent *Pf*PKG inhibitors **ML10** and **MMV030084** (Figure 1). In vivo proof-of-concept was demonstrated for **ML10** and PKG inhibitors have shown a low propensity for resistance,^11,12^ but the potential drawback of specific PKG inhibitors is their narrow temporal window of inhibitory activity (< 3 h) against the asexual blood stage parasite.^13^ Although PKG plays an essential role in the asexual blood stage particularly during schizont rupture and merozoite egression, its catalytic activity is not needed during the first 48-h period of intraerythrocytic parasite development.^14^ Consequently, such inhibitors show slow killing rates in the in vitro Parasite Reduction Ratio (PRR) assay.

*Plasmodium* PI4Kβ and PKG continue to attract attention as promising antimalarial drug targets with the potential to deliver antimalarials with prophylactic and transmission blocking activity. Dual inhibition of these essential kinases has the potential to provide a stronger barrier to the onset of resistance and improve efficacy. Targeting multiple *Plasmodium* kinases simultaneously may have advantages over single-target therapeutics due to possible synergy and absence of pharmacokinetic mismatch during advanced clinical drug combination studies.^6^ As for any kinase-focused drug discovery program, toxicity risks due to off-target human kinase inhibition will need to be carefully monitored during the optimization of multi-kinase inhibitors.

Molecular features responsible for the high affinity of sapanisertib for the *Plasmodium* PI4Kβ and PKG ATP binding sites were identified computationally using a PI4Kβ homology model and published PKG crystal structures.^7^ Using the same modelling techniques, we have hypothesized that replacement of the isopropyl moiety in sapanisertib with benzyl and pyridyl substituents may deliver analogues with dual affinity for the aforementioned *Plasmodium* targets, and an avenue for exploring selectivity. Better selectivity for *Plasmodium* kinases may improve the safety profile of sapanisertib, a key issue that has been flagged in vivo.^7^ While initial cytotoxicity profiling against the HepG2 cell line indicated a moderate selectivity window as measured by the selectivity index (SI = HepG2 IC_50_/*Pf*Dd2 IC_50_ = 79),^7^ subsequent cytotoxicity profiling against the Chinese Hamster Ovarian (CHO) cell line indicated a cytotoxicity risk. Herein, we report the antiplasmodium activity, cytotoxicity, pharmacokinetics, and in vivo efficacy of a series of sapanisertib analogues that potently inhibit *Plasmodium* PI4Kβ or dually inhibit PI4Kβ and PKG in vitro.

## Results and Discussion

### Design and Synthesis

Recently, we reported the molecular docking studies of sapanisertib into the ATP binding sites of *Plasmodium* PKG and PI4Kβ using the respective crystal structure and homology model.^7^ Consistent with the in vitro kinase data (*Pv*PI4Kβ IC_50_ = 0.004 μM; *Pf*PKG IC_50_ = 0.020 μM;), the in silico docking studies predicted the existence of strong interactions between the adenine-like pyrazolopyrimidine core and the hinge region, and the benzoxazole moiety in the affinity/back pocket of the ATP binding sites. However, no interactions were observed between the isopropyl group and the ribose pocket.^7^ Here, we investigate the effects of replacing the isopropyl group with an *N*-alkylated substituted benzyl ring on *Plasmodium* PI4Kβ and PKG inhibition and other parameters including antiplasmodium activity and cytotoxicity. A range of Craig plot functionalities^15^ at the *ortho*, *meta* and *para* positions of the benzyl ring were investigated.

Accordingly, analogues were synthesized based on Scheme 1. The crucial precursor **2** was synthesized from commercially sourced 5-amino-1*H*-pyrazole-4-carbonitrile in high yield (93%) via a Leuckart condensation reaction in formamide based on a previously described scheme.^16^ An aromatic nucleophilic substitution reaction of this intermediate with *N*-iodosuccinimide ensured the delivery of the iodinated intermediate (**3**) in a relatively high yield (89%). The *N*-alkylated diversity required on the pyrazole nitrogen of the iodinated precursor **3** to intermediates **6a**–**38a** was achieved via a bimolecular nucleophilic substitution (S_N_2) with commercial alkyl halide in DMF with K_2_CO_3_ as a base. These intermediates were obtained in moderate to high yields (37-71%). Several alkyl halides used in this reaction were obtained from their corresponding alcohols or carboxylic acids. Finally, Suzuki-Miyaura cross-coupling reactions on the series of the generated *N*-alkylated intermediates with a benzoxazole-derived boronic ester (**5**), delivered the target compounds in low to moderate yields (14–57%).

**Scheme 1:**
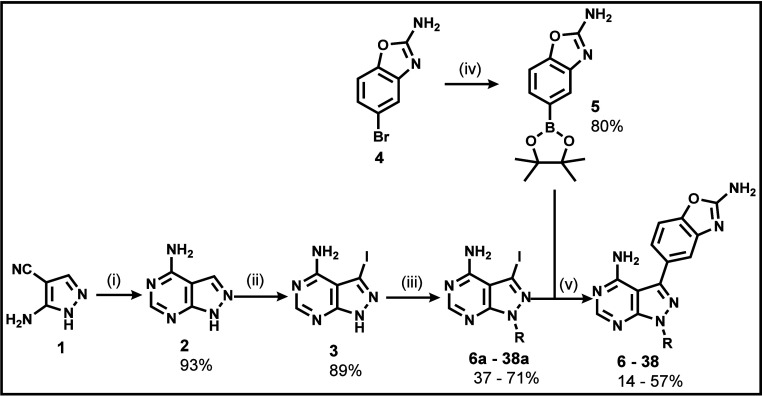
General Synthetic Approach for Target Analogues **6**–**38** ^a^

a Reagents and reaction conditions: (i) HCONH_2_, DMF, 180 °C, 15 h, 93%; (ii) NIS, DMF, 80 °C, 15 h, 89%; (iii) RBr or RCl, K_2_CO_3_, DMF, 30 °C for RBr and 70 °C for RCl, 2 h, 37–71%; (iv) bis­(pinacolato)­diborane, Pd­(dppf)­Cl_2_, KOAc, dioxane, 100 °C, 15 h, 80%; (v) Pd­(PPh_3_)_4_, Na_2_CO_3_, dioxane/H_2_O (3:1), 100 °C, 15 h, 14–57%.

### In Vitro Asexual Blood Stage Antiplasmodium Activity and Intrinsic Solubility Profiling

Compounds were tested against the chloroquine-(CQ) sensitive NF54 strain of P. falciparum to investigate the effect of the benzyl substituents on antiplasmodium activity (Table 1). *Para* and *meta*-substituted analogues containing small electron-withdrawing groups (EWGs), particularly F and Cl groups, exhibited higher potency than larger groups such as trifluoromethyl (CF_3_) or Me substituents. The *para* substituents were also better tolerated than the corresponding *meta*-substituents. For example, the *para*-Cl analogue **7** displayed more potent antiplasmodium activity (*Pf*NF54 IC_50_ = 0.029 μM) than the *meta*-Cl-containing compound **13** (*Pf*NF54 IC_50_ = 0.129 μM). Similarly, *p-*F substituted analogue **8** (*Pf*NF54 IC_50_ = 0.141 μM) displayed a two-fold lower IC_50_ value relative to its *m*-substituted counterpart **11** (IC_50_ = 0.297 μM). In contrast, all the *ortho*-substituted analogues showed poor activity (IC_50_ > 1 μM; Table 1), while the non-substituted benzyl **6** displayed modest activity (IC_50_ = 0.719 μM), highlighting the importance of a lipophilic group at this position for antiplasmodium activity.

**Table 1:**
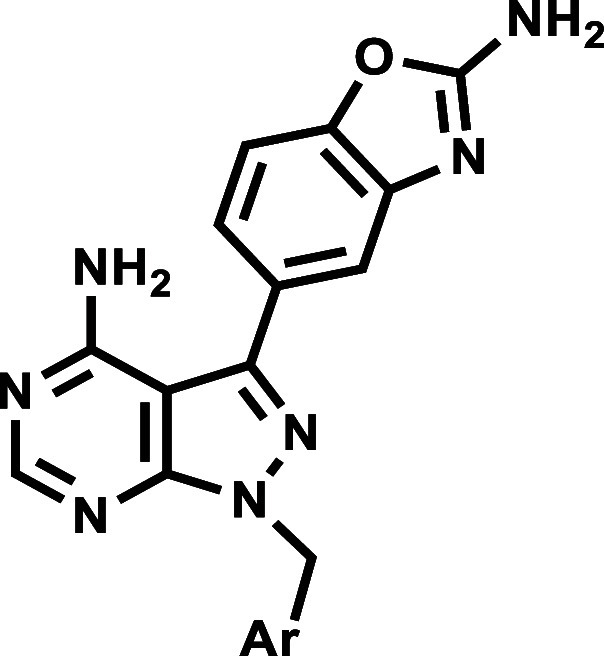

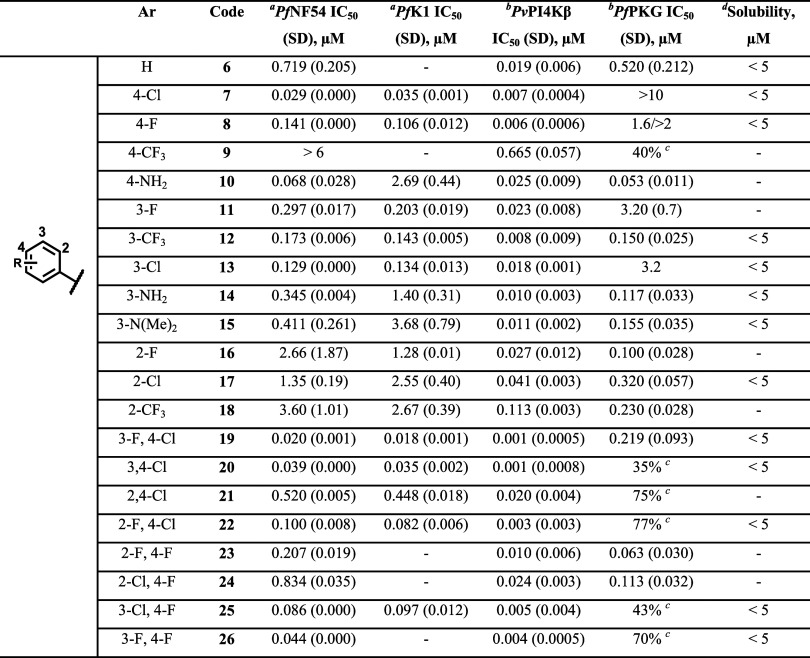
In Vitro Asexual Blood Stage Antiplasmodium Activity, In Vitro *Pv*PI4Kβ and *Pf*PKG Inhibition, and Solubility of Benzyl Derivatives

a Mean from *n* ≥ 2 independent experiments (individual IC_50_ values differed by ≤2-fold); All the compounds were tested for in vitro asexual blood stage antiplasmodium activity using 72 h parasite lactate dehydrogenase (pLDH) assay with exception of compounds **7**, **8**, **11**–**13**, **19**–**22** and **26** for which a modified 72 h [^3^H]-hypoxanthine incorporation assay was employed. Artesunate [IC_50_ = 4.0 nM (NF54)], chloroquine [IC_50_ = 16 nM (NF54)] were used as positive controls.

b Inhibition of recombinant *Pv*PI4Kβ and *Pf*PKG in the presence of 10 μM ATP was measured using ADP-Glo kinase assays. Mean IC_50_ values were calculated based on *N* ≥ 2 independent experiments, each with technical duplicates.

c Mean percentage kinase inhibition at 10μM inhibitor concentration was determined in the presence of 10 μM ATP based on the mean of *N* = 2 independent experiments, each carried out in triplicate. ML10 (LifeArc) [IC_50_ = 0.6 nM *Pf*PKG] and MMV390048 [IC_50_ = 3 nM *Pv*PI4K] were used as positive controls.

d All values were determined using an HPLC-based miniaturized shake flask method at pH 6.5. “-” = data not generated.

A handful of compounds were also tested against the multi-drug resistant *Pf*K1 strain (Table 1). No significant differences in antiplasmodium activity were observed across the *Pf*NF54 and K1 strains, with the exception of the basic aniline derivatives (**10;** *Pf*K1 IC_50_ **=** 2.69 μM, **14**; *Pf*K1 IC_50_ **=** 1.40 μM, and **15**; *Pf*K1 IC_50_ **=** 3.68 μM) which displayed reduced potency against the multi-drug resistant strain. However, despite these benzyl and pyridyl substitutions being tolerated for antiplasmodium activity, they were detrimental to solubility as all the tested compounds showed low intrinsic solubility (< 5 μM) at pH 6.5 (Table 1).

Encouraged by the potent antiplasmodium activity observed for analogues **7** and **8**, we further expanded the SAR by exploring di-substituted benzyls with F and Cl groups in the *para* position and various electron-withdrawing and -donating groups in the *ortho* or *meta* position (**19**–**26**). The antiplasmodium potency was retained for analogues containing a disubstituted benzyl moiety with either Cl or F group in the *para* position. Notably, di-substituted *para* F/Cl analogues showed equipotency as observed in **20** (*Pf*NF54 IC_50_ = 0.039 μM) and **19** (IC_50_ = 0.020 μM) relative to the initially identified lead mono-chlorinated compound **7** (IC_50_ = 0.029 μM), while a modest three-fold decrease in potency was observed for the 2-F, 4-Cl di-substituted analogue **22** (IC_50_ = 0.100 μM). In contrast, 2,4-Cl di-substitution was detrimental to potency as a > 10-fold decrease in potency was observed for analogue **21** (IC_50_ = 0.520 μM) relative to analogue **7**. Similarly, maintaining F in the *para* position ensured potency for 3,4-disubstituted compounds, with improved or similar activity compared to that of the *para*-monoflourinated lead compound **8** (IC_50_ = 0.141 μM). Of note is the more potent 3,4-difluoro analogue **26** (IC_50_ = 0.044 μM) and the 3-Cl, 4-F-substituted congener **25** (IC_50_ = 0.086 μM). Compounds **25** and **19**–**22** showed comparable asexual blood stage activity against the multidrug resistant K1 strain, indicating that the risk of cross-resistance with currently used clinical drugs is low. Swapping the positions of the F and Cl substituents had a modest impact on antiplasmodium activity based on the four-fold difference in activity observed for analogue **25** (IC_50_ = 0.086 μM) and **19** (IC_50_ = 0.020 μM). However, substitution at the *ortho* position while maintaining the F atom at the *para* position was detrimental to potency, as demonstrated by **23** (IC_50_ = 0.207 μM), and **24** (IC_50_ = 0.834 μM). No significant improvement in aqueous solubility was observed for this set of compounds.

To improve solubility, we generated a set of analogues with the pyridyl functionality while retaining the F or Cl group at the *para* or *meta* position (**27–38**, Table 2). Modifications in this SAR set were also well tolerated with analogues exhibiting sub-micromolar antiplasmodium activity (IC_50_ = 0.081–0.950 μM). However, a modest (two- to four-fold) decline in the antiplasmodium potency was observed relative to the benzyl counterparts. The position of the pyridyl nitrogen had minimal impact on antiplasmodium potency, as observed in the *meta*-substituted Cl analogues **32** (*Pf*NF54 IC_50_ = 0.104 μM) and **35** (IC_50_ = 0.151 μM). This strategy led to some improvement in aqueous solubility at pH 6.5, particularly regarding the non-substituted pyridyls such as **31** (30 μM), and **33** (20 μM; Table 2), but these compounds showed reduced antiplasmodium activity with respective *Pf*NF54 IC_50_ values of 0.95, and 0.79 μM. Attempts to regain activity by substitution on the pyridyl ring negated the improved solubility as all substituted pyridyls tested showed low solubility (≤10 μM). This was attributed to the addition of the lipophilic groups. Additionally, several pyridyls such as **32** (*Pf*NF54 IC_50_ **=** 0.10 μM/ *Pf*K1 IC_50_ **=** 2.51 μM, Resistance Index (RI) = 24), showed a heightened risk for cross-resistance when tested on the multi-drug resistant *Pf*K1 strain.

**Table 2:**
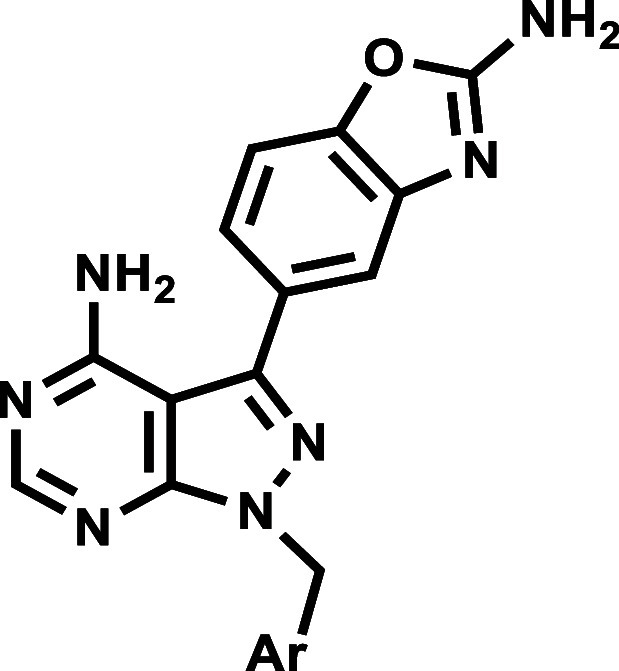

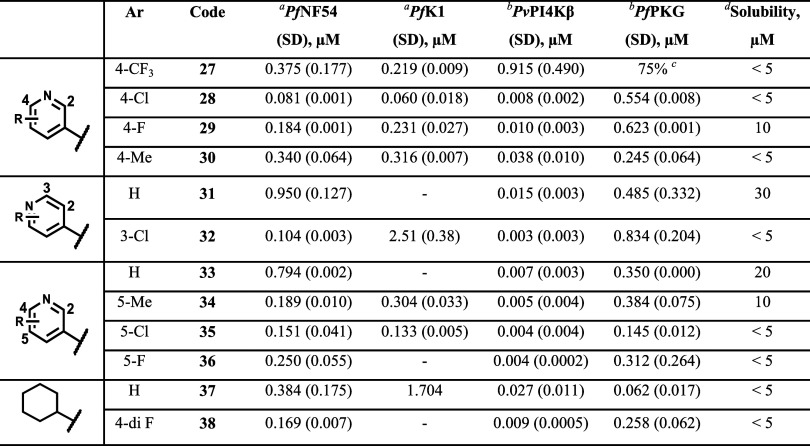
In Vitro Asexual Blood Stage Antiplasmodium Activity, In vitro *Pv*PI4Kβ and *Pf*PKG Inhibition, and Solubility of Pyridyl and Cyclohexyl Derivatives

a Mean from *N* ≥ 2 independent experiments (individual IC_50_ values differed by ≤2-fold); All the compounds were tested for in vitro asexual blood stage antiplasmodium activity using the 72 h parasite lactate dehydrogenase (pLDH) assay with exception of compounds **27**, and 28 in which a modified 72 h [^3^H]-hypoxanthine incorporation assay was employed. Artesunate [IC_50_ = 4.0 nM (NF54)] and chloroquine [IC_50_ = 16 nM (NF54)] were used as positive controls.

b Inhibition of recombinant *Pv*PI4Kβ and *Pf*PKG in the presence of 10 μM ATP was measured using ADP-Glo kinase assays. Mean IC_50_ values were calculated based on *N* ≥ 2 independent experiments, each with technical duplicates.

c Mean percentage kinase inhibition at 10μM inhibitor concentration was determined in the presence of 10 μM ATP based on the mean of *N* = 2 independent experiments, each carried out in triplicate. ML10 (LifeArc) [IC_50_ = 0.6 nM *Pf*PKG], and MMV390048 [IC_50_ = 3 nM *Pv*PI4K] were used as positive controls.

d All values were determined using an HPLC-based miniaturized shake flask method at pH 6.5. “-” = data not generated.

Several compounds with cyclohexyl substituents (**37** and **38**) were also explored. Introduction of the aliphatic cyclohexyl ring resulted in compounds that retained antiplasmodium activity (**38**; *Pf*NF54 IC_50_ = 0.169 μM) with equally poor solubility, whereas further modification to carboxamides and sulfonamides led to significant loss in antiplasmodium activity (*Pf*NF54 > 1 μM), although the intrinsic solubility was greatly improved (Table S1 in the Supporting Information).

### *Plasmodium* PI4Kβ Inhibition and In Silico Docking Studies

The in vitro PI4Kβ inhibition was assessed using purified recombinant *Pv*PI4Kβ protein in an ADP detection assay as previously described.^7,17^ The catalytic domains of *Pf*PI4Kβ and *Pv*PI4Kβ are well conserved, sharing 97% sequence similarity and residues predicted to form ATP binding sites are identical. Consequently, *Pv*PI4Kβ serves as an adequate surrogate for *Pf*PI4Kβ which is more challenging to express recombinantly.

*Pv*PI4Kβ IC_50_ values are shown in Tables 1 and 2. Compound **19** and **20** showed the most potent *Pv*PI4Kβ activity (IC_50_ ≤ 0.001 μM), corresponding to the lower detection limit of the assay. Potent *Pv*PI4Kβ inhibition translated into high antiplasmodium potency (*Pf*NF54 IC_50_ = 0.020 and 0.039 μM, respectively) as previously highlighted. Compound **7** which was equally potent against *Pf*NF54 and *Pf*K1 strains (PfNF54/K1 IC_50_ = 0.029/0.035 μM), also exhibited potent in vitro *Pv*PI4Kβ inhibition with an IC_50_ value of 0.007 μM. Other analogues highly potent against *Pv*PI4Kβ with good antiplasmodium activities were **22** and **25** (Table 1). From the results, it is evident that the presence of a Cl or F group in the *para* or *meta* positions favored antiplasmodium potency and *Pv*PI4Kβ inhibitory activity, with Cl-substitution in the *para* position resulting in superior antiplasmodium activity. This is exemplified by analogue **7** (*Pf*NF54 IC_50_ = 0.029 μM; *Pv*PI4Kβ IC_50_ = 0.007 μM) and **8** (*Pf*NF54 IC_50_ = 0.141 μM; *Pv*PI4Kβ IC_50_ = 0.006 μM) as well as the disubstituted congeners. In contrast, *ortho* substitution led to a decrease in *Pv*PI4Kβ activity and whole cell potency as exemplified by the mono-substituted F analogue **16** (*Pf*NF54 IC_50_ =2.66 μM; *Pv*PI4Kβ IC_50_ = 0.027 μM) and Cl counterpart **17** (*Pf*NF54 IC_50_ = 1.35μM; *Pv*PI4Kβ IC_50_ = 0.041 μM). Incorporation of the larger and more lipophilic CF_3_ group at the *para* position resulted in the least potent benzyl analogue (**9**, *Pf*NF54 IC_50_ > 6 μM; *Pv*PI4Kβ IC_50_ = 0.665 μM).

The sapanisertib series of compounds all docked into the *Pf*PI4Kβ homology model with the expected pose, as illustrated in Figure 2 for **9** and **19**, where the adenine-like core hydrogen bonds to the hinge backbone carbonyl of V1357 and the amino benzoxazole interacts with the catalytic K1308 and D1430 residues. F827 from the P-loop also makes a π-stacking interaction with the adenine core and the substituted benzyl group.

**Figure 2:**
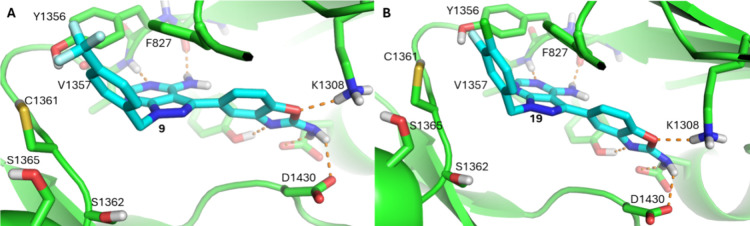
Docking poses of **9** (A) and **19** (B) in the *Pf*PI4Kβ ATP binding site. (A) Compound **9** docked into the *Pf*PI4Kβ homology model with the expected hinge binding motif H-bonding to the purine core, the amino-benzoxazole H-bonding to the catalytic residues of D1430 and K1308 while the *para*-CF_3_ phenyl group interacts with the with the ribose pocket projecting out towards the solvent front. (B) Compound **19** has an almost identical pose to **9** with its 3,4 Cl, F phenyl group occupying an identical position in the ribose pocket near the solvent front.

In addition, all tested pyridyl compounds with *para-* and *meta*-F and -Cl substituents showed good *Pv*PI4Kβ potency (IC_50_ < 0.010 μM) regardless of the position on the pyridine ring (Table 2). Notable compounds included the *para*-Cl pyridyl analogue **28** (*Pv*PI4Kβ IC_50_ = 0.008 μM), and the *meta*-Cl congener **35** (*Pv*PI4Kβ IC_50_ = 0.004 μM). The docking model predicts that related pyridyl and benzyl compounds dock with a similar pose, consistent with the potent *Pv*PI4Kβ inhibition and corresponding antiplasmodium activity observed for compounds in this series.

### *Plasmodium* PKG Inhibition and In Silico Docking Studies

The potential of this class of compounds to act as dual *Plasmodium* PI4Kβ and PKG inhibitors was evaluated by testing compounds against a recombinant *Pf*PKG protein using an ADP detection assay as previously described.^11,12^ Compounds in this series generally showed weak to moderate *Pf*PKG inhibition (IC_50_ values between 0.053 and >10 μM) relative to the more potent *Pv*PI4Kβ inhibition (IC_50_ < 0.02 μM), as highlighted in the previous subsection (Tables 1 and 2). The 4-amino benzyl analogue **10** and the cyclohexyl-substituted analogue **37** displayed the highest potency against *Pf*PKG with respective IC_50_ values of 0.053 and 0.062 μM. Furthermore, all analogues with basic side chains displayed moderate *Pf*PKG potencies as exemplified by **14** (*Pf*PKG IC_50_ = 0.117 μM) and **15** (*Pf*PKG IC_50_ = 0.155 μM). Similarly, the *ortho*-F benzyl **16** also displayed moderate potency with an IC_50_ value of 0.100 μM.

Compounds with aliphatic groups exhibited good enzymatic potency as exemplified by the cyclohexyl-substituted analogue **37** (*Pf*PKG IC_50_ = 0.062 μM). Difluorination of the cyclohexyl substituent in **38** resulted in a four-fold decline in potency (*Pf*PKG IC_50_ = 0.258 μM). However, all pyridyls tested displayed moderate in vitro potency (*Pf*PKG IC_50_ < 1 μM) against the recombinant protein. Pyridyl compound **35** showed good potency against the enzyme with a *Pf*PKG IC_50_ of 0.145 μM. Other notable pyridyls included the methyl substituted analogues **30** (*Pf*PKG IC_50_ = 0.245 μM), and **34** (*Pf*PKG IC_50_ = 0.384 μM).

By contrast, despite their potent inhibition against the *Pv*PI4Kβ enzyme, a significant number of *para-* and *meta*-substituted benzyls displayed a complete loss of *Pf*PKG inhibitory activity. For example, the *para*-Cl analogue **7** (*Pv*PI4Kβ IC_50_ = 0.007 μM) and *para*-F **8** (*Pv*PI4Kβ IC_50_ = 0.006 μM) previously highlighted, displayed weak *Pf*PKG inhibition (IC_50_ ≥ 1.6 μM). Similarly, *para*-trifluoromethyl (CF_3_) substituted analogue **9** showed weak *Pf*PKG inhibition (40% inhibition at 10 μM). By comparison, the unsubstituted benzyl **6** exhibited a *Pf*PKG IC_50_ of 0.52 μM, suggesting that substitution with an electron withdrawing group was generally unfavorable for *Pf*PKG potency. Similar observations were made with substitution on the *meta* position, as exemplified by **13** (IC_50_ = 3.2 μM).

The predicted binding interactions for this series in the *Plasmodium* PKG ATP binding site are exemplified in Figure 3. The *Pv*PKG crystal structure in complex with 1-*tert*-butyl-3-(3-chlorophenoxy)-1*H*-pyrazolo­[3,4-*d*]­pyrimidin-4-amine inhibitor was selected for docking studies due to the structural similarity of the bound inhibitor with this series. Compound **14** (*Pf*PKG IC_50_ = 0.117 μM) docked into a PDB structure of *Pv*PKG (PDB ID: 5F0A, Figure 3A) with the expected pose with the adenine core forming a H-bond acceptor/donor pair interaction with the hinge (L613 and V614). The benzoxazole of **14** forms an H-bond with the backbone amide of D675 and the amide on this benzoxazole group may form an H-bond with E582 in the back pocket. The benzyl substituent of **14** interacts with lipophilic residues in the ribose pocket like L644 and the meta-NH_2_ on this benzyl group is predicted to form an H-bond with the acidic side chain of D675. This H-bond likely plays a sizeable role in the higher *Pf*PKG potency of **14.** Compound **37** (*Pf*PKG IC_50_ = 0.062 μM) is predicted to bind with the same hinge, catalytic region and back pocket interactions as **14** (Figure 3B). The different aliphatic cyclohexyl group interacts with the lipophilic region of the ribose pocket near L644, explaining its good potency.

**Figure 3:**
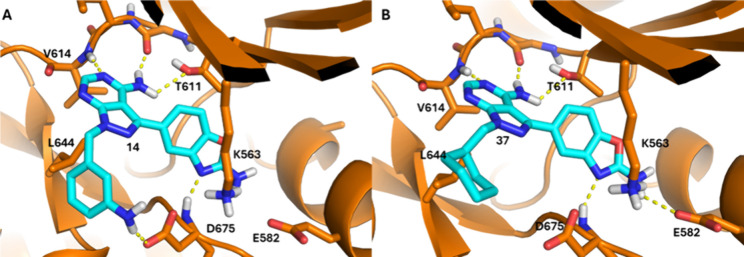
Docking poses of **14** and **37** in the *Pv*PKG ATP binding site (PDB ID 5F0A). (A) Compound **14** (cyan, *Pf*PKG IC_50_ = 0.117 μM) docked into the ATP binding site of *Pv*PKG (orange). (B) Compound **37** (cyan, *Pf*PKG IC_50_ = 0.062 μM) docked into the ATP binding site of *Pv*PKG (orange).

As discussed above, compounds in this series generally exhibited potent in vitro *Pv*PI4Kβ inhibition with weaker more variable *Pf*PKG inhibition. The benzyl analogues containing basic side chains (such as **10**, **14**, and **15**; Table 1) and pyridyl analogues (such as **30** and **35**) showed the most potent dual *Plasmodium* PI4Kβ and PKG inhibition (*Pv*PI4Kβ IC_50_ < 0.02 μM, and *Pf*PKG IC_50_ < 0.25 μM).

Despite the promising dual inhibition of sapanisertib and several other analogues from this series, the whole-cell antiplasmodium activity was found to be more dependent on PI4Kβ inhibition than PKG inhibition. Other targets may also contribute to the antiplasmodium activity of some analogues. A challenge for designed polypharmacology is effectively balancing potency against the targets to ensure the advantages of dual targeting are realized in the cellular context in vivo. For kinase targets, this will be affected by any differences in *K*_*m*_^ATP^ between the two targets resulting in different degrees of ATP competition at high cellular ATP concentrations, in addition to the relative vulnerability of the targets, which is more challenging to quantify.

Compound **10** is an example of a dual inhibitor in this series, showing the best balance of activity for the two targets (*Pv*PI4Kβ IC_50_ = 0.025 μM/*Pf*PKG IC_50_ = 0.053 μM). This compound also showed good antiplasmodium whole-cell activity in the *Pf*NF54 strain (IC_50_ = 0.068 μM). However, this compound showed reduced activity against the K1 strain (IC_50_ = 2.69 μM), indicating that common resistance mechanisms, pre-existing in the field, may reduce compound efficacy. The docking models predict that the 4-aniline group makes polar interactions with the ribose pocket of both PI4Kβ, and PKG, accounting for its dual inhibition (Figure 4). A better balance between *Plasmodium* PKG and PI4Kβ potency will likely be required to fully realize the benefits of dual targeting. Future work will need to focus on significantly improving PKG potency, particularly because the PKG *K*_*m*_^ATP^ (∼20 μM) is lower than for PI4Kβ (∼300 μM), so higher PKG inhibitor potency will be required to achieve 50% PKG inhibition at high ATP concentrations in the cellular environment than for PI4Kβ.

**Figure 4:**
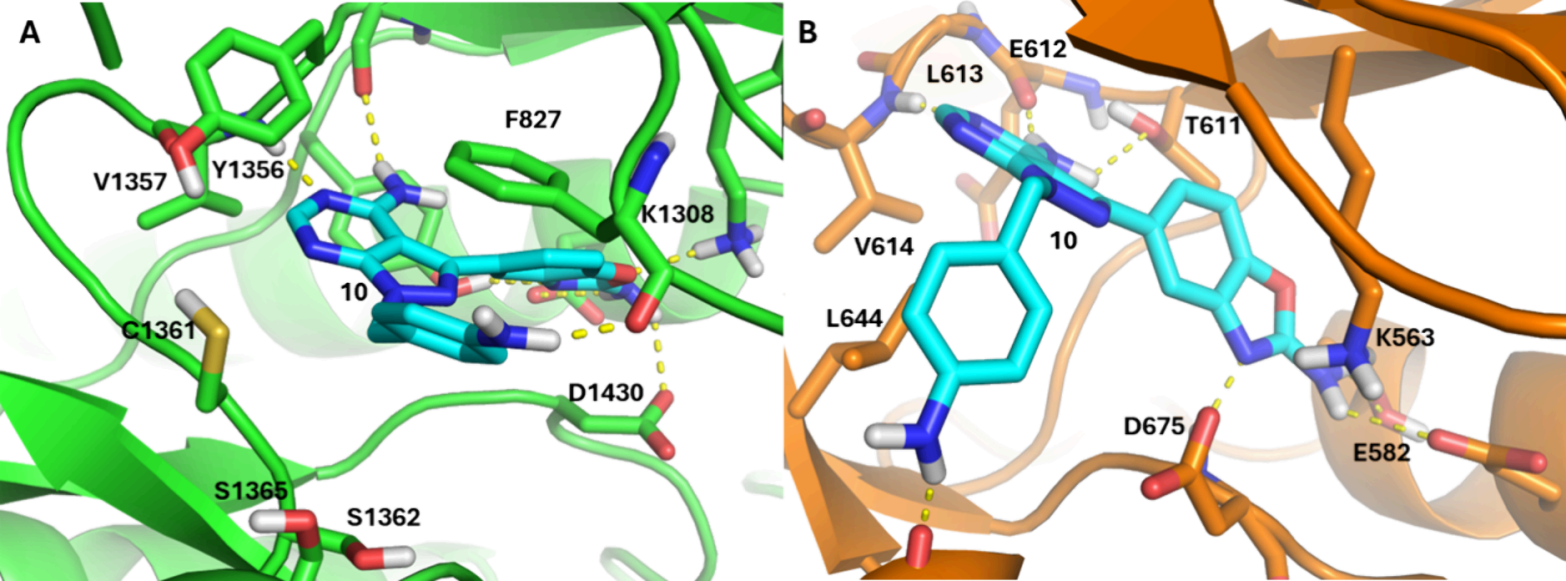
Compound **10** docked into *Pf*PI4Kβ and *Pv*PKG. (A) Compound **10** (cyan, *Pv*PI4Kβ IC_50_ = 0.025 μM) docked into the homology model of *Pf*PI4Kβ (green) with the conserved core pose for the adenine and benzoxazole groups and unique interactions between the 4-aniline and the ribose pocket. (B) Compound **10** (cyan, *Pf*PKG IC_50_ = 0.053 μM) docked into *Pv*PKG with the same core pose for the adenine and benzoxazole groups with the aniline group finding a new H-bond in the ribose pocket.

It is also worth noting that a potential risk emanating from dual inhibition of two distinct kinases, such as *Pf*PI4Kβ and PKG, is that the series could encroach on promiscuous kinase inhibitor chemical space, so the host off-target kinase liability also needs to be assessed.

### Off-Target Human PI4Kβ and mTOR Inhibition

A key challenge for the development of *Plasmodium* PI4Kβ inhibitors is obtaining selectivity over the human PI4Kβ orthologue and related phosphoinositide kinases. This is principally due to the high conservation of the ATP binding site in *Plasmodium* PI4Kβ and related human host phosphoinositide kinases.^18^ Sapanisertib displays a distinct human kinase inhibition profile relative to other reported PI4Kβ series, displaying minimal activity against human PI4Kβ but potent inhibition of human mTOR.^7^ Given that small structural changes can influence the selectivity profile, several analogues were tested for off-target activity against both human mTOR and PI4Kβ (Table 3).

**Table 3:**
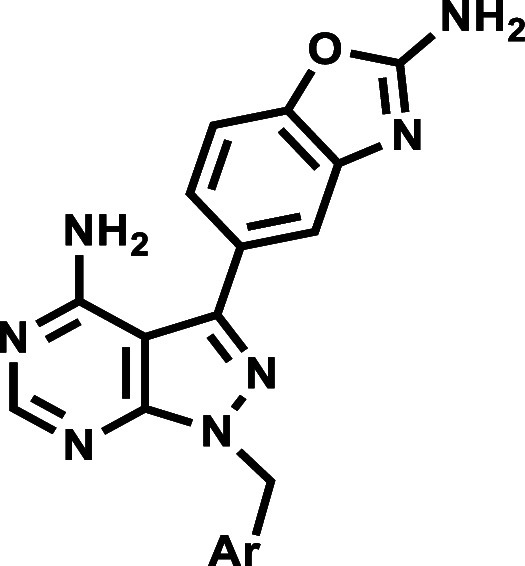

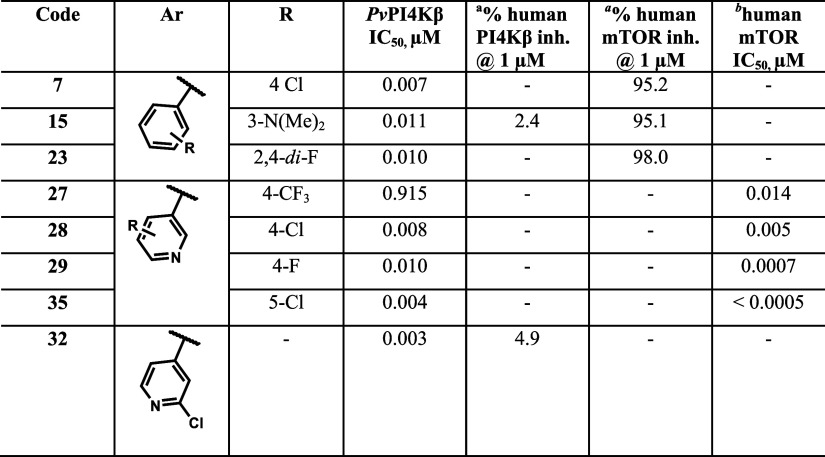
Comparison of In Vitro Inhibition of *Pv*PI4Kβ, *Hu*PI4Kβ, and mTOR

a Percentage inhibition relative to vehicle control, *N* = 1 biological assay with technical triplicates (*n* = 3).

b 10-point dose-response assays carried in the presence of 10 μM ATP. PI-103 (*Hu*mTOR IC_50_ = 84.3 nM), and PIK-93 (*Hu*PI4Kβ IC_50_ = 12.5 nM) were used as positive controls for single point and dose-response assays. “-” = data not generated.

All the benzyl and pyridyl compounds analyzed from this series showed little inhibitory activity (<5% inhibition at 1 μM; Table 3) against the human PI4Kβ but retained potent human mTOR inhibition (>95% at 1 μM or IC_50_ < 15 nM)_._ Similar observations were made for related carboxamides and sulfonamides (*Hu*PI4Kβ IC_50_ > 10 μM for all the tested compounds; *hu*mTOR IC_50_ 0.002–0.277 μM) (Table S2 in the Supporting Information) indicating that compounds retained selectivity for *Plasmodium* PI4Kβ relative to human PI4Kβ. Docking studies using the human PI4Kβ crystal structure (PDB code 6GL3) found no plausible pose for any compound when docked against HuPI4Kβ suggesting a low inhibition risk for this human off-target. Once other liabilities have been addressed, more advanced compounds will need to be tested against a larger panel of human kinases to gain further insight into potential kinase-related toxicity risks.

### Cytotoxicity Screening against the Chinese Hamster Ovarian Cell-Line

To further assess potential compound safety, cytotoxicity was tested using the colorimetric 3-(4,5-dimethylthiazol-2-yl)-2,5-diphenyltetrazolium bromide (MTT) assay in CHO cells.^19^ IC_50_ values and selectivity indices (SI; CHO IC_50_/*Pf*NF54 IC_50_) for representative compounds are shown in Table 4. A significant number of mono-substituted *para* and *meta* benzyl analogues exhibited SI > 100 as exemplified by 13 with CHO IC_50_ > 50 μM, translating to SI value >388 (Table 4). Exceptions to this included the *para*-Cl analogue **7** with an SI of 40. In contrast, di-substitution increased the potential for inducing cytotoxicity in CHO cells as all disubstituted benzyls showed poor SIs (<100), except for 3,4 di-chloro in **20** (SI > 1250) and 3-Cl, 4-F substitution in **25** (SI = 267). The 3,4-di-chlorinated analogue **20** showed the most favorable safety profile (SI > 1250) of all analogues tested.

**Table 4:**
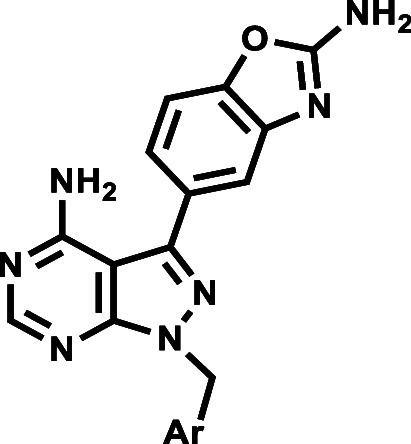

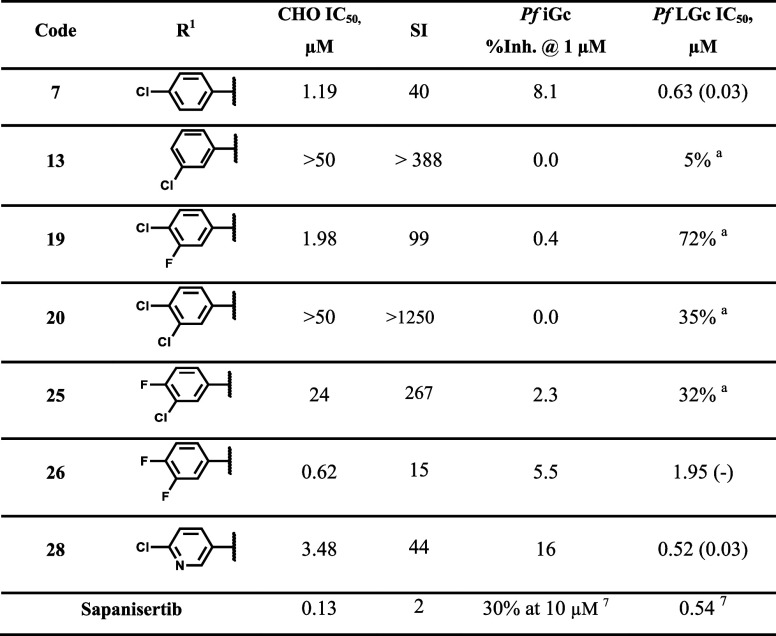
Cytotoxicity Data and In Vitro Activity against Immature and Late-Stage Gametocytes of P. falciparum NF54 for Selected Compounds^b^

a % inhibition of iGc at 1 μM drug concentration. “-” = data not generated.

b *Pf* iGc, Plasmodium falciparum immature gametocytes (> 90% stage I–III); *Pf* LGc, Plasmodium falciparum late-stage gametocytes (LGc, > 90% stage IV/V) *Pf* LGc IC_50_ data are the mean of n = 3 independent experiments, each carried out in triplicate. SEM, standard error of the mean.

Additionally, changing the positions of the substituents showed no significant improvement, except for the 3-F, 4-Cl modification in analogue **19** (SI = 99). A head-to-head comparison with its isomeric congener **25** (SI = 267) showed a dramatic shift in SI, suggesting that minor modifications can impact heavily on the cytotoxicity profile of compounds. The pyridyl analogues all showed cytotoxicity SI < 100, except compounds **27** (SI > 133), **32** (SI = 393), and **34** (SI = 210). Furthermore, there appeared to be no favored pyridyl position for enhanced selectivity, as no specific SAR could be deduced. Equally, replacement of the aromatic moiety with an aliphatic cyclohexyl was not beneficial, as evidenced by the low SI of **37** (SI = 15) and **38** (SI = 1.2; Table S3 in the Supporting Information).

### In Vitro Activity against *Pf* Immature and Late-Stage Gametocytes

The in vitro gametocytocidal activity of selected compounds showing potent asexual blood stage activity (*Pf*NF54 IC_50_ ≤ 0.5 μM) was tested using the luciferase-reporter *Pf*NF54 line to assess the transmission-blocking potential of this series. Compounds were screened for activity against in vitro immature gametocytes (iGc, >90% stage I–III) and late-stage gametocytes (LGc, >90% stage IV/V).^20^ Initially, each compound was investigated for % inhibition against the two stages at 5 and 1 μM. Compounds displayed specificity for late-stage gametocytes as was observed for sapanisertib,^7^ with 18 compounds exhibiting >70% inhibition at a concentration of 5 μM (Table S4 in the Supporting Information). None of the tested compounds showed dual activity (>50% inhibition) against the two sexual stages of development at 1 μM drug concentration. Compounds with reproducibly high single-point inhibition at 1 μM (>75%; Table 4), were tested in dose-response assays for IC_50_ determination (*n* = 1 experiment in technical duplicates).

Compounds **28** and **7** demonstrated high potency against late-stage gametocytes with respective IC_50_ values of 0.52 and 0.62 μM. These compounds also exhibited potent asexual blood stage activity and *Pv*PI4Kβ inhibition (**28;** *Pf*NF54 IC_50_ = 0.081 and *Pv*PI4Kβ IC_50_ = 0.008; **7**; *Pf*NF54 = 0.029 μM and *Pv*PI4Kβ = 0.007 μM).

While PI4Kβ is expressed across gametocyte development and PI4Kβ inhibitors have been shown to inhibit both immature and late-stage gametocytes, the correlation between asexual blood stage and immature and late-stage gametocyte antiplasmodium activity is generally poor. A recent study focused on understanding the differences between asexual blood stage and gametocyte activity profiles for a range of antimalarial compounds. This study showed that gametocytocidal activity for three closely related *Plasmodium* PI4Kβ inhibitors with distinct physicochemical properties (MMV390048, UCT594 and UCT943) correlated with drug accumulation in gametocytes rather than target abundance.^21^ Further optimization, to ensure that compounds can effectively enter gametocytes via lipid-diffusion mediated uptake, will be required for the development of equipotent dual-active compounds.

### Microsomal Metabolic Stability

Selected compounds exhibiting high in vitro antiplasmodium activity (IC_50_ < 0.5 μM) and low cytotoxicity (CHO SI > 100) were progressed to in vitro microsomal metabolic stability testing in human liver microsomes (HLM), rat liver microsomes (RLM), and mouse liver microsomes (MLM). For this study, a single-point microsomal metabolic stability assay at a drug concentration of 0.1 μM was performed using liver microsome preparations from the three species of interest.^22^

From the results, four of five compounds assayed showed good microsomal metabolic stability across all species (> 85%; Table 5). Compounds **25**, **32**, and **34** displayed high stability (>90%) across the three species translating to a long half-life ( t12 ) of >150 min and low hepatic extraction ratio (*E*_H_) in human liver microsomal preparations. According to the percentage parent drug remaining after 30 min incubation, regio-isomerism appeared to impact microsomal stability as **19** (H/R/M = 89.2/87.0/85.5%) was less stable than **25** (H/R/M = 94.7/93.5/97.6%) across the three species. The di-chlorinated analogue **20** displayed moderate stability in RLMs (68.2% remaining after 30 min) and MLMs (59.7% remaining after 30 min), although it displayed high stability in HLMs (95.9% remaining after 30 min), suggesting species differences in metabolism.

**Table 5:** Microsomal Metabolic Stability and hERG Profiles for Selected Front-Runner Compounds

	microsomal metabolic stability			^e^hERG	
code	% rem. after 30 min^a^H/^b^R/^c^M	projected t12 ^d^ (min)	hepatic extraction ratio (*E*_H_)^a^H/^b^R/^c^M	IC_50_ (μM)	IC_20_ (μM)
**19**	89/87/86	>150/146.2/130.7	<0.42/<0.3/0.4	>30^f^	-
**20**	96/68/60	>150/54/40	<0.4/0.4/0.7	>30^f^	-
**25**	95/94/98	>150/>150/>150	<0.42/<0.3/<0.33	-	-
**32**	97/95/95	>150/>150/>150	<0.42/<0.3/<0.33	21.62	5.07
**34**	97/98/96	>150/>150/>150	<0.42/<0.3</0.33	78.99	8.61
**37**	-	-	-	13.74^g^	6.49
**sapanisertib**				175 ^23^	

a H = Human liver microsomes.

b R = Rat liver microsomes.

c M = Mouse liver microsomes.

d t12 = half-life.

e E-4031 used as reference compound at a concentration of 100 nM, with the hERG tail current blockade of 5.22 ± 0.95% relative remaining current for *n* = 2 experiments.

f Remaining current at highest concentration >70%, data obtained by extrapolation.

g Remaining current at highest concentration 50–70%; “-” = data not evaluated.

### In Vitro hERG Inhibitory Activity

To further evaluate potential compound safety, selected analogues were evaluated for cardiotoxicity risk by assessing inhibition of the human *ether-a-go-go-*related gene (hERG) channel stably expressed in the CHO cell line (Table 5). As with the parent compound sapanisertib (hERG IC_50_ = 175 μM)^23^ the compounds showed no hERG channel liability with IC_50_ values greater than 10 μM. All the tested compounds possessing the additional aromaticity including the pyridyls exhibited a clean profile. Pyridyl analogue **34** displayed low activity against the channel with IC_50_ of 79 μM. Lower inhibitory determinations (IC_20_) were also evaluated with modest activity against the cardiac channel obtained for **32**, **34**, and **37** with respective hERG IC_20_ values of 5.1, 8.6, and 6.5 μM. This further confirmed a low risk of hERG channel-related cardiotoxicity.

### In Vivo Efficacy Studies in P. berghei-Infected Mice

Compounds **19**, **20**, and **25** with potent in vitro antiplasmodium activity (*Pf*NF54 IC_50_ < 0.10 μM), low cytotoxicity (SI values 99 to >1250), no hERG channel liability risk (hERG IC_50_ > 10 μM), and good microsomal metabolic stability in microsomal preparations across species were assessed for their in vivo efficacy in the rodent-adapted P. berghei mouse model of malaria. Despite displaying potent dual PI4Kβ and PKG inhibition (*Pv*PI4Kβ IC_50_ < 0.01 μM; *Pf*PKG < 1 μM), most of the pyridyl analogues tested displayed a higher tendency for cross-resistance against *Pf*K1 and unfavorable cytotoxicity profile, hence were not progressed for efficacy studies. Four consecutive daily doses of 50 mg kg^–1^ of compound **19**, **20** and **25** resulted in 80, 44, and <40% reduction in parasitemia, respectively, relative to untreated mice (Table 6). However, despite the high efficacy of **19** in this model, curative effects were not observed at this dose since the mouse had a mean survival of 6 days.

**Table 6:**
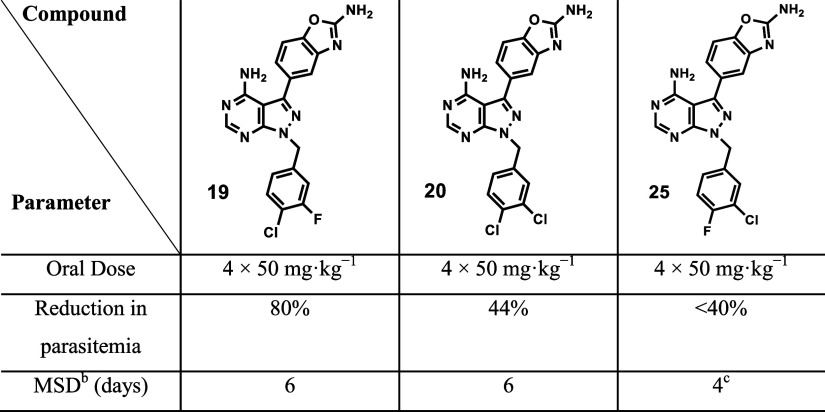
In Vivo Efficacy in P. berghei-Infected Mice at an Oral Dose of 4 × 50 mg kg^–1^ for Each Compound^a^

a Chloroquine (CQ) was used as the reference drug, resulting into 99.9% reduction in parasitemia at an oral dose of 4 × 30 mg kg^–1^, with a mouse mean survival of 24 days.

b MSD = mean survival days.

c Mice were euthanized on day 4 to prevent inevitable death due to increasing parasitemia.

### In Vivo Pharmacokinetic Studies

When administered intravenously, the blood clearance (CL_b_) of **19** was low in mice (Table 7), with a value less than 30% of hepatic blood flow. The oral exposure of **19** was 16% following an oral dose of 1 mg/kg.

**Table 7:** In Vivo Pharmacokinetic Parameters for **19** in Healthy BALB/c Mice

	19	
parameters^a^	i.v	p.o.
*C*_max_ (μM)		0.1
*t*_1/2_ (h)	1.4 (0.1)	
CL_b_ (mL/min/kg)	28.6 (6.0)	
AUC_0‑inf_ (min μM)	337 (30)	11 (2)
*V*_ss_ (L/kg)	1.8 (0.4)	
*F* (%)		16 (1)
*F*_abs_		0.21

a In vivo mouse pharmacokinetic parameters calculated from non-compartmental analysis of intravenous dosing at 0.5 mg/kg and oral dosing at 1 mg/kg. Data represented as mean (SD); *C*_max_, maximum concentration; *t*_1/2_, elimination half-life; *V*_ss_, apparent volume of distribution at steady state; CL_b_, whole-blood clearance; AUC_0‑inf_, area under the curve from time 0 extrapolated to infinite time; *F*, bioavailability; *F*_abs_, fraction absorbed.

Further to this, comparing the oral exposure of **19** in healthy BALB/c mice against the in vitro NF54 IC_50_ (Figure 5) supports the 80% reduction in parasitemia observed in the P. berghei model following four consecutive doses of 50 mg/kg.

**Figure 5:**
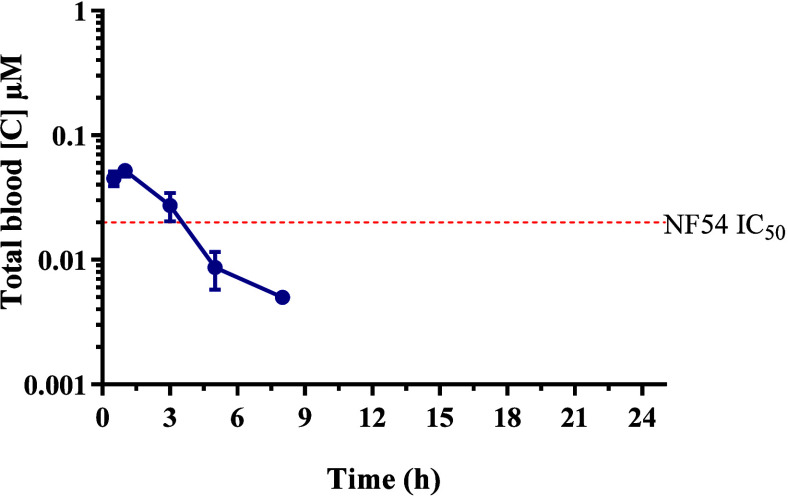
Whole-blood concentration-versus-time profile following oral administration of **19** to healthy BALB/c mice. The dashed line represents the in vitro NF54 IC_50_.

## Conclusions

In conclusion, this study identified new analogues based on sapanisertib with potent antiplasmodium and *Plasmodium* PI4Kβ inhibitory activity. Several analogues also showed nanomolar *Pf*PKG inhibition. The frontrunner benzyl analogue **19**, was found to be a dual inhibitor of *Pv*PI4Kβ (IC_50_ ≤ 0.001 μM) and *Pf*PKG (IC_50_ = 0.22 μM) while analogues **20** and **25** were selective for PI4Kβ (PI4Kβ IC_50_ = 0.001 and 0.004 μM, respectively) relative to *Pf*PKG (IC_50_ > 10 μM). Progression of these analogues to in vivo proof-of-concept resulted in high to moderate suppression of parasitemia in the P. berghei mouse model of infection. Analogue **19** displayed the highest efficacy with 80% reduction in parasitemia in the model with a mean survival timeframe of 6 days. However, these compounds and most active analogues displayed sub-optimal intrinsic solubility, a parameter that will require optimization. The kinase studies also showed that pyridyl analogues have a higher potential to dually target the *Plasmodium* proteins of interest. However, the compounds showed lower antiplasmodium potency, cytotoxicity risk and a higher propensity for cross-resistance against the multi-drug *Pf*K1 strain. While PI4Kβ was shown to be the primary efficacious target responsible for the asexual blood stage activity of sapanisertib,^7^ it cannot be ruled out that other *Plasmodium* kinase or non-kinase targets contribute to the observed antiplasmodium activity of some analogues. Particularly given that a chemoproteomic study showed that sapanisertib interacted with a range of kinase and non-kinase targets in *Plasmodium* cell lysate.^7^

Representative compounds tested against human PI4Kβ, maintained good selectivity for *Plasmodium* PI4Kβ, over the human orthologue. Analogues with significantly improved CHO cytotoxicity indices relative to sapanisertib were identified. Some compounds, such as **28** also showed late-stage gametocytocidal activity (*Pf* LGc IC_50_ = 0.52 μM). However, no reduction in human mTOR potency was observed for the tested compounds relative to sapanisertib (mTOR IC_50_ = 0.007 μM), the primary host target of sapanisertib for cancer treatment. While mitigating human kinase off-target activity is important for reducing the risk of toxicity in humans, it is worth noting that modulating the host immune response by targeting the human mTOR complex 1 has been proposed as a strategy for adjunctive host-directed therapy for cerebral malaria.^24^

Taken together, the work reveals the potential of a single molecule to simultaneously target multiple parasite kinases. This may offer benefits to potentially delay the onset of parasite resistance, increase clinical efficacy, and overcome the challenges of optimization of drug cocktails in clinic, as is often encountered in modern antimalarial drug discovery and development programs. However, to fully realize the benefit of dual PI4Kβ and PKG inhibitions for this series, further optimization will aim to improve potency against *Pf*PKG, de-risk human host kinase targets including mTOR, investigate selectivity over other human protein and lipid kinases, and optimize solubility for the delivery of potent drugs against malaria.

## Experimental Section

### Reagents, Solvents, Chromatography, and Instrumentation for Synthesis

All commercially available chemicals and solvents were purchased from either Sigma-Aldrich or Combi-Blocks. Unless otherwise stated, all solvents used were anhydrous. ^1^H NMR and ^13^C NMR spectra were acquired on either a Bruker AV 400 (^1^H 400.0, ^13^C 100.6 MHz), Varian Mercury 300 (^1^H 300.1, ^13^C 75.5 MHz) or Bruker Ascend 600 (^1^H 600.0, ^13^C 151 MHz) spectrometers. An Agilent LC-MS instrument comprising an Agilent 1260 Infinity Binary Pump, Agilent 1260 Infinity Diode Array Detector, Agilent 1290 Infinity Column Compartment, Agilent 1260 Infinity Autosampler, Agilent 6120 Quadrupole MS, and Peak Scientific Genius 1050 Nitrogen Generator, and fitted with an X-bridge (C18, 2.5 μm, 3.0 mm (ID) × 50 mm length) column maintained at 35 °C was used to monitor the progress of reactions including percent purity determination of the target compounds. For biological assays, target compounds were confirmed to have >95% purity by HPLC analysis. Analytical thin-layer chromatography (TLC) was performed on aluminum-backed silica-gel 60 F_254_ (70–230 mesh) plates. Flash column chromatography was performed with Merck silica-gel 60 (70–230 mesh). Chemical shifts (δ) are given in ppm downfield from tetramethylsilane (TMS) as the internal standard. Coupling constants, *J*, are recorded in hertz (Hz).

### Synthetic Procedures and Characterization for Representative Compounds

#### Synthesis of 5-(4,4,5,5-Tetramethyl-1,3,2-dioxaborolan-2-yl-)­benzo­[*d*]­oxazol-2-amine, **5**

A solution of 3-bromobenzo­[*d*]­oxazol-2-amine (0.50 g, 2.35 mmol) in dioxane (3.5 mL) was purged with N_2_ for 5 min and to this solution, bis­(pinacolato)­diboron (1.2 equiv), KOAc (3 equiv) and Pd­(dppf)­Cl_2_ (1 mol %) were sequentially added. The resulting mixture was then heated at 100 °C for 15 h with stirring. After completion of the reaction, the mixture was cooled to 20 °C, EtOAc added (50 mL) and the mixture filtered through Celite. Silica was added to the filtrate and concentrated in vacuo. The residue was then purified by flash CC (EtOAc: Hexane 0–55% v/v) to afford the crude product which was later triturated in diethyl ether to furnish the crucial intermediate. The product was obtained as an off-white solid (0.49 g, 80%); MP 161–162 °C; *R*_f_ (40% EtOAc/hexane) 0.36; ^1^H NMR (CD_3_OD, 400 MHz): δ_H_ 6.82 (d, *J* = 0.8 Hz, 1H), 6.68 (dd, *J* = 8.0 and 0.8 Hz, 1H), 6.47 (d, *J* = 8.0 Hz, 1H), and 0.55 (s, 12H). ^13^C NMR (101 MHz, DMSO-*d*_6_): δ_C_ 162.55, 149.84, 141.27, 126.90, 120.30, 115.70, 107.09, 82.86, and 22.97. HPLC-MS (APCI/ESI): (*m*/*z*) [M + H]^+^ = 261.1, calculated exact mass = 260.1332, purity 95%, *t*_R_ = 2.43 min.

#### Synthesis of 1*H*-Pyrazolo­[3,4-*d*]­pyrimidin-4-amine, **2**

A suspension of 5-amino-1*H*-pyrazole-4-carbonitrile (3.0 g, 27.75 mmol) and formamide (15 mL) was heated at 180 °C under N_2_ atmosphere for 15 h. After completion of the reaction, the mixture was cooled to 20 °C forming a brown precipitate, which was filtered off, washed with water (50 mL) and allowed to dry affording the product as a pale-brown solid (3.49 g, 93%); MP > 350 °C; *R*_f_ (15% MeOH/DCM) 0.3; ^1^H NMR (DMSO-*d*_6_, 400 MHz): δ_H_ 13.31 (broad s, 1H), 8.12 (s, 1H), 8.07 (s, 1H), and 7.56 (broad s, 2H). ^13^C NMR (101 MHz, DMSO-*d*_6_): δ_C_ 158.44, 156.26, 155.23, 133.05, and 100.04. HPLC-MS (APCI/ESI): (*m*/*z*) [M + H]^+^ = 136.0, calculated exact mass = 135.0545, purity = 95%, *t*_R_ = 0.14 min.

#### Synthesis of 3-Iodo-1*H*-pyrazolo­[3,4-*d*]­pyrimidin-4-amine, **3**

A suspension of 1*H*-pyrazolo­[3,4-*d*]­pyrimidin-4-amine (**2**) (1.50 g, 11.11 mmol) in anhydrous DMF (13 mL) was charged with *N*-iodosuccinimide (1.5 equiv). The resulting reaction mixture was then heated at 80 °C under N_2_ atmosphere for 15 h. After completion of the reaction, the mixture was then cooled to 20 °C forming a precipitate which was filtered off, washed with EtOH (50 mL), and allowed to dry affording the product as a pale yellow solid (2.58 g, 89%); *R*_f_ (10% MeOH/DCM) 0.6; ^1^H NMR (DMSO-*d*_6_, 400 MHz): δ_H_ 13.31 (broad s, 1H), 8.14 (s, 1H), 8.07 (s, 1H) and 7.16 (broad s, 2H). ^13^C NMR (101 MHz, DMSO-*d*_6_): δ_C_ 158.03, 156.48, 155.49, 102.96, and 90.10. HPLC-MS (APCI/ESI): (*m*/*z*) [M + H]^+^ = 262.0, calculated exact mass = 261.9511, purity 97%, *t*_R_ = 0.24 min.

### General Procedure for Synthesis of Intermediates **6a**–**38a**

A suspension of 3-iodo-1*H*-pyrazolo­[3,4-*d*] pyrimidin-4-amine (**3**) (1 equiv) and K_2_CO_3_ (2 equiv) in DMF (6 mL) was treated with the appropriate bromobenzyl derivative (1.2 equiv) or chlorobenzyl derivative and resulting mixture stirred at 30 °C or 50 °C, respectively for 2 h. The reaction mixture was then cooled to room temperature (20 °C), diluted with water (50 mL) and extracted with EtOAc (50 mL × 2). The combined organic layer was dried over anhydrous Na_2_SO_4_ and concentrated in vacuo to obtain the crude product, which was then purified on column or flash chromatography (0–8% MeOH/DCM) to furnish the required intermediates.

#### Characterization of Representative Intermediates

##### 1-(4-Chlorobenzyl)-3-iodo-1*H*-pyrazolo­[3,4-*d*]­pyrimidin-4-amine, **7a**

The compound was synthesized using the general procedure and a reaction mixture containing **3** (0.40 g, 1.53 mmol), K_2_CO_3_ (2 equiv) and 4-chlorobenzyl bromide (1.2 equiv) the product was obtained as a yellow powder (0.25 g, 43%); MP 246–247 °C; *R*_f_ (10% MeOH/DCM) 0.4; ^1^H NMR (DMSO-*d*_6_, 600 MHz): δ_H_ 8.22 (s, 1H), 7.35 (d*, J* = 9.0 Hz, 2H), 7.23 (d*, J* = 9.0, Hz 2H), and 5.46 (s, 2H). ^13^C NMR (DMSO-*d*_6_, 151 MHz): δ_C_ 158.21, 156.81, 154.00, 136.30, 132.84, 129.99 (2C), 129.07 (2C), 103.63, 90.17, and 49.75. HPLC-MS (APCI/ESI): (*m*/*z*) [M + H]^+^ = 385.9, calculated exact mass = 384.9591, purity > 99%, *t*_R_ = 2.51 min.

##### 1-(4-Fluorobenzyl)-3-iodo-1*H*-pyrazolo­[3,4-*d*]­pyrimidin-4-amine, **8a**

Using the general procedure and a reaction mixture containing **3** (0.40 g, 1.53 mmol), K_2_CO_3_ (2 equiv) and 4-fluorobenzyl bromide (1.2 equiv), the product was obtained as an off-white solid (0.26g, 44%); MP 194–195 °C *R*_f_ (10% MeOH/DCM) 0.6; ^1^H NMR (DMSO-*d*_6_, 600 MHz): δ_H_ 8.23 (s, 1H, H^6^), 7.28 (pseudo dd*, J* = 9.0 and 6.0 Hz, 2H, H^8^), 7.13 (t, *J* = 9.0 Hz, 2H, H^7^), and 5.46 (s, 2H, CH_2_). ^13^C NMR (DMSO-*d*_6_, 151 MHz): δ_C_ 162.08 (d, *J* = 244.6 Hz,^1^ *J*_C–F_, 1C), 158.27, 156.79, 153.92, 133.53 (d, *J* = 3.0 Hz, ^4^ *J*_C–F_, 1C), 130.28 (d, *J* = 7.6 Hz,^3^ *J*_C–F_, 2C), 115.88 (d, *J* = 22.7 Hz,^2^ *J*_C–F_, 2C), 103.62, 90.03, and 49.74. HPLC-MS (APCI/ESI): (*m*/*z*) [M + H]^+^ = 370.0, calculated exact mass = 369.9887, purity = 98%, *t*_R_ = 2.43 min.

### General Procedure for Synthesis of Target Compounds **6**–**38**

A solution of the appropriate *N*-alkylated intermediate (1 equiv) and 5-(4,4,5,5-tetramethyl-1,3,2-dioxaborolan-2-yl-)­benzo­[*d*]­oxazol-2-amine, (**5**) (1.3 equiv) in 7 mL dioxane/water mixture (3:1) was purged with N_2_ for 5 min. To the resulting mixture was sequentially added Na_2_CO_3_ (5 equiv) and Pd­(PPh_3_)_4_ (0.08 equiv), tightly sealed and then heated with stirring at 100 °C for 12 h. The reaction mixture was then cooled to 20 °C, filtered through Celite and the cake washed with 50% MeOH/DCM (50 mL). The organic filtrate was concentrated in vacuo and purified by column chromatography (0–12 % MeOH/DCM) to obtain the crude product, which was washed with MeOH and dried, to afford the expected product.

#### Characterization of Representative Target Compounds

##### 5-(4-Amino-1-(4-chloro-3-fluorobenzyl)-1*H*-pyrazolo­[3,4-*d*]­pyrimidin-3-yl)­benzo­[*d*]­oxazol-2-amine, **19**

The compound was synthesized using the general procedure 3 and a reaction mixture containing **19a** (250 mg, 0.62 mmol), the product was obtained as a brown solid (42 mg, 17%); MP 318–319 °C; *R*_f_ (10% MeOH/DCM) 0.45; ^1^H NMR (DMSO-*d*_6_, 600 MHz): δ_H_ 8.29 (s, 1H, H^6’^), 7.54 (pseudo t, *J* = 8.4 Hz, 1H, H^8^), 7.53 (s, 2H, NH_2_^2”^), 7.47 (d, *J* = 8.4 Hz, 1H, H^3^), 7.42 (d, *J* = 1.8 Hz, 1H, H^6^), 7.35 (dd, *J* = 10.1 and 1.8 Hz, 1H, H^9^), 7.25 (dd, *J* = 8.4 and 1.8 Hz, 1H, H^4^), 7.12 (dd, *J* = 8.4 and 1.8 Hz, 1H, H^7^) and 5.59 (s, 2H, CH_2_). ^13^C NMR (DMSO-*d*_6_, 151 MHz): δ_C_ 163.91, 158.70, 157.50 (d, *J* = 247.0 Hz,^1^ *J*_C–F_, 1C), 156.56, 154.82, 148.88, 145.46, 144.87, 139.36 (d, *J* = 7.6 Hz,^3^ *J*_C–F_, 1C), 131.33, 128.71, 125.28 (d, *J* = 3.0 Hz, ^3^ *J*_C–F_, 1C), 120.95, 119.08 (d, *J* = 16.6 Hz,^2^ *J*_C–F_, 1C), 116.48 (d, *J* = 21.1 Hz,^2^ *J*_C–F_, 1C), 115.49, 109.36, 97.90, and 49.16. HPLC-MS (APCI/ESI): purity 98%, *t*_R_ = 2.50 min, (m/z) [M + H]^+^ = 410.0.

##### 5-(4-Amino-1-(3,4-dichlorobenzyl)-1*H*-pyrazolo­[3,4-*d*]­pyrimidin-3-yl)­benzo­[*d*]­oxazol-2-amine, **20**

The compound was synthesized using the general procedure and a reaction mixture containing **20a** (0.35 g, 0.83 mmol), the product was obtained as an off-white solid (0.13 g, 37%); MP 288–289 °C; *R*_f_ (10% MeOH/DCM) 0.5; ^1^H NMR (DMSO-*d*_6_, 600 MHz): δ_H_ 8.26 (s, 1H, H^6’^), 7.57 (d, *J* = 1.8 Hz, 1H, H^9^), 7.56 (d, *J* = 8.4 Hz, 1H, H^8^), 7.50 (s, 2H, NH_2_^2”^), 7.44 (d, *J* = 8.4 Hz, 1H, H^3^), 7.39 (d, *J* = 1.8 Hz, 1H, H^6^), 7.22 (dd, *J* = 8.4 and 1.8 Hz, 1H, H^7^), 7.21 (dd, *J* = 7.8 and 1.8 Hz, 1H, H^4^), and 5.55 (s, 2H, CH_2_). ^13^C NMR (DMSO-*d*_6_, 151 MHz): δ_C_ 163.91, 158.69, 156.56, 154.81, 148.89, 145.47, 144.88, 138.74, 131.54, 131.33, 130.75, 130.12, 128.69, 128.43, 120.93, 115.47, 109.36, 97.89, and 48.99. HPLC-MS (APCI/ESI): purity 99%, *t*_R_ = 2.56 min, (*m*/*z*) [M + H]^+^ = 426.0.

##### 5-(4-Amino-1-(3-chloro-4-fluorobenzyl)-1*H*-pyrazolo­[3,4-*d*]­pyrimidin-3-yl)­benzo­[*d*]­oxazol-2-amine, **25**

The compound was synthesized using the general procedure and a reaction mixture containing **25a** (250 mg, 0.62 mmol), and the product as obtained as an off-white solid (68 mg, 27%); m.p. 285–286 °C; *R*_f_ (10% MeOH/DCM) 0.39; ^1^H NMR (DMSO-*d*_6_, 600 MHz): δ_H_ 7.52 (dd, *J* = 7.2 and 2.4 Hz, 1H, H^9^), 7.48 (s, 2H, NH_2_^2”^), 7.42 (dd, *J* = 7.8 and 0.6 Hz, 1H, H^3^), 7.37 (dd, *J* = 1.8 and 0.6 Hz, 1H, H^6^), 7.33 (pseudo t, *J* = 8.4 Hz, H^8^), 7.26 (ddd, *J* = 8.4, 4.8, and 2.4 Hz, 1H, H^7^), 7.20 (dd, *J* = 7.8 and 1.8 Hz, 1H, H^4^), and 5.52 (s, 2H, CH_2_). ^13^C NMR (DMSO-*d*_6_, 151 MHz): δ_C_ 163.89, 158.67, 157.07 (d, *J* = 246.1 Hz,^1^ *J*_C–F_, 1C), 156.53, 154.72, 148.86, 145.37, 144.86, 135.52 (d, *J* = 4.5 Hz,^3^ *J*_C–F_, 1C), 130.24, 128.89 (d, *J* = 7.6 Hz, ^3^ *J*_C–F_, 1C), 128.70, 120.91, 119.86 (d, *J* = 18.1 Hz,^2^ *J*_C–F_, 1C), 117.56 (d, *J* = 21.1 Hz,^2^ *J*_C–F_, 1C), 115.45, 109.33, 97.87, and 48.96. HPLC-MS (APCI/ESI): purity 98%, *t*_R_ = 2.50 min, (*m/z*) [M + H]^+^ = 410.0.

##### 5-(4-Amino-1-(4-chlorobenzyl)-1*H*-pyrazolo­[3,4-*d*]­pyrimidin-3-yl)­benzo­[*d*]­oxazol-2-amine, **7**

The compound was synthesized using the general procedure and a reaction mixture containing **7a** (0.15 g, 0.39 mmol), the product was obtained as an off-white solid (64 mg, 42%); MP 292–294 °C; *R*_f_ (100% EtOAc) 0.6; ^1^H NMR (600 MHz, DMSO-*d*_6_): δ_H_ 8.27 (s, 1H), 7.52 (s, 2H), 7.46 (d, *J* = 8.1 Hz, 1H), 7.40 (d, *J* = 1.7 Hz, 1H), 7.39 (d, *J* = 9.0 Hz, 2H), 7.32 (d, *J* = 9.0 Hz, 2H), 7.23 (dd, *J* = 8.1 and 1.7 Hz, 1H), and 5.55 (s, 2H). ^13^C NMR (DMSO-*d*_6_, 151 MHz): δ_C_ 163.90, 158.67, 156.48, 154.72, 148.85, 145.23, 144.87, 136.67, 132.68, 129.98 (2C), 129.02 (2C), 128.77, 120.92, 115.46, 109.34, 97.85, and 49.51. HPLC-MS (APCI/ESI): (*m*/*z*) [M + H]^+^ = 392.0, calculated exact mass = 391.0948, purity 96%, *t*_R_ = 2.49 min.

##### 5-(4-Amino-1-(4-fluorobenzyl)-1*H*-pyrazolo­[3,4-*d*]­pyrimidin-3-yl)­benzo­[*d*]­oxazol-2-amine, **8**

The compound was synthesized using the general procedure and a reaction mixture containing **8a** (0.20 g, 0.54 mmol), the product was obtained as a brown solid (0.11 g, 54%); MP 298–300 °C; *R*_f_ (10% MeOH/DCM) 0.4; ^1^H NMR (DMSO-*d*_6_, 600 MHz): δ_H_ 8.26 (s, 1H), 7.49 (s, 2H), 7.43 (d, *J* = 8.1 Hz, 1H), 7.38 (d, *J* = 1.8 Hz, 1H), 7.34 (dd, *J* = 8.4 and 5.4 Hz, 2H), 7.21 (dd, *J* = 7.8 and 1.8 Hz, 1H), 7.13 (t, *J* = 9.0 Hz, 2H), and 5.52 (s, 2H). ^13^C NMR (DMSO-*d*_6_, 151 MHz): δ_C_ 163.90, 162.82 (d, *J* = 241.6 Hz,^1^ *J*_C–F_, 2C), 158.67, 156.46, 154.65, 148.84, 145.14, 144.87, 133.90, 130.27 (d, *J* = 9.1 Hz,^3^ *J*_C–F_, 2C), 128.81, 120.92, 115.88 (d, *J* = 21.1 Hz,^2^ *J*_C–F_, 2C), 115.74, 109.33, 97.86, and 49.48. HPLC-MS (APCI/ESI): (*m*/*z*) [M + H]^+^ = 376.1, calculated exact mass = 375.1244, purity 98%, *t*_R_ = 2.42 min.

## Supplementary Material

















## Notes

Additional synthetic methods, characterization data, LC-MS and ^1^H NMR spectra for target compounds (**6**, **9–18, 21–24, 26–38**, and related carboxamides and sulfonamides); methods for in vivo efficacy studies, in vitro asexual blood stage antiplasmodium evaluation, solubility determination, in vitro *Pv*PI4Kβ, *Pf*PKG, *Hu*PI4Kβ, and *Hu*mTOR inhibition assays; hERG inhibition, cytotoxicity assays, and in silico docking against the target enzymes (PDF)
Molecular formula strings (CSV)
In silico docking file 1, PfPI4K-9 (PDB)
In silico docking file 2, PfPI4K-10 (PDB)
In silico docking file 3, PfPI4K-19 (PDB)
In silico docking file 4, PvPKG-10 (PDB)
In silico docking file 5, PvPKG-14 (PDB)
In silico docking file 6, PvPKG-37 (PDB)

## Abbreviations

ACT: artemisinin combination therapy
APCI: atmospheric pressure chemical ionization
ATP: adenosine triphosphate
^13^C NMR: carbon-13 nuclear magnetic resonance
cGMP: cyclic guanidine monophosphate
CHO: Chinese hamster ovarian
COVID 19: coronavirus disease
iGc: immature gametocytes
DMF: *N*,*N*-dimethylformamide
DMSO: dimethyl sulfoxide
DMSO-*d*_6_: deuterated dimethyl sulfoxide
ESI: electrospray ionization
FDA: The United States Food and Drugs Administration
HPLC: high pressure liquid chromatography
PIK: phosphoinositide kinase
PI4K: phosphatidylinositol-4-kinase
hERG: human *ether-a-go-go-*related gene
IC_20_: concentration of a drug that is required for 20% inhibition in vitro
IC_50_: concentration of a drug that is required for 50% inhibition in vitro
LC-MS: liquid chromatography mass spectrometry
LGc: late-stage gametocytes
MP.: melting point
ND: not determined
*P. falciparum*: Plasmodium falciparum
*Pf*: Plasmodium falciparum
*Pf*SCID: Plasmodium falciparum severe combined immunodeficiency gamma mouse model
PKG: cyclic guanidine monophosphate (cGMP)-dependent protein kinase
PDB: protein data base
*R*_*f*_: retardation factor
SAR: structure–activity relationship
NIS: *N*-iodosuccinimide
SD: standard deviation
SEM: standard error of the mean
SI: selectivity index
TLC: thin layer chromatography
*po*: per oral
UV: ultraviolet
^1^H NMR: proton nuclear magnetic resonance
TMS: tetramethylsilane
t_r_: retention time
δ_H_: chemical shift in ^1^H NMR spectrum
δ_C_: chemical shift in ^13^C NMR spectrum
*m/z*: mass to charge ratio
WHO: World Health Organization
