# Supplementary material for: Medicinal Chemistry Progression of Sapanisertib, the Anticancer and Dual Plasmodium Phosphatidylinositol 4‑Kinase Beta and cGMP-Dependent Protein Kinase Inhibitor, for Malaria
Source: J Med Chem. 2025 May 16;68(11):10757–70. doi: 10.1021/acs.jmedchem.4c02799 (PMC12169674; doi:10.1021/acs.jmedchem.4c02799)
Supplement: Supplementary file 1 [file jm4c02799_si_001.pdf]

Supplementary Information

Medicinal Chemistry Progression of Sapanisertib, the Anticancer and Dual *Plasmodium* Phosphatidylinositol 4-kinase beta and cGMP-dependent Protein Kinase Inhibitor, for Malaria

Samuel Gachuhi,[†] Stephanie Kamunya,[†] Stephen Fienberg,[∞] Lynn Wambua,[†,Ω] Nicolaas Salomane,[∞] Godfrey Mayoka,[†] Dale Taylor,[∞] Dina Coertzen,[‡] Mariette van der Watt,[‡] Janette Reader,[‡] Lyn-Marié Birkholtz,[‡, ¥] Sergio Wittlin,[ǁ,§] Liezl Krugmann,[∞] Lauren B. Coulson*,[∞,Ω] and Kelly Chibale*[,†,∞,Ω,Π]

[†]Department of Chemistry, University of Cape Town, Rondebosch 7701, Cape Town, South Africa.

[∞]Holistic Drug Discovery and Development (H3D) Centre, University of Cape Town, Rondebosch 7701, Cape Town, South Africa.

[Ω]Institute of Infectious Disease and Molecular Medicine, University of Cape Town, Observatory 7925, Cape Town, South Africa.

[‡]Department of Biochemistry, Genetics and Microbiology, Institute for Sustainable Malaria Control, University of Pretoria, Hatfield 0028, Pretoria, South Africa.

[¥]Department of Biochemistry, Stellenbosch University, Matieland, Stellenbosch, 7602, South Africa

[ǁ]Swiss Tropical and Public Health Institute, Kreuzstrasse 2, 4123 Allschwil, Switzerland.

[§]University of Basel, 4001 Basel, Switzerland.

[Π]South African Medical Research Council Drug Discovery and Development Research Unit, University of Cape Town, Rondebosch 7701, Cape Town, South Africa.

Corresponding author

*(L.B.C.) E-mail: lauren.coulson@uct.ac.za. Phone: +27-21-6505166. Fax: +27-21-6505195.

*(K.C.) E-mail: kelly.chibale@uct.ac.za. Phone: +27-21-6502553. Fax: +27-21-6505195.

Table of Contents

Table of Contents 2

1. Compound Synthesis and Characterization 3

2. ^{1}H, ^{13}C NMR and LC MS Spectra of Frontrunner Compounds and Key Intermediates 15

3. *In silico* Molecular Docking 26

4. Biological and Solubility Evaluation 27

   In vitro Asexual Blood Stage Antiplasmodium [^{3}H]-Hypoxanthine Incorporation Assay *27*

   In vitro Luciferase Reporter Gametocytocidal Assay *27*

   In vitro P. vivax PI4Kβ Inhibition Assay *27*

   In vitro P. falciparum PKG Inhibition Assay *28*

   Cytotoxicity in the Chinese Hamster Ovarian Cell Line *28*

   In vitro Microsomal Metabolic Stability *29*

   In vitro hERG Inhibition Assay *30*

   Solubility using HPLC-based DMSO "dry-down" method *30*

   In vivo Efficacy Studies in P. berghei-infected mice *31*

   In Vivo Pharmacokinetic Studies *31*

5. Supplementary Tables for Biological, Enzymatic and Solubility Data 33

6. Associated References 36

1. Compound Synthesis and Characterization

5-(4-Amino-1-benzyl-1*H*-pyrazolo[3,4-*d*]pyrimidin-3-yl)benzo[*d*]oxazol-2-amine, 6. Using the general procedure 2 and a reaction mixture containing **6a** (0.31 g, 0.88 mmol), the product was obtained as a brown solid (0.11 g, 36%); MP 287–289°C; R_f (8% MeOH/DCM) 0.4; ^{1}H-NMR (DMSO-d_6, 400 MHz): δ_H 8.29 (s, 1H, $H^{6'}$), 7.51 (s, 2H, $NH_2^{2''}$), 7.46 (d, J = 8.0 Hz, 1H, H^3), 7.42 (d, J = 2.0 Hz, 1H, H^6), 7.36–7.27 (m, 5H, $H^{7,8,9}$), 7.25 (dd, J = 8.0 and 2.0 Hz, 1H, H^4), and 5.56 (s, 2H, CH_2). ^{13}C-NMR (DMSO-d_6, 101 MHz): δ_H 163.92, 158.68, 156.43, 154.74, 148.86, 145.05, 144.90, 137.71, 129.00 (2C), 128.89, 128.08 (2C), 127.98, 120.93 (1C), 115.48, 109.33, 97.88, and 50.26. HPLC-MS (APCI/ESI): purity 99%, t_R = 2.40 min, (*m/z*) $[M+H]^+$ = 358.1.

5-(4-Amino-1-(4-(trifluoromethyl)benzyl)-1*H*-pyrazolo[3,4-*d*]pyrimidin-3-yl) benzo[*d*]oxazol-2-amine, 9. Pale yellow solid (0.11 g, 37%); m.p. 307–308°C; R_f (8% MeOH/DCM) 0.4; ^{1}H-NMR (DMSO-d_6, 600 MHz): δ_H 8.28 (s, 1H, $H^{6'}$), 7.70 (d, J = 8.1 Hz, 2H, H^8), 7.52 (s, 2H, $NH_2^{2''}$), 7.49 (d, J = 8.1 Hz, 2H, H^7), 7.46 (d, J = 8.1 Hz, 1H, H^3), 7.42 (d, J = 1.7 Hz, 1H, H^6), 7.24 (dd, J = 8.1 and 1.7 Hz, 1H, H^4), and 5.67 (s, 2H, CH_2). ^{13}C-NMR (DMSO-d_6, 151 MHz): δ_C 163.91, 158.70, 156.56, 154.88, 148.88, 145.42, 144.89, 142.38, 132.01, 128.74 (2C), 128.53 (q, J = 31.7 Hz, $^2J_{C\text{-}F}$, 1C), 125.97 (2C), 125.53 (q, J = 271.8 Hz, $^1J_{C\text{-}F}$, 1C), 120.93, 115.48, 109.34, 97.87, and 49.70. HPLC-MS (APCI/ESI): purity 98%, t_R = 2.53 min, (*m/z*) $[M+H]^+$ = 426.1.

5-(4-Amino-1-(4-aminobenzyl)-1*H*-pyrazolo[3,4-*d*]pyrimidin-3-yl)benzo[*d*]oxazol-2-amine, 10. Off-white solid (25 mg, 17%); m.p. 282–283°C; R_f (15% MeOH/DCM) 0.45; ^{1}H-NMR (600 MHz, DMSO-d_6) δ_H 8.23 (s, 1H, $H^{6'}$), 7.47 (s, 2H, $NH_2^{2''}$), 7.41 (d, J = 8.4 Hz, 1H, H^3), 7.35 (d, J = 1.8 Hz, 1H, H^6), 7.18 (dd, J = 8.4 and 1.8 Hz, 1H, H^4), 6.99 (d, J = 8.4 Hz, 2H, H^7), 6.45 (d, J = 8.4 Hz, 2H, H^8), 5.30 (s, 2H, CH_2), and 4.98 (s, 2H, benzyl NH_2). ^{13}C-NMR (DMSO-d_6, 151 MHz): δ_C 163.86, 158.56, 156.21, 154.28, 148.74, 148.65, 144.83, 144.52, 129.34 (2C), 129.00, 124.50, 120.88, 115.41, 114.11 (2C), 109.29, 97.79, and 50.15. HPLC-MS (APCI/ESI): purity 95%, t_R = 2.20 min, (*m/z*) $[M+H]^+$ = 373.1.

5-(4-Amino-1-(3-flourobenzyl)-1*H*-pyrazolo[3,4-*d*]pyrimidin-3-yl)benzo[*d*]oxazol-2-amine, 11. Off-white solid (0.12 g, 34%); m.p. 305–307°C; R_f (10% MeOH/DCM) 0.5; ^{1}H-NMR (DMSO-d_6, 400 MHz): δ_H 8.29 (s, 1H, $H^{6'}$), 7.51 (s, 2H, $NH_2^{2''}$), 7.47 (d, J = 8.4 Hz, 1H, H^3), 7.43 (d, J = 1.6 Hz, 1H, H^6), 7.41–7.35 (m, 1H, H^8), 7.25 (dd, J = 8.4 and 2.0 Hz, 1H, H^4), 7.13–7.09 (m, 3H, $H^{7,9,10}$), and 5.59 (s, 2H, CH_2). ^{13}C-NMR (DMSO-d_6, 101 MHz): δ_C 163.93, 163.80 (d, J = 244.4 Hz, $^1J_{C\text{-}F}$), 158.70, 156.51, 154.82, 148.89, 145.30, 144.90, 140.52 (d, J = 8.1 Hz, $^3J_{C\text{-}F}$, 1C), 131.13 (d, J = 8.1 Hz, $^3J_{C\text{-}F}$, 1C), 128.80, 124.07 (d, J = 2.0 Hz, $^4J_{C\text{-}F}$, 1C), 120.95, 115.49, 114.95 (d, J = 5.1 Hz, $^2J_{C\text{-}F}$, 1C), 114.74 (d, J = 6.1 Hz, $^2J_{C\text{-}F}$, 1C), 109.35, 97.91, and 49.66. HPLC-MS (APCI/ESI): purity 99%, t_R = 2.00 min, (*m*/*z*) $[M+H]^+$ = 376.1.

5-(4-Amino-1-(3-(trifluoromethyl)benzyl)-1*H*-pyrazolo[3,4-*d*]pyrimidin-3-yl)benzo[*d*]oxazol-2-amine, 12. Off-white solid (0.14 g, 35%); m.p. 281–283°C; R_f (10% MeOH/DCM) 0.6; ^{1}H-NMR (DMSO-d_6, 600 MHz): δ_H 9.12 (s, 1H, $H^{6'}$), 8.53–8.52 (m, 1H, H^{10}), 8.48–8.46 (m, 1H, H^9), 8.41–8.36 (m, 2H, $H^{7,8}$), 8.34 (s, 2H, $NH_2^{2''}$), 8.28 (dd, J = 7.8 and 0.6 Hz, 1H, H^3), 8.22 (dd, J = 1.8 and 0.6 Hz, 1H, H^6), 8.05 (dd, J = 8.4 and 1.8 Hz, 1H, H^4), and 6.49 (s, 2H, CH_2). ^{13}C-NMR (DMSO-d_6, 151 MHz): δ_C 164.73, 159.53, 157.38, 155.67, 149.70, 146.24, 145.71, 139.92, 132.99, 131.07, 130.60 (q, J = 31.7 Hz, $^2J_{C\text{-}F}$, 1C), 129.54, 126.25 (q, J = 273.3 Hz, $^1J_{C\text{-}F}$, 1C), 125.64 (broad q, J = 3.17 Hz, $^3J_{C\text{-}F}$, 1C), 125.47 (q, J = 3.47 Hz, $^3J_{C\text{-}F}$, 1C), 121.72, 116.27, 110.18, 98.69, and 50.43. HPLC-MS (APCI/ESI): purity 99%, t_R = 2.51 min, (*m*/*z*) $[M+H]^+$ = 426.1.

5-(4-Amino-1-(3-chlorobenzyl)-1*H*-pyrazolo[3,4-*d*]pyrimidin-3-yl)benzo[*d*]oxazol-2-amine, 13. Off-white solid (0.13 g, 38%); m.p. 310–312°C; R_f (10% MeOH/DCM) 0.5; ^{1}H-NMR (DMSO-d_6, 600 MHz): δ_H 7.52 (s, 2H, $NH_2^{2''}$), 7.46 (d, J = 8.4 Hz, 1H, H^3), 7.41 (d, J = 1.8 Hz, 1H, H^6), 7.38 - 7.34 (m, 3H, $H^{8,9,10}$), 7.25–7.23 (m, 2H, $H^{4,7}$), 5.57 (s, 2H, CH_2); ^{13}C-NMR (DMSO-d_6, 151 MHz): δ_C 163.91, 158.70, 156.54, 154.79, 148.87, 145.32, 144.89, 140.13, 135.02, 133.56, 131.01, 128.75, 128.02, 127.89, 126.75, 120.92, 115.46, 109.36, 97.87, and 49.56. HPLC-MS (APCI/ESI): purity 96%, t_R = 2.50 min, (*m*/*z*) $[M+H]^+$ = 392.0.

5-(4-Amino-1-(3-aminobenzyl)-1*H*-pyrazolo[3,4-*d*]pyrimidin-3-yl)benzo[*d*]oxazol-2-amine, 14. Off-white solid (53 mg, 28%); m.p. 286–287°C; R_f (10% MeOH/ DCM) 0.45; ^{1}H-NMR (DMSO-d_6, 400 MHz): δ_H 8.28 (s, 1H, H$^{6'}$), 7.52 (s, 2H, NH$_2$$^{2''}$), 7.47 (d, J = 8.4 Hz, 1H, H^3), 7.42 (d, J = 1.6 Hz, 1H, H^6), 7.25 (dd, J = 8.0 and 1.6 Hz, 1H, H^4), 6.95 (t, J = 8.0 Hz, 1H, H^8), 6.48–6.43 (m, 3H, H^{7,9,10}), 5.38 (s, 2H, CH$_2$), and 5.06 (s, 2H, benzyl NH$_2$). ^{13}C-NMR (DMSO-d_6, 101 MHz): δ_H 163.91, 158.63, 156.32, 154.65, 149.28, 148.83, 144.88, 144.77, 138.24, 129.41, 128.99, 120.94, 115.50 (2C), 113.59, 113.28, 109.32, 97.83, and 50.50. HPLC-MS (APCI/ESI): purity 98%, t_R = 2.21 min, (*m*/*z*) [M+H]$^+$ = 373.1.

5-(4-Amino-1-(3-(dimethylamino)benzyl)-1*H*-pyrazolo[3,4-*d*]pyrimidin-3-yl)benzo[*d*] oxazol-2-amine, 15. Off-white solid (58 mg, 24%); m.p. 269–270°C; R_f (10% MeOH/DCM) 0.43; ^{1}H-NMR (DMSO-d_6, 400 MHz): δ_H 8.28 (s, 1H, H$^{6'}$), 7.49 (s, 2H, NH$_2$$^{2''}$), 7.46 (d, J = 8.4 Hz, 1H, H^3), 7.41 (d, J = 1.6 Hz, 1H, H^6), 7.24 (dd, J = 8.0 and 1.6 Hz, 1H, H^4), 7.10 (t, J = 8.0 Hz, 1H, H^8), 6.73 (broad t, J = 2.4 Hz, 1H, H^{10}), 6.62 (dd, J = 8.0 and 2.4 Hz, 1H, H^9), 6.53 (broad d, J = 7.6 Hz, 1H, H^7), 5.48 (s, 2H, CH$_2$), and 2.85 (s, 6H, CH$_3$). ^{13}C-NMR (DMSO-d_6, 101 MHz): δ_H 163.92, 158.64, 156.34, 154.72, 150.98, 148.83, 144.88, 144.84, 138.33, 129.52, 128.96, 120.90, 115.73, 115.44, 112.06, 112.03, 109.34, 97.86, 50.76 and 40.46 (2C). HPLC-MS (APCI/ESI): purity 99%, t_R = 2.45 min, (*m*/*z*) [M+H]$^+$ = 401.1.

5-(4-Amino-1-(2-flourorobenzyl)-1*H*-pyrazolo[3,4-*d*]pyrimidin-3-yl)benzo[*d*]oxazol-2-amine, 16. Off-white solid (0.13 g, 43%); m.p. 296–297°C; R_f (10% MeOH/DCM) 0.4; ^{1}H-NMR (DMSO-d_6, 600 MHz): δ_H 8.26 (s, 1H, H$^{6'}$), 7.49 (s, 2H, NH$_2$$^{2''}$), 7.43 (dd, J = 7.8 and 0.6 Hz, 1H, H^3), 7.37 (dd, J = 1.8 and 0.6 Hz, 1H, H^6), 7.34–7.31 (m, 1H, H^9), 7.20 (dd, J = 7.8 and 1.8 Hz, 1H, H^4), 7.20–7.19 (m, 1H, H^{10}), 7.18–7.17 (m, 1H, H^7), 7.12 (td, J = 7.8 and 1.2 Hz, 1H, H^8), and 5.59 (s, 2H, CH$_2$). ^{13}C-NMR (DMSO-d_6, 151 MHz): δ_C 163.90, 161.11 (d, J = 244.6 Hz, $^1J_{C\text{-}F}$, 1C) 158.66, 156.46, 154.85, 148.86, 145.27, 144.87, 130.61 (d, J = 3.0 Hz, $^3J_{C\text{-}F}$, 1C), 130.33 (d, J = 7.6 Hz, $^3J_{C\text{-}F}$, 1C), 128.77, 125.08 (d, J = 3.0 Hz, $^4J_{C\text{-}F}$, 1C), 124.43 (d, J = 13.6 Hz, $^2J_{C\text{-}F}$, 1C), 120.92, 115.93 (d, J = 21.1 Hz, $^2J_{C\text{-}F}$, 1C), 115.46, 109.34, 97.80, and 43.89 (d, J = 3.0 Hz, $^3J_{C\text{-}F}$, 1C). HPLC-MS (APCI/ESI): purity >99%, t_R = 2.38 min, (*m*/*z*) [M+H]$^+$ = 376.1.

5-(4-Amino-1-(2-(chloromethyl)benzyl)-1*H*-pyrazolo[3,4-*d*]pyrimidin-3-yl)benzo[*d*] oxazol-2-amine, 17. Off-white solid (46 mg, 18%); m.p. 293–294°C; R_f (10% MeOH/ DCM) 0.50; ^{1}H-NMR (DMSO-d_6, 600 MHz): δ_H 8.26 (s, 1H, $H^{6'}$), 7.51 (s, 2H, $NH_2{}^{2''}$), 7.48 (dd, J = 8.4 and 1.2 Hz, 1H, H^{10}), 7.45 (d, J = 8.4 Hz, 1H, H^3), 7.40 (d, J = 1.8 Hz, 1H, H^6), 7.32 (td, J = 7.2 and 1.8 Hz, 1H, H^9), 7.26 (td, J = 7.2 and 1.2 Hz, 1H, H^8), 7.23 (dd, J = 8.4 and 1.8 Hz, 1H, H^4), 6.99 (dd, J = 7.8 and 1.8 Hz, 1H, H^7), and 5.64 (s, 2H, CH_2). ^{13}C-NMR (DMSO-d_6, 151 MHz): δ_C 163.92, 158.71, 156.51, 155.09, 148.88, 145.46, 144.88, 134.95, 132.33, 129.84 (2C), 129.80, 128.77, 127.94, 120.94, 115.49, 109.35, 97.83, and 47.73. HPLC-MS (APCI/ESI): purity 98%, t_R = 2.47 min, (m/z) $[M+H]^+$ = 392.0.

5-(4-Amino-1-(2-(trifluoromethyl)benzyl)-1*H*-pyrazolo[3,4-*d*]pyrimidin-3-yl)benzo[*d*] oxazol-2-amine, 18. Off-white solid (88 mg, 21%); m.p. 279–280°C; R_f (10% MeOH/ DCM) 0.46; ^{1}H-NMR (DMSO-d_6, 600 MHz): δ_H 8.28 (s, 1H, $H^{6'}$), 7.80 (d, J = 7.2 Hz, 1H, H^{10}), 7.59 (t, J = 7.2 Hz, 1H, H^8), 7.53 (s, 2H, $NH_2{}^{2''}$), 7.52 (overlapping t, J = 7.8 Hz, 1H, H^9), 7.47 (d, J = 7.8 Hz, 1H, H^3), 7.43 (d, J = 1.8 Hz, 1H, H^6), 7.26 (dd, J = 7.8 and 1.8 Hz, 1H, H^4), 6.94 (d, J = 7.8 Hz, 1H, H^7), and 5.76 (s, 2H, CH_2). ^{13}C-NMR (DMSO-d_6, 151 MHz): δ_H 163.92, 158.74, 156.66, 155.29, 148.91, 145.70, 144.89, 135.86, 133.46, 129.10, 128.69, 128.53, 126.58 (q, J = 30.4 Hz, $^2J_{C\text{-}F}$, 1C), 126.49 (q, J = 6.0 Hz, $^3J_{C\text{-}F}$, 1C), 124.81 (q, J = 274.1 Hz, $^1J_{C\text{-}F}$, 1C), 120.96, 115.51, 109.36, 97.89, and 46.67. HPLC-MS (APCI/ESI): purity 98%, t_R = 2.50 min, (m/z) $[M+H]^+$ = 426.1.

5-(4-Amino-1-(2,4-dichlorobenzyl)-1*H*-pyrazolo[3,4-*d*]pyrimidin-3-yl) benzo[*d*]oxazol-2-amine, 21. Off-white solid (0.10 g, 38%); m.p. 271–272°C; R_f (10% MeOH/DCM) 0.4; ^{1}H-NMR (DMSO-d_6, 600 MHz): δ_H 8.25 (s, 1H, $H^{6'}$), 7.64 (d, J = 2.4 Hz, 1H, H^6), 7.50 (s, 2H, $NH_2{}^{2''}$), 7.44 (d, J = 7.8 Hz, 1H, H^3), 7.38 (d, J = 1.2 Hz, 1H, H^9), 7.36 (dd, J = 8.4 and 2.4 Hz, 1H, H^8), 7.21 (dd, J = 7.8 and 1.8 Hz, 1H, H^4), 7.04 (d, J = 8.4 Hz, 1H, H^7), and 5.61 (s, 2H, -CH_2). ^{13}C-NMR (DMSO-d_6, 151 MHz): δ_C 164.81, 159.60, 157.44, 155.96, 149.79, 146.48, 145.77, 134.99, 134.46, 134.30, 132.25, 130.24, 129.58, 129.04, 121.83, 116.38, 110.25, 98.73, and 48.18. HPLC-MS (APCI/ESI): purity 99%, t_R = 2.56 min, (m/z) $[M+H]^+$ = 426.0.

5-(4-Amino-1-(4-chloro-2-fluorobenzyl)-1*H*-pyrazolo[3,4-*d*]pyrimidin-3yl)benzo[*d*] oxazol-2-amine, 22. Off-white solid (87 mg, 19%); m.p. 272–273°C; R_f (10% MeOH/ DCM) 0.32; ^{1}H-NMR (DMSO-d_6, 400 MHz): δ_H 8.29 (s, 1H, $H^{6'}$), 7.52 (s, 2H, $NH_2{}^{2''}$), 7.46 (overlapping d, J = 7.6 Hz, 1H, H^3), 7.45 (overlapping dd, J = 8.8 and 1.2 Hz, 1H, H^9), 7.40 (d, J = 1.6 Hz, 1H, H^6), 7.30–7.25 (m, 2H, $H^{7,8}$), 7.23 (dd, J = 8.4 and 2.0 Hz, 1H, H^4), and 5.60 (s, 2H, CH_2).^{13}C-NMR (DMSO-d_6, 101 MHz): δ_C 163.42, 159.73 (d, J = 249.2 Hz, $^1J_{C\text{-}F}$, 1C), 158.18, 156.02, 154.36, 148.39, 144.93, 144.37, 133.28 (d, J = 10.6 Hz, $^3J_{C\text{-}F}$, 1C), 131.50 (d, J = 4.5 Hz, $^3J_{C\text{-}F}$, 1C), 128.21, 124.89 (d, J = 3.0 Hz, $^4J_{C\text{-}F}$, 1C), 123.11 (d, J = 15.1 Hz, $^2J_{C\text{-}F}$, 1C), 120.44, 116.08 (d, J = 25.7 Hz, $^2J_{C\text{-}F}$, 1C), 114.97, 108.86, 97.33, and 43.04 (d, J = 4.5 Hz, $^4J_{C\text{-}F}$, 1C). HPLC-MS (APCI/ESI): purity 98%, t_R = 2.49 min, (m/z) $[M+H]^+$ = 410.0

5-(4-Amino-1-(2,4-difluorobenzyl)-1*H*-pyrazolo[3,4-*d*]pyrimidin-3-yl)benzo[*d*]oxazol-2-amine, 23. Off-white solid (84 mg, 23%); m.p. 294–295°C; R_f (10% MeOH/DCM) 0.48; ^{1}H-NMR (DMSO-d_6, 600 MHz): δ_H 8.29 (s, 1H, $H^{6'}$), 7.51 (s, 2H, $NH_2{}^{2''}$), 7.46 (d, J = 8.0 Hz, 1H, H^3), 7.40 (d, J = 1.6 Hz, 1H, H^6), 7.34 (td, J = 8.4 and 6.4 Hz, 1H, H^7), 7.26 (overlapping td, J = 9.4 and 1.6 Hz, 1H, H^9), 7.23 (dd, J = 8.0 and 1.6 Hz, 1H, H^4), 7.05 (td, J = 8.8, 7.6 and 2.0 Hz, 1H, H^8), and 5.58 (s, 2H, CH_2). ^{13}C-NMR (DMSO-d_6, 101 MHz): δ_C 163.93, 162.7 (d, J = 247.5 Hz), 160.47 (d, J = 237.4 Hz) 158.68, 156.49, 154.83, 148.90, 145.34, 144.90, 132.10 (dd, J = 5.1 Hz, $^3J_{C\text{-}F}$, 1C), 128.77, 120.93, 120.79 (dd, J = 11.1 and 4.0 Hz, 1C), 115.49, 112.17 (dd, J = 18.2 and 3.0 Hz, 1C), 109.34, 104.44 (t, J = 26.3 Hz, $^2J_{C\text{-}F}$, 1C), 97.86, and 43.48. HPLC-MS (APCI/ESI): purity >99%, t_R = 2.42 min, (m/z) $[M+H]^+$ = 394.1. (confirmed by HSQC and HMBC).

5-(4-Amino-1-(2-chloro-4-fluorobenzyl)-1*H*-pyrazolo[3,4-*d*]pyrimidin-3-yl)benzo[*d*] oxazol-2-amine, 24. Off-white solid (67 mg, 20%); m.p. 277–278°C; R_f (10% MeOH/DCM) 0.44; ^{1}H-NMR (DMSO-d_6, 400 MHz): δ_H 8.28 (s, 1H, $H^{6'}$), 7.50 (s, 2H, $NH_2{}^{2''}$), 7.48 (dd, J = 8.8 and 2.4 Hz, 1H, H^9), 7.46 (d, J = 8.0 Hz, 1H, H^3), 7.42 (d, J = 1.6 Hz, 1H, H^6), 7.24 (dd, J = 8.4 and 2.0 Hz, 1H, H^4), 7.18 (overlapping ddd, J = 13.6, 8.4 and 2.0 Hz, 1H, H^8), 7.17 (overlapping dd, J = 8.4 and 2.4 Hz, 1H, H^7), and 5.63 (s, 2H, CH_2). ^{13}C-NMR (DMSO-d_6, 101 MHz): δ_C 163.94, 161.81 (d, J = 248.5 Hz, $^1J_{C\text{-}F}$, 1C), 158.71, 156.52, 155.04, 148.91, 145.50, 144.89, 133.33 (d, J = 10.7 Hz, $^3J_{C\text{-}F}$, 1C), 131.68 (d, J = 9.2 Hz, $^3J_{C\text{-}F}$, 1C), 131.34 (d, J = 3.4 Hz, $^4J_{C\text{-}F}$, 1C), 128.75,

120.95, 117.16 (d, J = 25.4 Hz, $^{2}J_{C-F}$, 1C), 115.51, 115.11 (d, J = 21.4 Hz, $^{2}J_{C-F}$, 1C), 109.35, 97.87, and 47.20. HPLC-MS (APCI/ESI): purity 98%, t_R = 2.49 min, (m/z) $[M+H]^+$ = 410.0.

5-(4-Amino-1-(3,4-difluorobenzyl)-1*H*-pyrazolo[3,4-*d*]pyrimidin-3-yl)benzo[*d*]oxazol-2-amine, 26. Off-white solid (58 mg, 16%); m.p. 289–290°C; R_f (10% MeOH/ DCM) 0.35; ^{1}H-NMR (DMSO-d_6, 600 MHz): δ_H 8.24 (s, 1H, $H^{6'}$), 7.48 (s, 2H, $NH_2^{2''}$), 7.42 (d, J = 8.4 Hz, 1H, H^3),7.38 (d, J = 1.8 Hz, 1H, H^6), 7.37–7.32 (m, 2H, $H^{7,8}$), 7.20 (dd, J = 7.8 and 1.8 Hz, 1H, H^4), 7.11–7.08 (m, 1H, H^9), and 5.52 (s, 2H, CH_2). ^{13}C-NMR (DMSO-d_6, 101 MHz): δ_C 163.89, 158.67, 156.51, 154.72, 150.30 (dd, J = 55.9 and 12.1 Hz, 1C), 148.85, 148.67 (dd, J = 54.4 and 12.1 Hz, 1C), 145.35, 144.85, 135.32 (pseudo t, J = 4.5 Hz, 1C), 128.71, 124.96 (dd, J = 7.6 and 3.0 Hz, 1C), 120.92, 118.13 (d, J = 12.1 Hz, 1C), 117.21 (d, J = 12.1 Hz, 1C), 115.46, 109.33, 97.87, and 49.11. HPLC-MS (APCI/ESI): purity >99%, t_R = 2.43 min, (m/z) $[M+H]^+$ = 394.1.

5-(4-Amino-1-((6-(trifluoromethyl)pyridin-3-yl)methyl)-1*H*-pyrazolo[3,4-*d*]pyrimidin-3-yl)benzo[*d*]oxazol-2-amine, 27. Off-white solid (0.15 g, 36%); m.p. 299–300°C; R_f (12% MeOH/DCM) 0.5; ^{1}H-NMR (DMSO-d_6, 600 MHz): δ_H 8.75 (d, J = 1.8 Hz, 1H, H^9), 8.27 (s, 1H, $H^{6'}$), 7.92 (dd, J = 8.4 and 1.8 Hz, 1H, H^7), 7.85 (dd, J = 8.4 and 1.2 Hz, 1H, H^8), 7.50 (s, 2H, $NH_2^{2''}$), 7.44 (dd, J = 8.4 and 0.6 Hz, 1H, H^3), 7.40 (dd, J = 1.8 and 0.6 Hz, 1H, H^6), 7.22 (dd, J = 8.4 and 1.8 Hz, 1H, H^4), and 5.72 (s, 2H, CH_2). ^{13}C-NMR (DMSO-d_6, 101 MHz): δ_C 163.91, 158.72, 156.63, 154.93, 149.88, 148.91, 146.05 (q, J = 34.7 Hz, $^{2}J_{C-F}$, 1C), 145.71, 144.88, 137.98, 137.21, 128.63, 121.26 (broad q, J = 1.5 Hz, $^{3}J_{C-F}$, 1C), 121.13 (q, J = 274.8 Hz, $^{1}J_{C-F}$, 1C), 120.95, 115.51, 109.35, 97.97, and 47.42. HPLC-MS (APCI/ESI): purity 99%, t_R = 2.38 min, (m/z) $[M+H]^+$ = 427.1.

5-(4-Amino-1-((6-chloropyridin-3-yl)methyl)-1*H*-pyrazolo[3,4-*d*]pyrimidin-3-yl) benzo[*d*]oxazol-2-amine, 28. Brown solid (45 mg, 21%); m.p. 289–291°C; R_f (10% MeOH/DCM) 0.30; ^{1}H-NMR (DMSO-d_6, 600 MHz): δ_H 8.42 (dd, J = 2.4 and 0.6 Hz, 1H, H^9), 8.27 (s, 1H, $H^{6'}$), 7.75 (dd, J = 7.8 and 2.4 Hz, 1H, H^7), 7.51 (s, 2H, $NH_2^{2''}$), 7.47 (dd, J = 8.4 and 0.6 Hz, 1H, H^8), 7.45 (dd, J = 8.4 and 0.6 Hz, 1H, H^3), 7.40 (dd, J = 1.8 and 0.6 Hz, 1H, H^6), 7.22 (dd, J = 7.8 and 1.8 Hz, 1H, H^7), and 5.60 (s, 2H, CH_2). ^{13}C-NMR (DMSO-d_6, 151 MHz): δ_C 163.42, 158.21, 156.08, 154.31, 149.53, 149.15, 148.40, 145.04, 144.39, 139.29, 132.34, 128.18, 124.32, 120.44,

115.00, 108.85, 97.46, and 46.56. HPLC-MS (APCI/ESI): purity >99%, t_R = 2.56 min, (m/z) $[M+H]^+$ = 392.1.

5-(4-Amino-1-((6-flouropyridin-3-yl)methyl)-1*H*-pyrazolo[3,4-*d*]pyrimidin-3-yl) benzo[*d*]oxazol-2-amine, 29. Brown solid (55 mg, 24%); m.p. 275–276°C; R_f (10% MeOH/DCM) 0.30; ^{1}H-NMR (DMSO-d_6, 400 MHz): δ_H 8.30 (s, 1H, $H^{6'}$), 8.28–8.27 (broad m, 1H, H^9), 7.93 (td, J = 8.0 and 2.4 Hz, 1H, H^7), 7.50 (s, 2H, $NH_2^{2''}$), 7.46 (d, J = 8.0 Hz, 1H, H^3), 7.42 (d, J = 1.6 Hz, 1H, H^6), 7.24 (dd, J = 8.0 and 1.6 Hz, 1H, H^4), 7.15 (ddd, J = 8.8, 3.2 and 0.8 Hz, 1H, H^8) and 5.62 (s, 2H, CH_2). ^{13}C-NMR (DMSO-d_6, 101 MHz): δ_C 163.93, 162.97 (d, J = 236.3 Hz, $^1J_{C\text{-}F}$, 1C), 158.71, 156.55, 154.76, 148.91, 147.32 (d, J = 16.2 Hz, $^3J_{C\text{-}F}$, 1C), 145.46, 144.90, 142.30 (d, J = 8.1 Hz, $^3J_{C\text{-}F}$, 1C), 131.55 (d, J = 5.1 Hz, $^4J_{C\text{-}F}$, 1C), 128.73, 120.94, 115.51, 110.09 (d, J = 37.4 Hz, $^2J_{C\text{-}F}$, 1C), 109.34, 97.99, and 46.97. HPLC-MS (APCI/ESI): purity 99%, t_R = 2.25 min, (m/z) $[M+H]^+$ = 377.1.

5-(4-Amino-1-((6-methylpyridin-3-yl)methyl)-1*H*-pyrazolo[3,4-*d*]pyrimidin-3-yl) benzo[*d*]oxazol-2-amine, 30. Off-white solid (164 mg, 40%); m.p. 279–280°C; R_f (10% MeOH/ DCM) 0.39; ^{1}H-NMR (DMSO-d_6, 600 MHz): δ_H 8.42 (d, J = 2.4 Hz, 1H, H^9), 8.25 (s, 1H, $H^{6'}$), 7.55 (dd, J = 7.8 and 2.4 Hz, 1H, H^4), 7.48 (s, 2H, $NH_2^{2''}$), 7.42 (d, J = 7.8 Hz, 1H, H^3), 7.36 (d, J = 1.8 Hz, 1H, H^6), 7.19 (dd, J = 7.8 and 2.4 Hz, 1H, H^7), 7.16 (d, J = 7.8 Hz, 1H, H^8), 5.51 (s, 2H, CH_2), and 2.38 (s, 3H, CH_3). ^{13}C-NMR (DMSO-d_6, 151 MHz): δ_C 163.88, 158.64, 157.74, 156.45, 154.65, 148.83, 148.74, 145.21, 144.84, 136.36, 130.14, 128.75, 123.40, 120.90, 115.44, 109.31, 97.88, 47.68, and 24.12. HPLC-MS (APCI/ESI): purity 99%, t_R = 2.22 min, (m/z) $[M+H]^+$ = 373.1.

5-(4-Amino-1-(pyridin-4-ylmethyl)-1*H*-pyrazolo[3,4-*d*]pyrimidin-3-yl) benzo[*d*]oxazol-2-amine, 31. White solid (0.13 g, 39%); m.p. 299–300°C; R_f (10% MeOH/DCM) 0.4; ^{1}H-NMR (DMSO-d_6, 400 MHz): δ_H 8.52 (pseudo dd, J = 6.0 and 1.6 Hz, 2H, H^8), 8.28 (s, 1H, $H^{6'}$), 7.51 (s, 2H, $NH_2^{2''}$), 7.47 (d, J = 8.4 Hz, 1H, H^3), 7.44 (d, J = 1.6 Hz, 1H, H^6), 7.26 (dd, J = 8.4 and 1.6 Hz, 1H, H^4), 7.21 (pseudo dd, J = 6.0 and 1.6 Hz, 2H, H^7), and 5.62 (s, 2H, CH_2). ^{13}C-NMR (DMSO-d_6, 101 MHz): δ_C 163.93, 158.73, 156.59, 155.06, 150.32 (2C), 148.92, 146.46, 145.55, 144.91, 128.73, 122.69 (2C), 120.96, 115.52, 109.35, 97.92, and 49.13. HPLC-MS (APCI/ESI): purity 99%, t_R = 2.04 min, (m/z) $[M+H]^+$ = 359.1.

5-(4-Amino-1-((2-chloropyridin-4-yl)methyl)-1*H*-pyrazolo[3,4-*d*]pyrimidin-3-yl) benzo[*d*] oxazol-2-amine, 32. White solid (55 mg, 17%); m.p. 292–293°C; R$_f$ (10% MeOH/DCM) 0.41; ^{1}H-NMR (DMSO-d_6, 400 MHz): δ_H 8.34 (d, J = 5.2 Hz, 1H, H^8), 8.27 (s, 1H, H$^{6'}$), 7.51 (s, 2H, NH$_2$$^{2''}$), 7.46 (d, J = 8.4 Hz, 1H, H^3), 7.43 (d, J = 1.6 Hz, 1H, H^6), 7.36 (d, J = 1.2 Hz, 1H, H^9), 7.25 (dd, J = 8.0 and 1.6 Hz, 1H, H^4), 7.18 (dd, J = 5.2 and 1.2 Hz, 1H, H^7), and 5.63 (s, 2H, CH$_2$). ^{13}C-NMR (DMSO-d_6, 101 MHz): δ_C 163.94, 158.60, 156.46, 155.05, 151.00, 150.72, 150.54, 148.96, 145.88, 144.86, 128.59, 123.04, 122.13, 120.99, 115.53, 109.40, 97.96, and 48.66. HPLC-MS (APCI/ESI): purity 99%, t$_R$ = 2.30 min, (m/z) [M+H]$^+$ = 393.1.

5-(4-Amino-1-(pyridin-3-ylmethyl)-1*H*-pyrazolo[3,4-*d*]pyrimidin-3-yl) benzo[*d*]oxazol-2-amine, 33. Off-white solid (0.15 g, 50%); m.p. 286–287°C; R$_f$ (15% MeOH/DCM) 0.6; ^{1}H-NMR (DMSO-d_6, 400 MHz): δ_H 8.59 (dd, J = 2.4 and 1.2 Hz, 1H, H^{10}), 8.50 (dd, J = 4.8 and 1.6 Hz, 1H, H^9), 8.30 (s, 1H, H$^{6'}$), 7.71 (ddd, J = 7.9, 2.3 and 1.7 Hz, 1H, H^7), 7.50 (s, 2H, NH$_2$$^{2''}$), 7.46 (dd, J = 8.0 and 0.8 Hz, 1H, H^3), 7.42 (dd, J = 1.6 and 0.4 Hz, 1H, H^6), 7.36 (ddd, J = 7.8, 4.8 and 0.9 Hz, 1H, H^8), 7.24 (dd, J = 8.1 and 1.7 Hz, 1H, H^4), and 5.62 (s, 2H, CH$_2$). ^{13}C-NMR (DMSO-d_6, 101 MHz): δ_C 163.93, 158.70, 156.53, 154.80, 149.42, 149.34, 148.90, 145.37, 144.90, 136.00, 133.22, 128.77, 124.17, 120.94, 115.50, 109.34, 97.96, and 47.91. HPLC-MS (APCI/ESI): purity 99%, t$_R$ = 2.20 min, (m/z) [M+H]$^+$ = 359.1.

5-(4-Amino-1-((5-methylpyridin-3-yl)methyl)-1*H*-pyrazolo[3,4-*d*]pyrimidin-3-yl) benzo[*d*]oxazol-2-amine, 34. White solid (48 mg, 18%); m.p. 285–286°C; R$_f$ (10% MeOH/DCM) 0.32; ^{1}H-NMR (DMSO-d_6, 400 MHz): δ_H 8.38 (d, J = 2.0 Hz, 1H, H^7), 8.33 (d, J = 2.0 Hz, 1H, H^8), 8.30 (s, 1H, H$^{6'}$), 7.53 (broad t, J = 2.4 Hz, 1H, H^9), 7.50 (s, 2H, NH$_2$$^{2''}$), 7.46 (d, J = 8.0 Hz, 1H, H^3), 7.42 (d, J = 1.6 Hz, 1H, H^6), 7.24 (dd, J = 8.4 and 1.6 Hz, 1H, H^4), 5.57 (s, 2H, CH$_2$), and 2.26 (s, 3H, CH$_3$). ^{13}C-NMR (101 MHz, DMSO-d_6): δ_C 163.93, 158.68, 156.50, 154.74, 149.71, 148.89, 146.63, 145.32, 144.87, 136.27, 133.42, 132.67, 128.77, 120.97, 115.50, 109.36, 97.94, 47.82, and 18.22. HPLC-MS (APCI/ESI): purity 97%, t$_R$ = 2.21 min, (m/z) [M+H]$^+$ = 373.1.

5-(4-Amino-1-((5-chloropyridin-3-yl)methyl)-1*H*-pyrazolo[3,4-*d*]pyrimidin-3-yl)benzo[*d*] oxazol-2-amine, 35. White solid (97 mg, 28%); m.p. 274–275°C; R$_f$ (10% MeOH/DCM) 0.34; ^{1}H-NMR (DMSO-d_6, 600 MHz): δ_H 8.52 (d, J = 2.4 Hz, 1H, H^8), 8.48 (d, J = 2.4 Hz, 1H, H^7), 8.26

(s, 1H, $H^{6'}$), 7.82 (t, J = 2.4 Hz, 1H, H^9), 7.48 (s, 2H, $NH_2^{2''}$), 7.42 (d, J = 7.8 Hz, 1H, H^3), 7.38 (d, J = 1.8 Hz, 1H, H^6), 7.20 (dd, J = 7.8 and 1.8 Hz, 1H, H^4), and 5.60 (s, 2H, CH_2). ^{13}C-NMR (151 MHz, DMSO-d_6): δ_C 163.89, 158.69, 156.59, 154.83, 148.88, 147.93, 147.75, 145.59, 144.86, 135.68, 134.93, 131.38, 128.63, 120.92, 115.47, 109.34, 97.96, and 47.19. HPLC-MS (APCI/ESI): purity 99%, t_R = 2.34 min, (*m/z*) $[M+H]^+$ = 393.1.

5-(4-Amino-1-((5-fluoropyridin-3-yl)methyl)-1*H*-pyrazolo[3,4-*d*]pyrimidin-3-yl)benzo[*d*] oxazol-2-amine, 36. White solid (74 mg, 24%); m.p. 285–286°C; R_f (10% MeOH/ DCM) 0.36; ^{1}H-NMR (DMSO-d_6, 600 MHz): δ_H 8.47 (broad d, J = 2.4 Hz, 1H, H^8), 8.40 (broad t, J = 1.8 Hz, 1H, H^7), 8.25 (s, 1H, $H^{6'}$), 7.60 (dt, J = 9.0 and 2.4 Hz, 1H, H^9), 7.48 (s, 2H, $NH_2^{2''}$), 7.42 (d, J = 7.8 Hz, 1H, H^3), 7.38 (d, J = 1.8 Hz, 1H, H^6), 7.20 (dd, J = 8.4 and 1.8 Hz, 1H, H^4), and 5.62 (s, 2H, CH_2). ^{13}C-NMR(151 MHz, DMSO-d_6): δ_C 163.89, 159.31 (d, J = 253.7 Hz, $^1J_{C\text{-}F}$, 1C), 158.69, 156.57, 154.83, 148.88, 145.61 (overlapping d, J = 4.5 Hz, $^4J_{C\text{-}F}$, 1C), 145.58, 144.85, 137.52 (d, J = 22.7 Hz, $^2J_{C\text{-}F}$, 1C), 135.21 (d, J = 3.0 Hz, $^3J_{C\text{-}F}$, 1C), 128.64, 122.84 (d, J = 19.6 Hz, $^2J_{C\text{-}F}$, 1C), 120.94, 115.48, 109.33, 97.96, and 47.18. HPLC-MS (APCI/ESI): purity 98%, t_R = 2.26 min, (*m/z*) $[M+H]^+$ = 377.1.

5-(4-Amino-1-(cyclohexylmethyl)-1*H*-pyrazolo[3,4-*d*]pyrimidin-3-yl)benzo[*d*]oxazol-2-amine, 37. Off-white solid (97 mg, 24%); m.p. 285–286°C; R_f (10% MeOH/ DCM) 0.46; ^{1}H-NMR (DMSO-d_6, 400 MHz): δ_H 8.19 (s, 1H, $H^{6'}$), 7.47 (s, 2H, $NH_2^{2''}$), 7.42 (d, J = 7.8 Hz, 1H, H^3), 7.37 (d, J = 1.2 Hz, 1H, H^3), 7.20 (dd, J = 8.4 and 1.8 Hz, 1H, H^4), 4.14 (d, J = 6.6 Hz, 2H, CH_2), 1.92 (ttt, J = 10.8, 7.1 and 3.4 Hz, 1H, H^7), 1.63–1.60 (m, 2H, H^{9a}), 1.55–1.49 (m, 3H, $H^{8e, 10e}$), 1.16–1.08 (m, 3H, $H^{10a, 9e}$) and 1.00–0.94 (m, 2H, H^{8a}). ^{13}C-NMR (DMSO-d_6, 101 MHz): δ_C 163.87, 158.59, 156.06, 154.83, 148.74, 144.84, 144.32, 129.06, 120.88, 115.44, 109.28, 97.54, 52.52, 38.19, 30.58 (2C), 26.30, 25.58 (2C). HPLC-MS (APCI/ESI): purity >99%, t_R = 2.51 min, (*m/z*) $[M+H]^+$ = 364.1.

5-(4-amino-1-((4,4-difluorocyclohexyl)methyl)-1*H*-pyrazolo[3,4-*d*]pyrimidin-3-yl) benzo[*d*]oxazol-2-amine, 38. Off-white solid (186 mg, 45%); m.p. 288–289°C; R_f (10% MeOH/DCM) 0.37; ^{1}H-NMR (DMSO-d_6, 600 MHz): δ_H 8.21 (s, 1H, $H^{6'}$), 7.48 (s, 2H, $NH_2^{2''}$), 7.43 (d, J = 7.8 Hz, 1H, H^3), 7.38 (d, J = 1.8 Hz, 1H, H^6), 7.21 (dd, J = 8.4 and 1.8 Hz, 1H, H^4),

4.23 (d, J = 7.2 Hz, 1H, CH_2), 2.08 (ddd, J = 10.3, 7.8, and 3.6 Hz, 1H, H^7), 1.98–1.93 (m, 2H, H^{9e}), 1.78–1.69 (m, 2H, H^{9a}), 1.63–1.60 (m, 2H, H^{8e}), 1.26 (qd, J = 13.2 and 3.6 Hz, 2H, H^{8a}). ^{13}C-NMR (DMSO-d_6, 151 MHz): δ_C 163.88, 158.62, 156.17, 154.85, 148.78, 144.84, 144.60, 128.96, 124.63 (t, J = 241.6 Hz, $^1J_{C\text{-}F}$, 1C), 120.89, 115.46, 109.30, 97.57, 51.01, 35.93, 32.63 (t, J = 24.2 Hz, $^2J_{C\text{-}F}$, 2C), and 26.67 (d, J = 9.1 Hz, $^3J_{C\text{-}F}$, 2C). HPLC-MS (APCI/ESI): purity 98%, t_R = 2.41 min, (m/z) $[M+H]^+$ = 400.1.

Characterization Data for Amides and Sulphonamides

4-((4-Amino-3-(2-aminobenzo[d]oxazol-5-yl)-1H-pyrazolo[3,4-d]pyrimidin-1-yl)methyl)benzamide, 46a. Pale brown solid (72.7 mg, 36% yield; m.p. 279-281 °C; R_f=0.31; 20%MeOH/DCM). ^{1}H NMR (400 MHz, DMSO-d_6) δ = 8.29 (s, 1H, H^1), 7.89 (s, 1H, H^9), 7.82 (d, J = 8.0 Hz, 2H, H^8), 7.51 (s, 2H, H^5), 7.47 (d, J = 8.1 Hz, 1H, H^3), 7.42 (d, J = 1.6 Hz, 1H, H^4), 7.35 (d, J = 8.4 Hz, 2H, H^7), 7.29 (s, 1H, H^9), 7.25 (dd, J = 8.2, 1.7 Hz 1H, H^2), and 5.62 ppm (s, 2H, H^6). ^{13}C NMR (101 MHz, DMSO-d_6) δ = 168.06, 163.93, 158.69, 156.49, 154.83, 148.88, 145.24, 144.90, 140.75, 134.12, 128.83, 128.23 (2C), 127.81 (2C), 120.94, 115.49, 109.34, 97.90, and 49.95 ppm. HPLC-MS (APCI/ESI): purity = 96%, t_R = 2.15 min, m/z $[M+H]^+$ = 401.1.

3-((4-amino-3-(2-aminobenzo[d]oxazol-5-yl)-1H-pyrazolo[3,4-d]pyrimidin-1-yl)methyl)benzamide, 46b. Pale white solid (23.2 mg, 12% yield; m.p. 295-297 °C; R_f=0.36; 20%MeOH/DCM). ^{1}H NMR (600 MHz, DMSO-d_6) δ 8.25 (s, 1H, H^1), 7.92 (s, 1H, H^7), 7.79 (t, J = 1.9 Hz, 1H, H^3), 7.73 (dt, J = 7.1 and 1.8 Hz, 1H, H^4), 7.47 (s, 2H, H^{11}), 7.42 (d, J = 8.1 Hz, 1H, H^{10}), 7.40 – 7.35 (m, 3H, H^5, H^6, H^8), 7.29 (s, 1H, H^7), 7.20 (dd, J = 8.1 and 1.8 Hz, 1H, H^9), 5.56 (s, 2H, H^2).^{13}C NMR (151 MHz, DMSO-d_6) δ 168.05, 163.87, 158.65, 156.45, 154.74, 148.82, 145.16, 144.85, 137.79, 135.06, 130.89, 128.86, 128.80, 127.41, 126.95, 120.91, 115.45, 109.32, 97.84 and 50.03 ppm. HPLC-MS (APCI/ESI): purity = 96%, t_R = 2.15 min, m/z $[M+H]^+$ = 401.1.

2-((4-amino-3-(2-aminobenzo[d]oxazol-5-yl)-1H-pyrazolo[3,4-d]pyrimidin-1-yl)methyl)benzamide, 46c. Pale white solid (32 mg, 16% yield; m.p. 296-298 °C; R_f=0.4; 20%MeOH/DCM). ^{1}H NMR (600 MHz, DMSO-d_6) δ 8.21 (s, 1H, H^1), 8.02 (s, 1H, H^7), 7.53 –

7.49 (m, 2H, H^3 and H^7), 7.48 (s, 2H, H^{11}), 7.43 (d, J = 8.1 Hz, 1H, H^{10}), 7.39 (d, J = 1.7 Hz, 1H, H^8), 7.31 – 7.27 (m, 2H, H^4 and H^5), 7.22 (dd, J = 8.1 and 1.7 Hz, 1H, H^9), 6.77 – 6.73 (m, 1H, H^6), 5.75 (s, 2H, H^2). ^{13}C NMR (151 MHz, DMSO-d_6) δ 170.57, 163.88, 158.68, 156.42, 155.02, 148.83, 145.19, 144.86, 135.94, 135.65, 130.44, 128.82, 128.16, 127.65, 127.54, 120.94, 115.50, 109.31, 97.82 and 47.86 ppm. HPLC-MS (APCI/ESI): purity = 99%, t_R = 2.21 min, *m/z* $[M+H]^+$ = 401

4-((4-amino-3-(2-aminobenzo[d]oxazol-5-yl)-1H-pyrazolo[3,4-d]pyrimidin-1-yl)methyl)benzenesulphonamide, 46d. Pale white solid (12.1 mg, 4% yield; m.p. 235-237 °C; R_f=0.4; 20%MeOH/DCM). ^{1}H NMR (600 MHz, DMSO-d_6) δ 8.24 (s, 1H, H^1), 7.74 (d, J = 8.5 Hz, 2H, H^4), 7.48 (s, 2H, H^9), 7.43 – 7.41 (m, 3H, H^3, H^8), 7.38 (d, J = 1.8 Hz, 1H, H^6), 7.25 (s, 2H, H^5), 7.20 (dd, J = 8.1 and 1.8 Hz, 1H, H^7), 5.60 (s, 2H, H^2). ^{13}C NMR (151 MHz, DMSO-d_6) δ 163.88, 158.67, 156.51, 154.81, 148.85, 145.35, 144.86, 143.75, 141.46, 128.74, 128.46 (2C), 126.44 (2C), 120.92, 115.48, 109.32, 97.88 and 49.75 ppm.

4-((4-amino-3-(2-aminobenzo[d]oxazol-5-yl)-1H-pyrazolo[3,4-d]pyrimidin-1-yl)methyl)-N-methylbenzenesulphonamide, 46e. Pale white solid (52 mg, 26% yield; m.p. 273-275 °C; R_f=0.54; 20%MeOH/DCM). ^{1}H NMR (600 MHz, DMSO-d_6) δ 8.24 (s, 1H, H^1), 7.70 (d, J = 8.1 Hz, 2H, H^4), 7.48 (s, 2H, H^{10}), 7.45 (d, J = 8.6 Hz, 2H, H^3), 7.42 (d, J = 7.9 Hz, 1H, H^9), 7.39 (d, J = 1.7, 1H, H^7), 7.36 (q, J = 5.0 Hz, 1H, H^5), 7.21 (dd, J = 8.1 and 1.7 Hz, 1H, H^8), 5.62 (s, 2H, H^2), 2.35 (d, J = 5.0 Hz, 3H, H^6). ^{13}C NMR (151 MHz, DMSO-d_6) δ 163.89, 158.69, 156.54, 154.86, 148.86, 145.41, 144.87, 142.15, 138.99, 128.72, 128.64 (2C), 127.47 (2C), 120.93, 115.49, 109.33, 97.86 and 49.68, 29.09 ppm. HPLC-MS (APCI/ESI): purity = 99%, t_R = 2.24 min, *m/z* $[M+H]^+$ = 451

4-((4-amino-3-(2-aminobenzo[d]oxazol-5-yl)-1H-pyrazolo[3,4-d]pyrimidin-1-yl)methyl)-N,N-dimethylbenzenesulphonamide, 46f. Pale white solid (95.2 mg, 47% yield; m.p. 290-291 °C; R_f=0.58; 20%MeOH/DCM). ^{1}H NMR (600 MHz, DMSO-d_6) δ 8.25 (s, 1H, H^1), 7.69 (d, J = 8.5 Hz, 2H, H^4), 7.48 – 7.47 (m, 4H, H^3, H^9), 7.43 (d, J = 8.1 Hz, 1H, H^8), 7.39 (d, J = 1.7, 1H, H^6), 7.21 (dd, J = 8.1 and 1.7 Hz, 1H, H^7), 5.65 (s, 2H, H^2), 2.55 (s, 6H, H^5). ^{13}C NMR (151 MHz, DMSO-d_6) δ 163.89, 158.70, 156.57, 154.92, 148.87, 145.47, 144.88, 142.83, 134.56, 128.70,

128.67 (2C), 128.36 (2C), 120.92, 115.48, 109.34, 97.84, 49.60 and 37.95 (2C) ppm. HPLC-MS (APCI/ESI): purity = 99%, t_R = 2.27 min, *m/z* $[M+H]^+$ = 465

3-((4-amino-3-(2-aminobenzo[d]oxazol-5-yl)-1H-pyrazolo[3,4-d]pyrimidin-1-yl)methyl)-N,N-dimethylbenzenesulfonamide, 46g. Pale white solid (45 mg, 11% yield; m.p. 242-245 °C; R_f=0.8; 20%MeOH/DCM). ^{1}H NMR (600 MHz, DMSO-d_6) δ 8.24 (s, 1H, H^1), 7.64 (broad t, *J* = 1.3 Hz, 1H, H^3), 7.63 – 7.60 (m, 1H, H^4), 7.58 – 7.57 (m, 2H, H^5 and H^6), 7.48 (s, 2H, H^{11}), 7.43 (d, *J* = 8.2 Hz, 1H, H^{10}), 7.36 (d, *J* = 1.2 Hz, 1H, H^8), 7.19 (dd, *J* = 1.7, 8.1 Hz, 1H, H^9), 5.66 (s, 2H, H^2), 2.50 (s, 6H, H^7). ^{13}C NMR (151 MHz, DMSO-d_6) δ 163.90, 158.68, 156.53, 154.90, 148.86, 145.45, 144.87, 139.10, 135.40, 132.58, 130.21, 128.69, 127.06, 127.00, 120.84, 115.40, 109.35, 97.86, 49.65 and 37.91 (2C) ppm. HPLC-MS (APCI/ESI): purity = 99%, t_R = 2.30 min, *m/z* $[M+H]^+$ = 465.1

3-((4-amino-3-(2-aminobenzo[d]oxazol-5-yl)-1H-pyrazolo[3,4-d]pyrimidin-1-yl)methyl)-N-methylbenzenesulfonamide, 46h.

Pale white solid (25.2 mg, 5% yield; m.p. 261-263 °C; R_f=0.7; 20%MeOH/DCM). ^{1}H NMR (600 MHz, DMSO-d_6) δ 8.25 (s, 1H, H^1), 7.68 (broad t, *J* = 1.7 Hz, 1H, H^3), 7.65 (dt, *J* = 7.2, 1.9 Hz, 1H, H^4), 7.55 (overlapping t, *J* = 7.7 Hz, 1H, H^5), 7.53 – 7.52 (m, 1H, H^6), 7.48 (s, 2H, H^{12}), 7.43 (d, *J* = 8.1 Hz, 1H, H^{11}), 7.41 (overlapping q, *J* = 5.2 Hz, 1H, H^7), 7.37 (d, *J* = 1.7 Hz, 1H, H^9), 7.20 (dd, *J* = 1.8, 8.1 Hz, 1H, H^{10}), 5.63 (s, 2H, H^2), 2.34 (d, *J* = 5.0 Hz, 3H, H^8). ^{13}C NMR (151 MHz, DMSO-d_6) δ 163.89, 158.68, 156.53, 154.85, 148.86, 145.41, 144.87, 140.13, 138.92, 132.03, 130.07, 128.72, 126.22, 126.08, 120.89, 115.45, 109.34, 97.86, 49.69 and 29.06 ppm. HPLC-MS (APCI/ESI): purity = 99%, t_R = 2.16 min, *m/z* $[M+H]^+$ = 451

2. ^{1}H, ^{13}C NMR and LC MS Spectra of Frontrunner Compounds and Key Intermediates

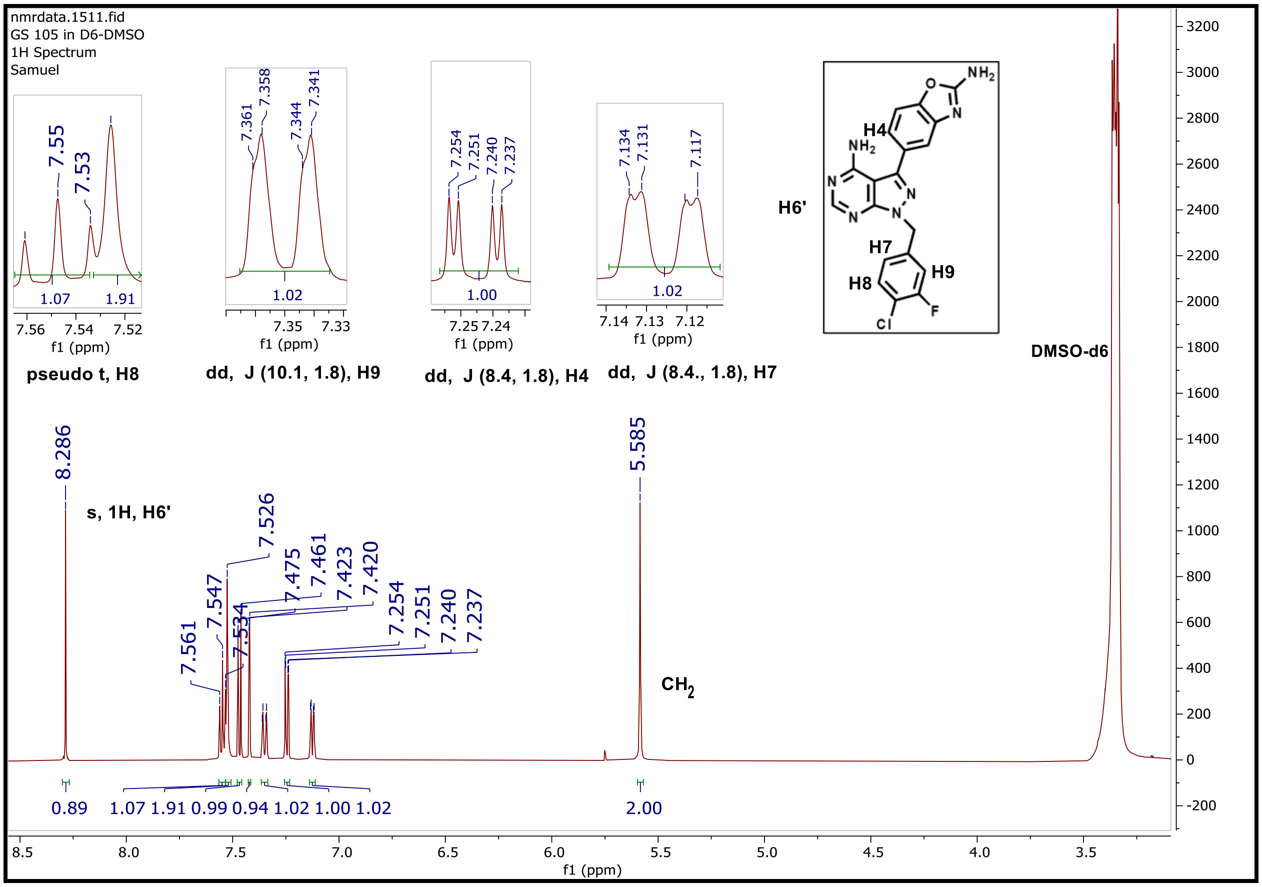

Figure S1: 600 MHz ^{1}H NMR spectrum of the frontrunner compound **19** in DMSO-d_6

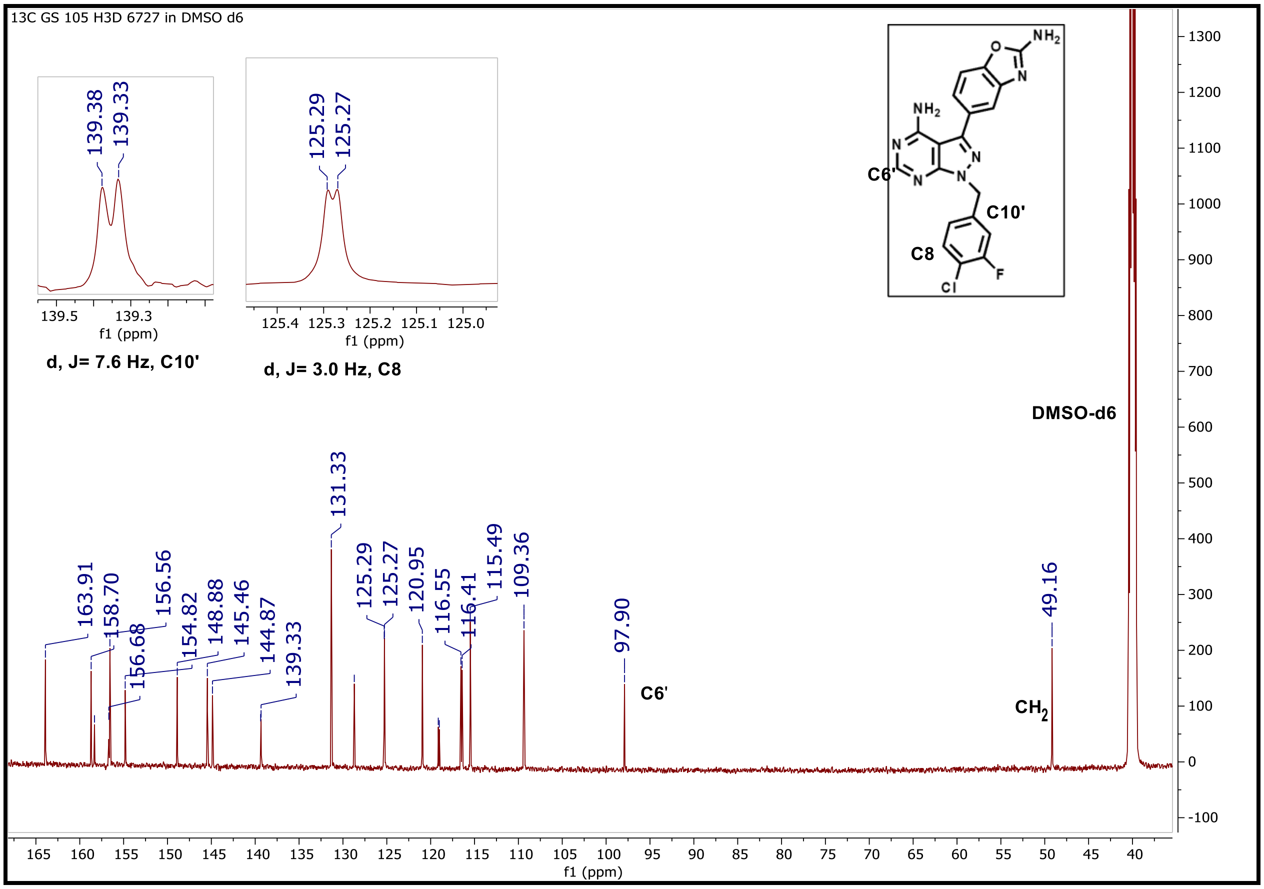

Figure S2: 151 MHz ^{13}C NMR spectrum of the frontrunner compound **19** in DMSO-d_6

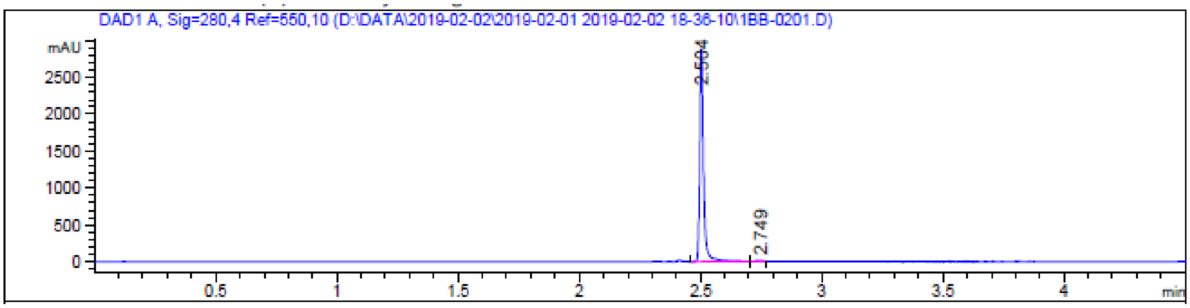

Signal 1: DAD1 A, Sig=280,4 Ref=550,10

Peak #	RetTime [min]	Type	Width [min]	Area [mAU*s]	Height [mAU]	Area %
1	2.504	BV	0.0169	3278.30420	2858.78491	99.3292
2	2.749	VV	0.0349	22.14057	8.08786	0.6708

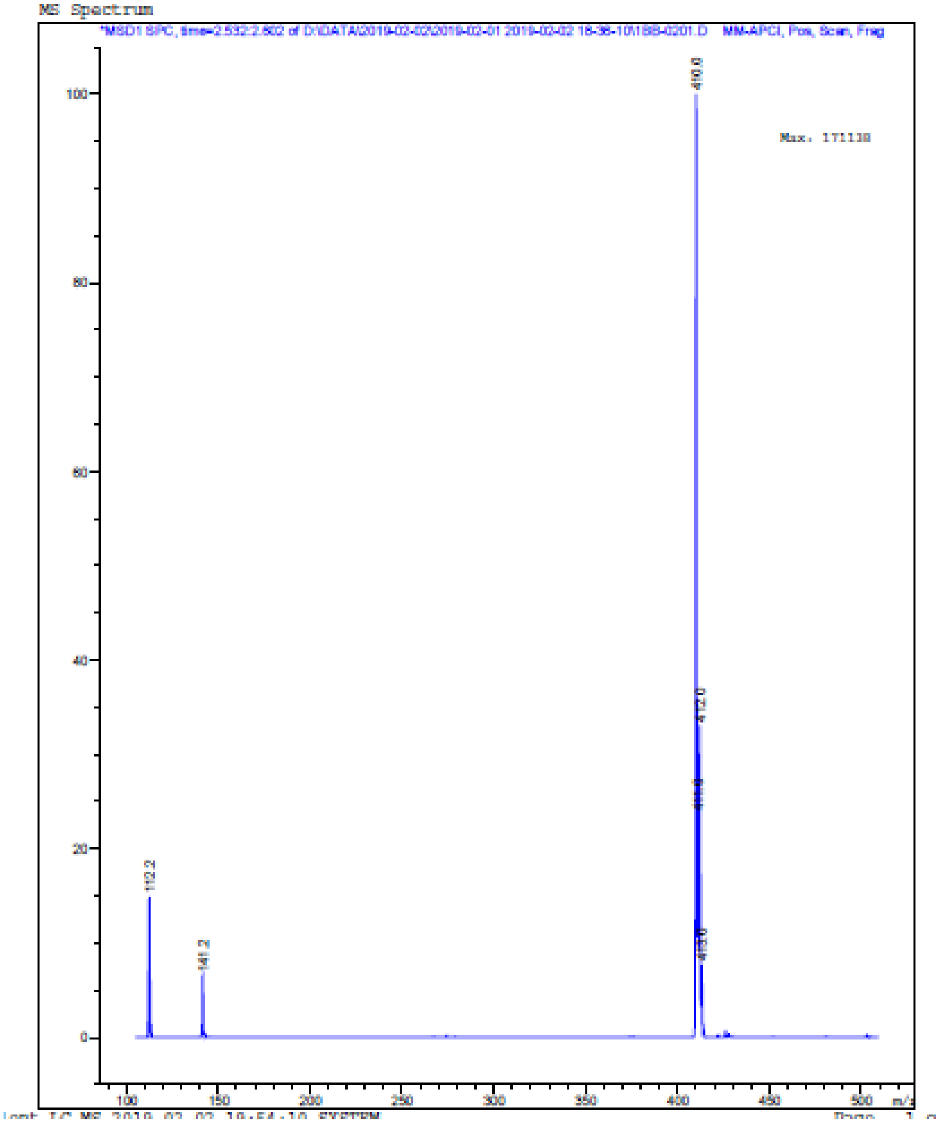

Figure S3: HPLC chromatogram and APCI$^+$ mass spectrum of compound **19**

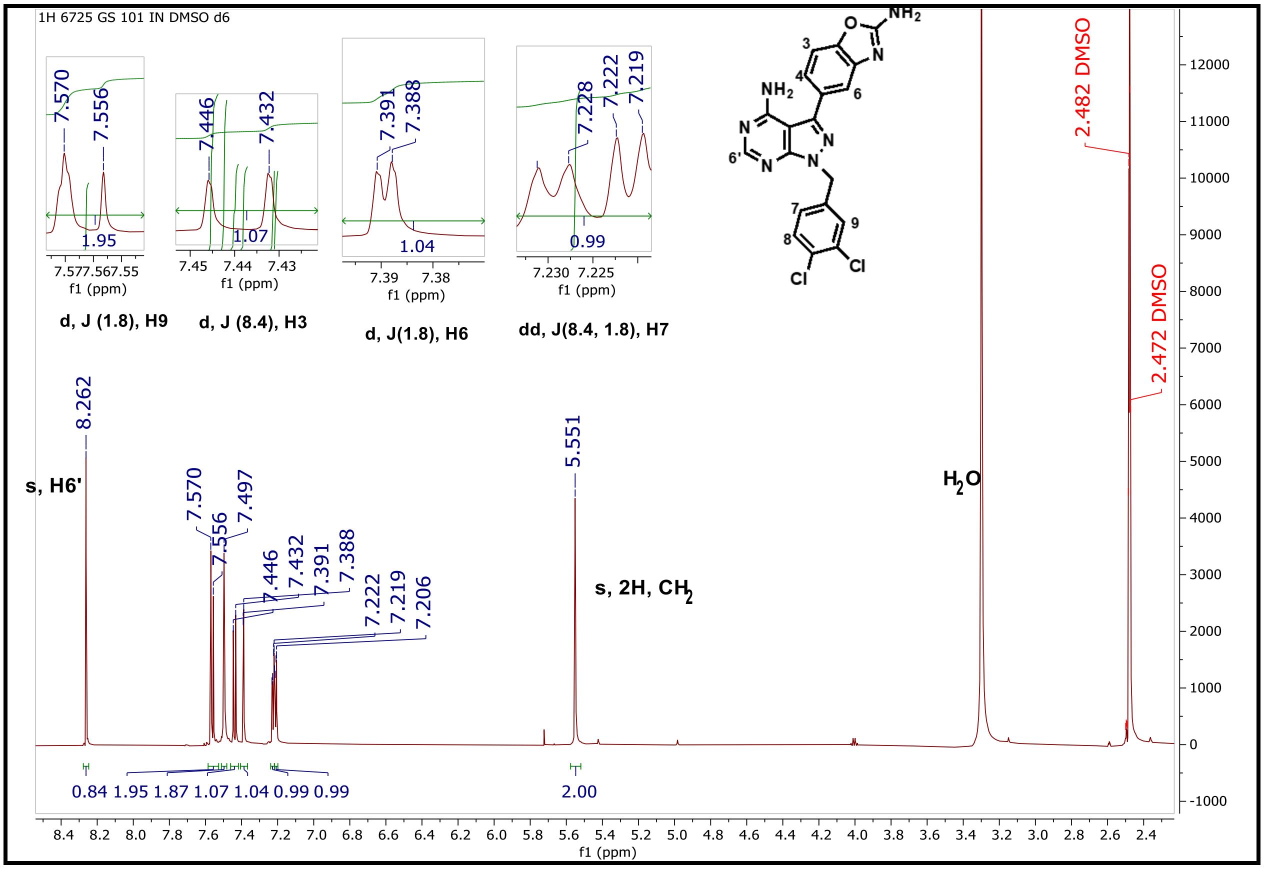

Figure S4: 600 MHz ^{1}H NMR spectrum of compound **20** in DMSO-d_6

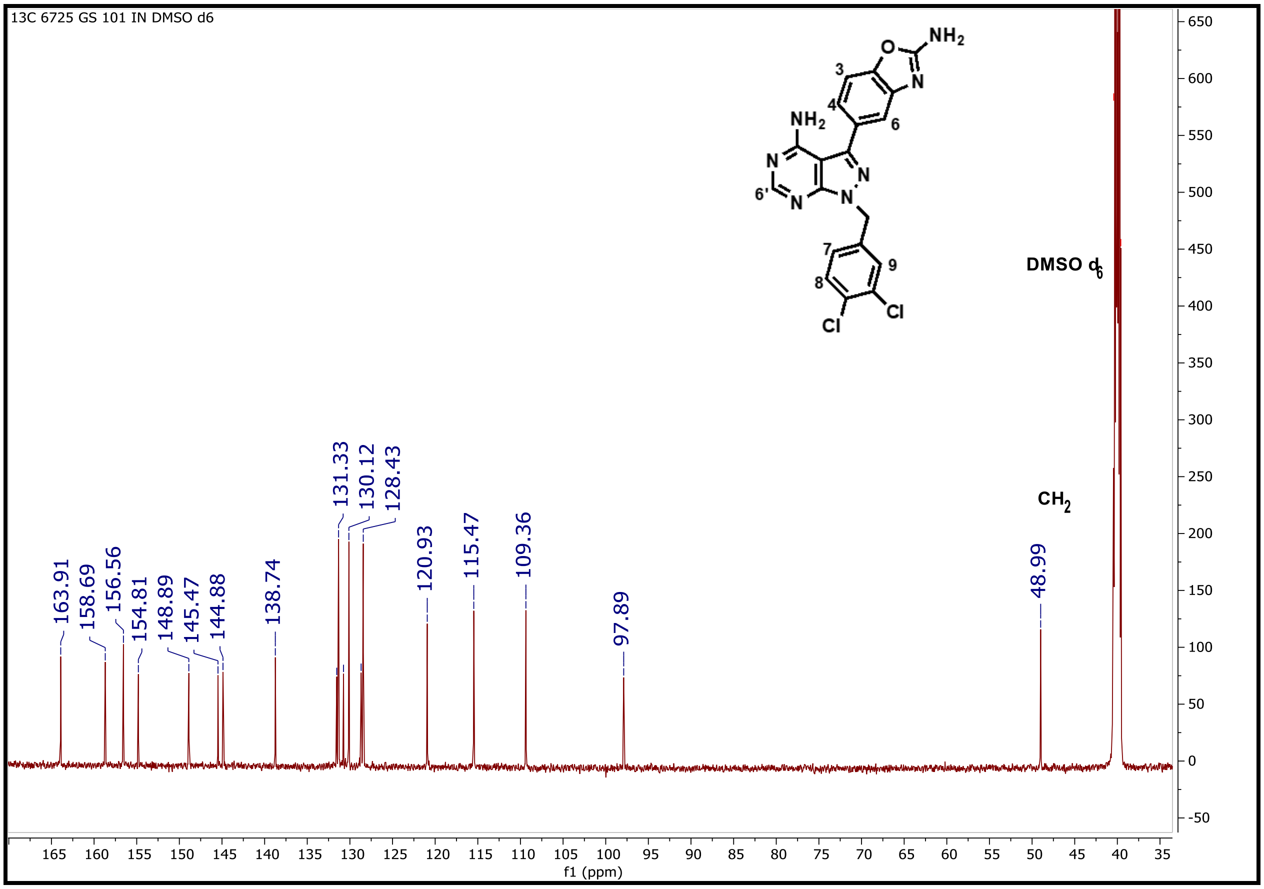

Figure S5: 151 MHz ^{13}C NMR spectrum of compound **20** in DMSO-d_6

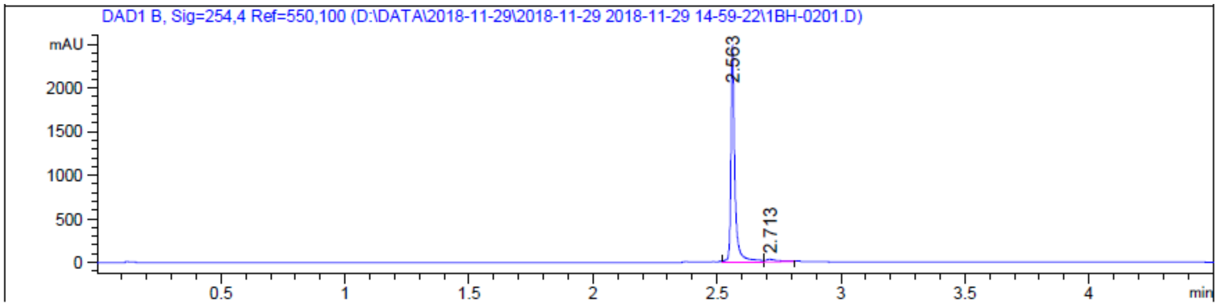

Signal 2: DAD1 B, Sig=254,4 Ref=550,100

Peak #	RetTime [min]	Type	Width [min]	Area [mAU*s]	Height [mAU]	Area %
1	2.563	VV	0.0162	2800.99365	2469.06006	96.3718
2	2.713	VV	0.0454	105.45214	30.80031	3.6282

MS Spectrum

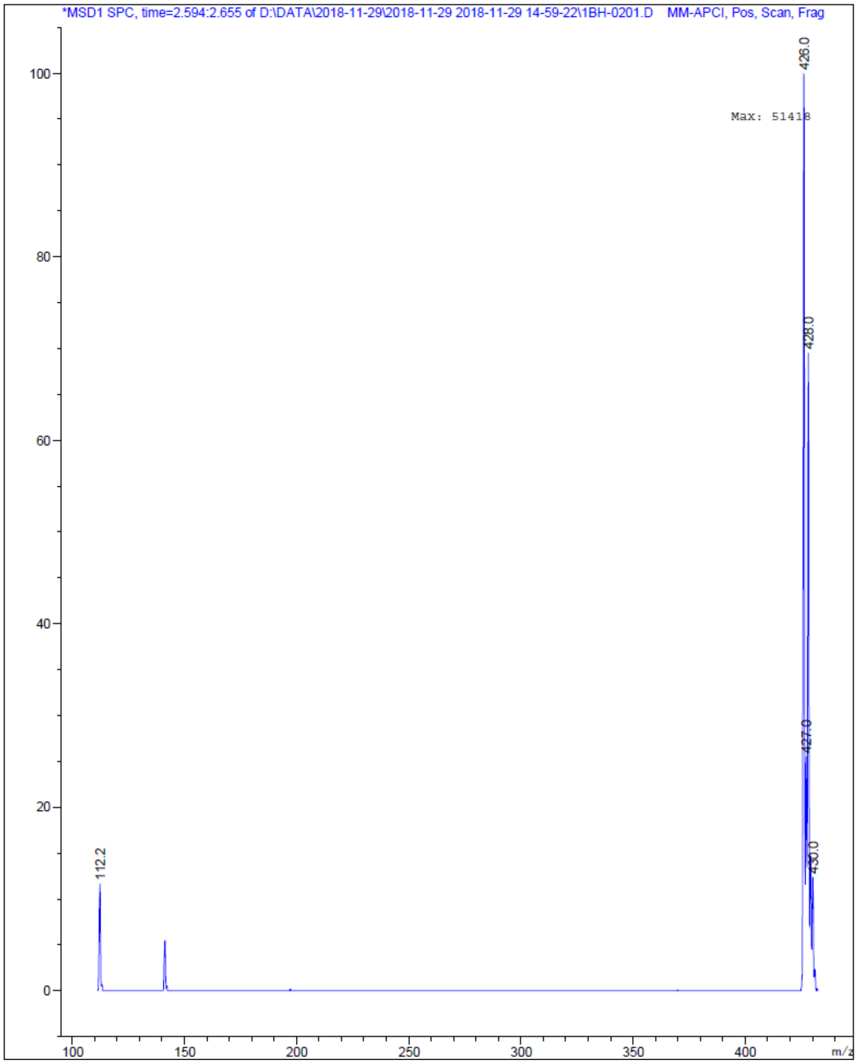

Figure S6: HPLC chromatogram and APCI^{+} mass spectrum of compound **20**

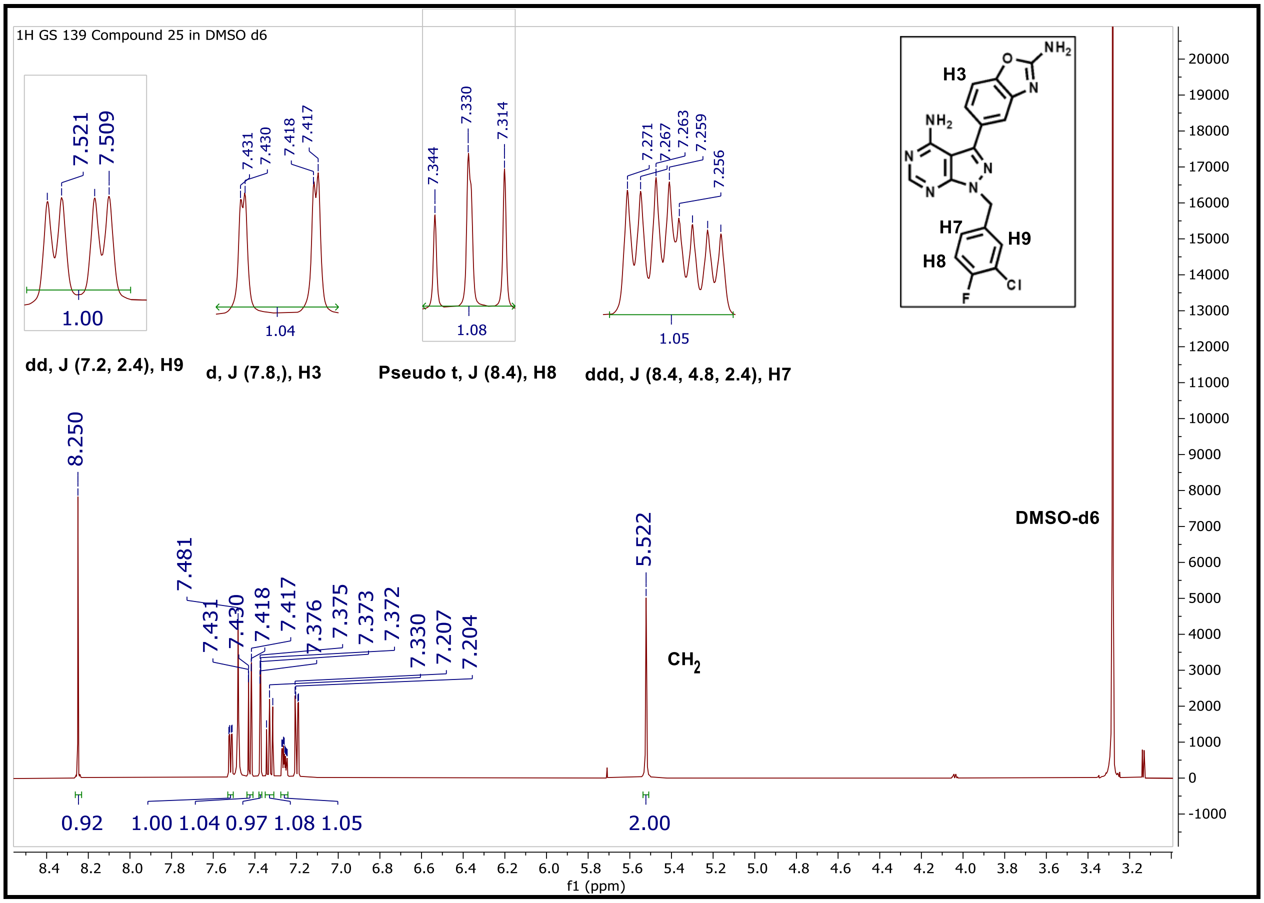

Figure S7: 600 MHz ^{1}H NMR spectrum of compound **25** in DMSO-d_6

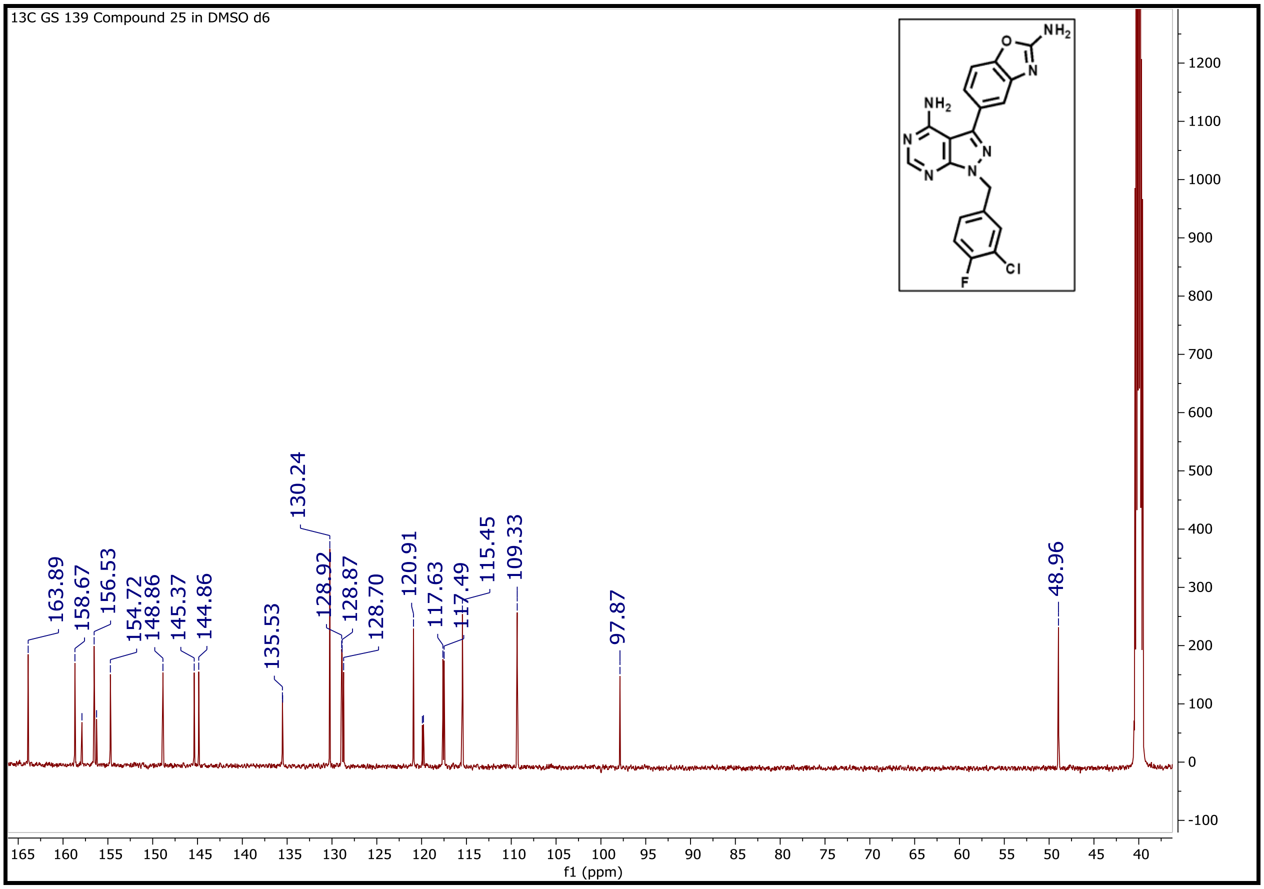

Figure S8: 151 MHz ^{13}C NMR spectrum of compound **25** in DMSO-d_6

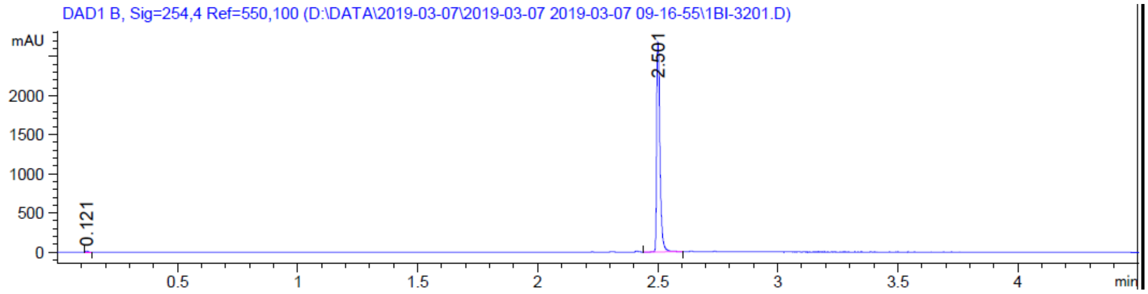

Signal 2: DAD1 B, Sig=254,4 Ref=550,100

Peak #	RetTime [min]	Type	Width [min]	Area [mAU*s]	Height [mAU]	Area %
1	0.121	BB	0.0119	11.40884	14.90175	0.4433
2	2.501	BV	0.0152	2562.30371	2672.99854	99.5567

MS Spectrum

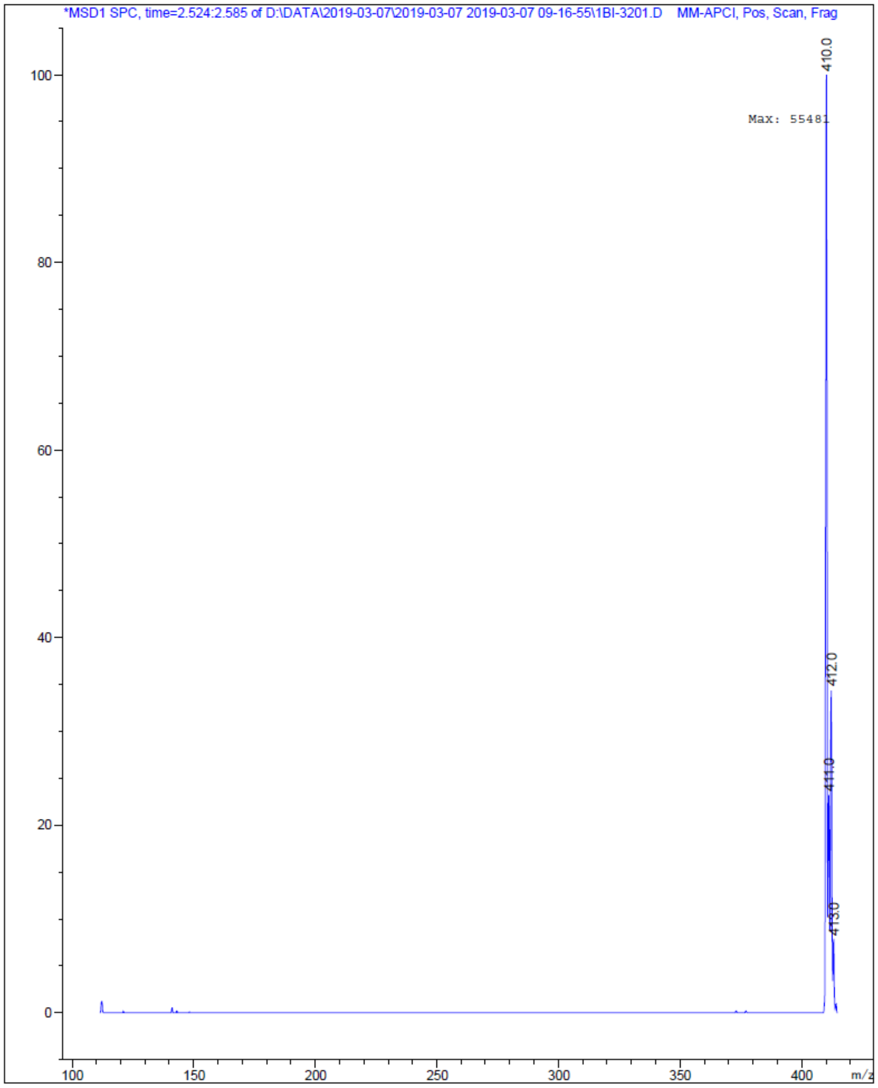

Figure S9: HPLC chromatogram and APCI$^+$ mass spectrum of compound **25**

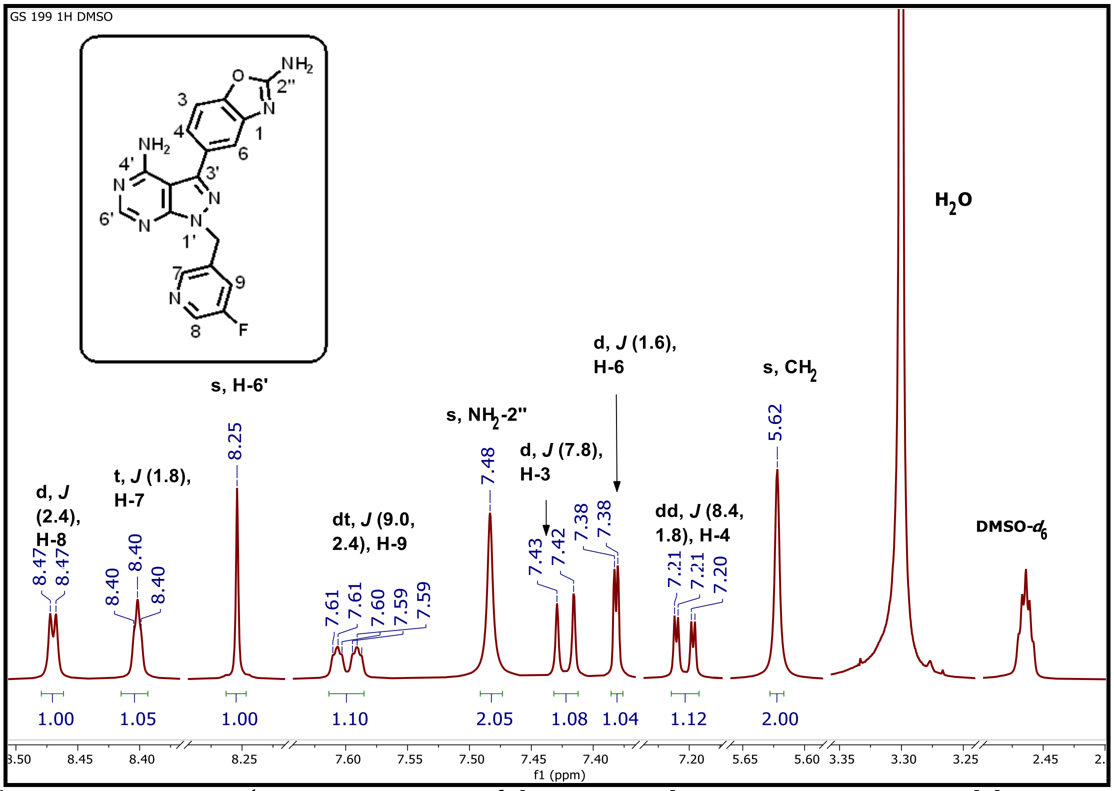

Figure S10: 600 MHz ^{1}H-NMR spectrum of the compound **36**, a representative pyridyl compound

^{1}H NMR Spectra of Key Intermediates

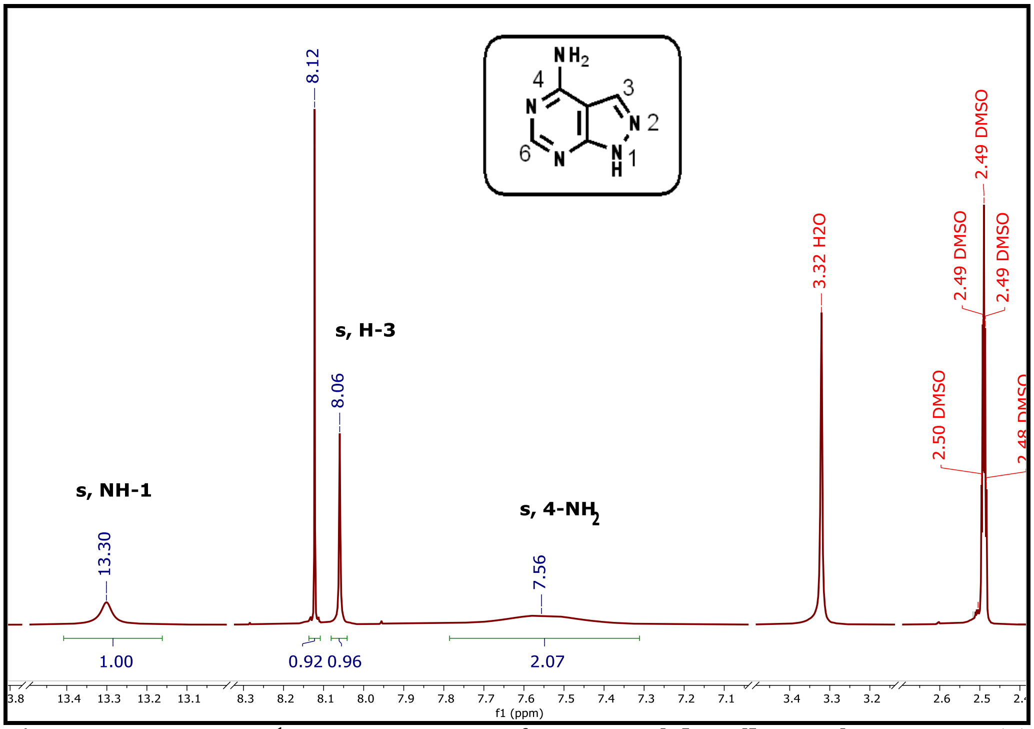

Figure S11: 400 MHz ^{1}H NMR spectrum of 1*H*-pyrazolo[3,4-d]pyrimidin-4-amine (**2**)

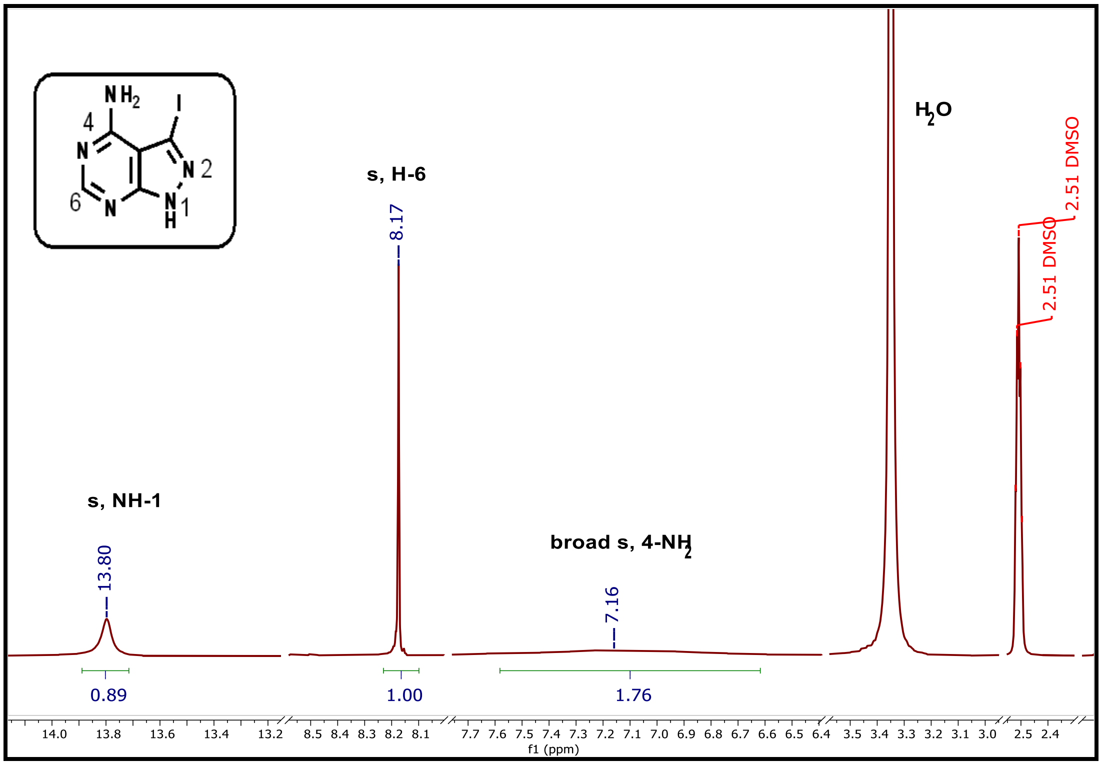

Figure S12: ^{1}H-NMR spectrum of 3-iodo-*1H*-pyrazolo[3,4-d]pyrimidin-4-amine, **3** in DMSO-d_6

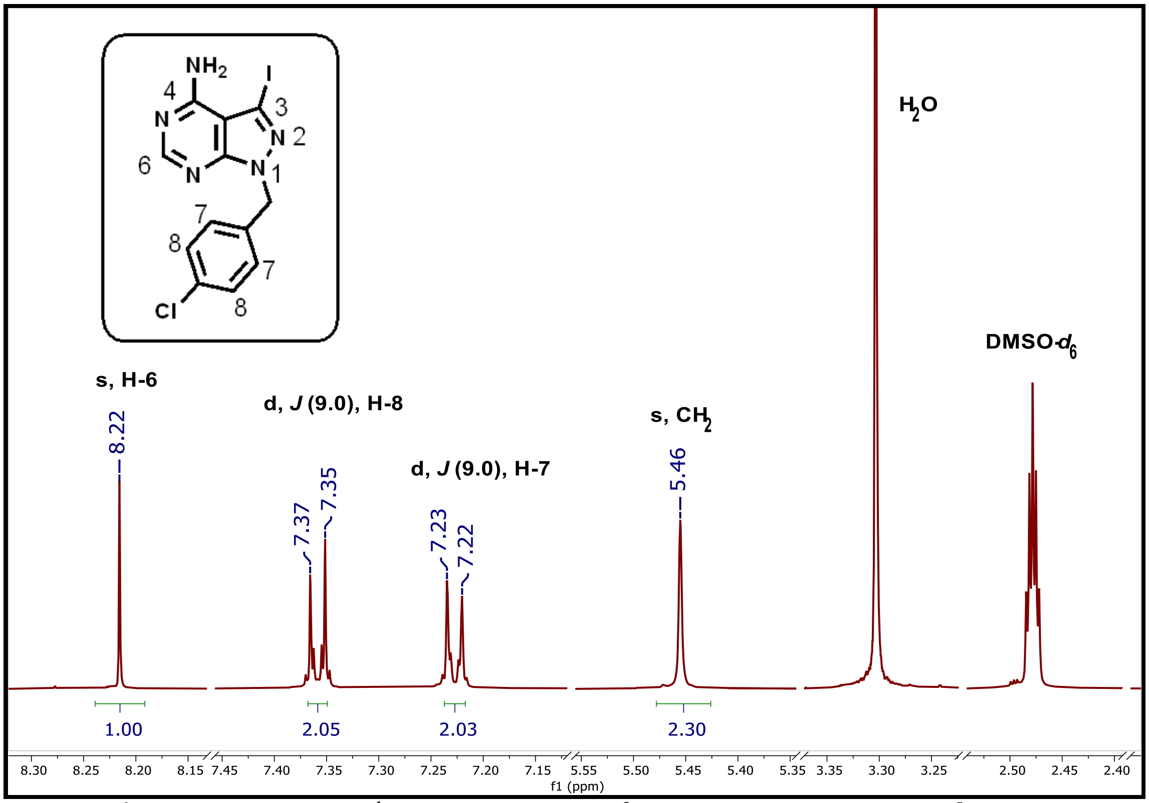

Figure S13: 400 MHz ^{1}H-NMR spectrum of representative S_N1 intermediate **7a**

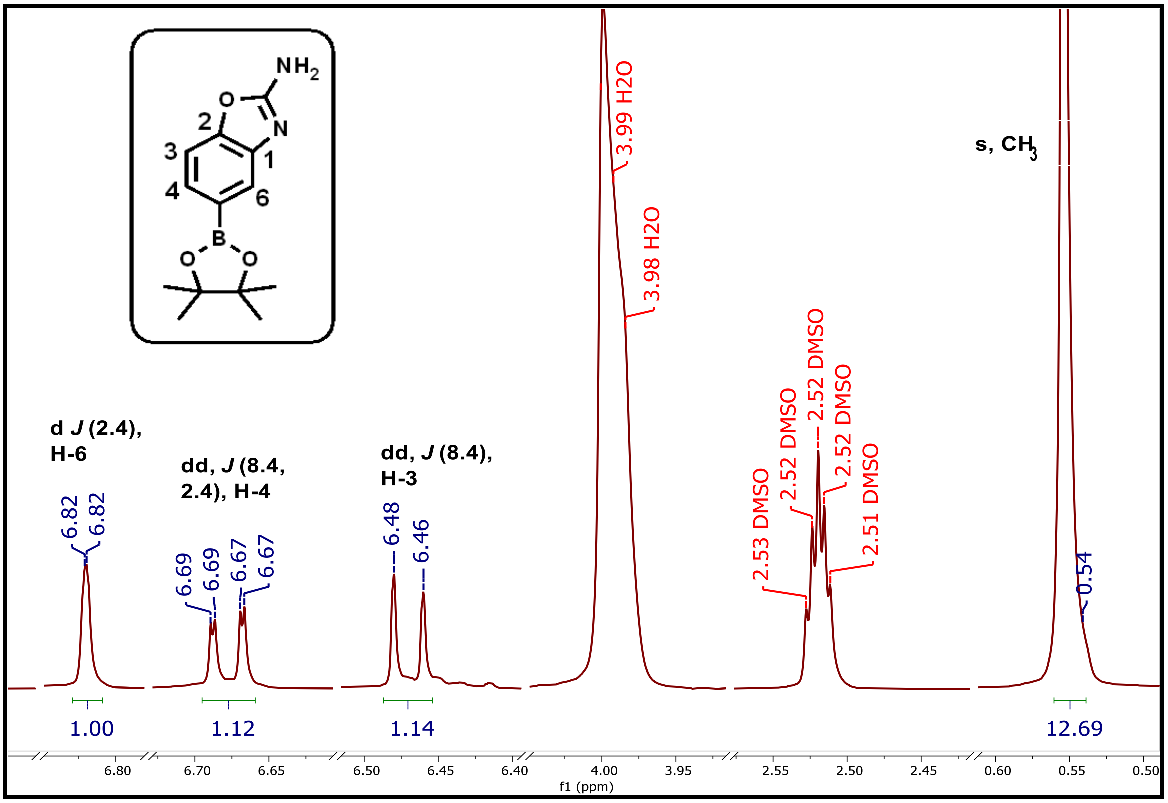

Figure S14: 400 MHz ^{1}H-NMR spectrum of the benzoxazole intermediate, **5** in DMSO-d_6

3. *In silico* Molecular Docking

The *Pf*PI4Kβ homology model[1], the *Pv*PKG crystal structure (PDB ID 5F0A), and the human mTOR crystal structure (PDB ID 4JT5) were all prepared for docking using the Maestro protein preparation tool (Schrödinger Release 2021-3: Maestro, Schrödinger, LLC, New York, NY, 2021).[1,2] Once prepared, a docking grid was created around the binding site using the GLIDE docking grid generation tool with a hydrogen bonding constraint set on the backbone amide of the critical hinge valine (*Pf*PI4Kβ, V1357; *Pv*PKG, V614; HumTOR, V2240). The structures of sapanisertib and other analogues were drawn using the Maestro 2D sketcher and prepared using the LigPrep tool at pH 7.0. Inhibitors were docked using GLIDE at standard precision with the hydrogen bond constraint implemented. The output docked poses were consistent with the binding mode observed in kinase co-crystals for the related compound TORkinib. Full residues within a 5.0-Å radius of the ligand were minimized using PRIME minimization in the function and the OPLS4 force field.

4. Biological and Solubility Evaluation

In vitro Asexual Blood Stage Antiplasmodium LDH Assay

Compounds were screened against the CQ-sensitive *Pf*NF54 and multi-drug resistant *Pf*K1 strains using the parasite Lactate Dehydrogenase (pLDH) assay. Parasites were cultured and maintained according to the approach by Trager and Jensen with slight variations.[1] Stock solutions of test compounds were prepared at 10 mM in 100% HPLC-grade DMSO (Sigma Aldrich). Subsequent dilutions of the stock solutions were prepared in medium to give a 10-point dose response curve with a highest starting concentration of 6 µM. Chloroquine and artesunate were employed as positive controls. Non-linear dose-response curves generated in GraphPad Prism v.6.01 software were used to evaluate the IC_{50} values.

In vitro Asexual Blood Stage Antiplasmodium [^{3}H]-Hypoxanthine Incorporation Assay

Compounds were screened against CQ-sensitive *Pf*NF54 and multi-drug resistant *Pf*K1 strains using the modified [^{3}H]-hypoxanthine incorporation *in vitro* assay.[3]

In vitro Luciferase Reporter Gametocytocidal Assay

Two transgenic parasite lines (NF54-*Pf*S16-GFP-Luc (early stage) and NF54-Mal8p1.16-GFP-Luc (late stage)) were used in the luciferase assays, which facilitated stage-specific determination of gametocytocidal activity. Gametocytes were produced according to Reader *et al.*[4,5] On days 5 and 10, which respectively represent > 90% of immature (I – III) and >90% late-stage (IV and V) gametocytes, drug assays were set up. In both cases, 2 - 3% gametocytemia and 1.5% hematocrit culture were used with a 48 h drug pressure under hypoxic conditions (90% N_2, 5% O_2 and 5% CO_2) at 37ºC. To 20 µL parasite lysates, 50 µL of luciferin substrate (Promega Luciferase Assay System) was added at room temperature (22ºC). Luciferase activity was determined by detecting resulting bioluminescence at an integration constant of 10 seconds using a GloMax®-Multi$^+$ Detection System with Instinct® Software. Methylene blue and dihydroartemisinin were used as controls.

In vitro P. vivax PI4Kβ Inhibition Assay

Full-length *Pv*PI4Kβ (PVX_098050) recombinant protein expressed in a baculovirus-insect cell expression system and purified as previously described was used in the assay.[6,7]. Briefly, a 10-point 3-fold serial dilution of each inhibitor was carried out in DMSO and inhibitors were

subsequently diluted into assay buffer (25 mM HEPES pH 7.4, 100 mM NaCl, 3 mM $MgCl_2$, 1 mM DTT, 0.025 mg/ml BSA, 0.2% (v/v) Triton-X-100). The final 20 µL kinase reaction contained ~6 nM *Pv*PI4Kβ protein (for ~10% ATP conversion), 10 µM ATP, 0.1 mg/ml L-alpha-phosphatidylinositol (PI; Avanti Polar Lipid, cat. 840042P), 1% (v/v) DMSO and inhibitor in assay buffer. Reactions were incubated for 45 min at 22°C. ADP formation was measured using the ADP-Glo Kinase Kit (Promega). Luminescent signal was measured using the EnSpire Multimode Plate Reader (PerkinElmer). The data was normalised based on the 100% activity controls (1% DMSO only) and the 100% inhibition controls (10 µM MMV390048). Mean IC_{50} values were calculated from $N \geq 2$ independent experiments, each with technical duplicates (log(inhibitor) vs. normalized response - Variable slope). IC_{50} values within 3-fold from independent experiments are considered reproducible.

In vitro P. falciparum PKG Inhibition Assay

Full length *Pf*PKG (*Pf*3D7_1436600) expressed in *Escherichia coli* and purified using methods previously described was used in the assay.[7] [8] Briefly, a 10-point 3-fold serial dilution of each inhibitor was carried out in DMSO (Sigma-Aldrich) and subsequently diluted into assay buffer (25 mM HEPES pH 7.4, 0.1 mg/mL BSA, 0.01 % (v/v) Triton-X 100, 20 mM $MgCl_2$, 2 mM DTT, 10 µM cGMP). The final kinase reactions contained ˜0.6 nM *Pf*PKG protein, 10 µM ATP, 20 µM GRTGRRNSI-NH2 (Sigma-Aldrich, Cat no. SCP0212), 1 % (v/v) DMSO and inhibitor in assay buffer. Reactions were incubated for 45 minutes at 22°C. ADP formation was measured using the ADP-Glo Kinase Kit (Promega). Luminescent signal was measured using the EnSpire Multimode Plate Reader (PerkinElmer). The data was normalised based on the 100% activity controls (1% DMSO only) and the 100% inhibition controls (10 µM ML10 (N-[5-[3-[2-(cyclopropylmethylamino)pyrimidin-4-yl]-7-[(dimethylamino)methyl]-6-methylimidazo[1,2-a]pyridin-2-yl]-2-fluorophenyl]methanesulfonamide), LifeArc). Mean IC_{50} values were calculated from $N \geq 2$ independent experiments, each with technical duplicates (log(inhibitor) vs. normalized response - Variable slope). IC_{50} values within 3-fold from independent experiments are considered reproducible.

Cytotoxicity in the Chinese Hamster Ovarian Cell Line

In vitro cytotoxicity was conducted in CHO cells using the 3-(4,5-dimethylthiazol-2-yl)-2,5-diphenyltetrazolium bromide (MTT) assay.[9] Test compounds were dissolved in 100% DMSO to

yield a 20 mM stock solution while the reference standard, emetine, was dissolved in distilled water to a 2 mg/mL solution. From an initial test compound and control concentration of 100 µg/mL, 10-fold serial dilutions in assay medium were performed to achieve six concentrations with 0.001 µg/mL being the lowest concentration. The highest concentration of DMSO exposed to cells had no effects on cell viability. Plates were incubated for 48 h with 100 µL of drug and 100 µL of cell suspension in each well after which they were developed by the addition of 25 µL of sterile MTT (Thermo Fisher Scientific) to each well and incubated in the dark for 4 hours. Thereafter, the plates were centrifuged, the medium aspirated off and DMSO (100 µL) added to dissolve crystals and absorbance readings taken at 540 nm. Non-linear dose-response curve fitting analysis using GraphPad Prism v.4.0 software (La Jolla, USA) was applied to generate IC_{50} values. The assay was conducted in triplicate and two independent assays (n = 2) were carried out for each compound.

In vitro Microsomal Metabolic Stability

Metabolic stability was conducted using the one-time point assay as described in literature.[10,11] Experiments were performed in triplicate in a 96-well microtiter plate using test compounds at a concentration of 0.1 µM, incubated individually at 37°C in a solution containing 0.35 mg/mL mouse, rat and pooled human liver microsomes (XenoTech, Lenexa, KS).

To initiate metabolic reactions, NADPH (1 mM) in phosphate buffer (100 mM, pH 7.4) was added to the wells and the plates were incubated for 30 min. After this period, 300 µL of ice-cold acetonitrile (ACN) containing an internal standard (carbamazepine, 0.0236 µg/mL) was added to each well to quench the reactions. The supernatant was filtered after which LCMS/MS (Agilent Rapid Resolution HPLC, AB SCIEX 4000 QTRAP MS) analysis was performed to determine the concentration of the test compound. Differences in the amounts of compounds before and after incubation were determined by LC-MS/MS and results recorded as percent compound remaining after 30 min incubation. The relative loss of parent compound over time was monitored and plots of concentration versus time were prepared for each compound to determine the first order rate constant for compound depletion. This was used to calculate the degradation half-life ($t_{1/2}$) and predict the *in vivo* hepatic extraction ratio (E_H). Metabolite identification was not performed during the microsomal metabolic stability assay.

In vitro hERG Inhibition Assay

The hERG inhibition assay was performed on QPatch 16X or QPatchHTX automated patch-clamp system.[12] Initially, test compounds were dissolved in DMSO (Sigma-Aldrich) to a 10 mM stock solution and thereafter serially diluted to the screening concentrations of 30, 10, 3, and 0 µM in freshly prepared extracellular solution to ensure a final 1 × components of NaCl (137 mM), KCl (4 mM), $CaCl_2$ (1.8 mM), $MgCl_2$ (1 mM), D-glucose (10 mM) in HEPES buffer (10 mM) titrated to a pH of 7.4 in NaOH. The intracellular solution used in the system comprised of KCl (130 mM), $CaCl_2$ (2 mM), $MgCl_2$ (4 mM), Na_2-ATP (4 mM), EGTA (5 mM), in HEPES buffer (10 mM) stabilized to a pH of 7.2 in KOH. The potassium ion hERG channel was stably expressed in a CHO cell line, grown in sterile culture flasks in a mixture of HAM/F-12 (1 × liquid with L-Glutamine) supplemented with 10% foetal bovine serum and 1.0% Penicillin/Streptomycin solution, and harvested in standard laboratory conditions.

Cells were then transferred as suspension in serum-free medium to the QPatch system and kept in the cell storage tank/stirrer during the experiments. All solutions applied to cells including the intracellular solution were maintained at room temperature (19 to 30°C). Voltage ramps were then employed, from a holding potential of -80 mV, depolarization of the cell membrane to +20 mV for 2 s and upon subsequent repolarisation to −40 mV for 3 s, the outward hERG tail currents were measured. A pre-programmed waiting time of 5 s was employed, before the next pulse. This application protocol was undertaken in triplicate for the test solutions, vehicle and control standard (E-4031). The normalized currents were then plotted using SigmaPlot 11.0 software, fitted to the Hill equation, upon which IC_{50} and IC_{20} values were deduced.

Solubility using HPLC-based DMSO "dry-down" method

A modified kinetic solubility determination method was employed.[13] Test compounds were dissolved in DMSO (Sigma-Aldrich) to yield 10 mM stock solutions. HPLC analysis was used with UV detection to construct calibration curves for each compound using low (11 µM), medium (100 µM), and high (220 µM) concentration standards. High and medium standards were prepared by placing 4.4 µL and 2 µL of the 10 mM stock solution into wells A and B of a 96-well microtiter plate and diluted by adding 195.6 µL and 198 µL DMSO, respectively. The low standard was prepared in well C by diluting 10 µL, obtained from well A, with 190 µL DMSO to obtain a 20-fold dilution of the 220 µM solution.

Thereafter, each test sample (4 µL of 10 mM stock) was placed in triplicate in wells D, E, and F and DMSO was removed by freeze-drying under Genevac®. This step reduces DMSO-associated solubility enhancement. PBS (200 µL, pH 7.4) was added to the wells now containing dry, solid material from test samples. The plates were covered and placed on a shaker at 37°C for 24 h. After this period, the plates were put under centrifugation (Digtor 21R®) at 3500 rpm for 15 min at a temperature of 23°C. The supernatants were then carefully transferred to an analytical 96-well microtiter plate for HPLC analysis fitted with UV detection. The concentrations of dissolved samples were determined by comparing the average peak areas of the samples (wells D, E, and F) against the previously constructed standard curve using samples in wells A, B, and C.

In vivo Efficacy Studies in *P. berghei*-infected mice

In vivo efficacy was assessed as previously described[14] with the modification that mice (n = 3) were infected with a GFP-transfected *P. berghei* ANKA strain (donated by A. P. Waters and C. J. Janse, Leiden University, The Netherlands), and parasitemia was determined using standard flow cytometry techniques. The detection limit was 1 parasite in 1000 erythrocytes (that is, 0.1%). The activity was calculated as the difference between the mean percent parasitemia for the control and treated groups expressed as a percentage relative to the control group. Compounds were dissolved or suspended in a vehicle consisting of 70% Tween-80 and 30% ethanol, followed by a 10-fold dilution in H2O and oral administration as four consecutive daily doses (4, 24, 48, and 72 h after infection). Blood samples for the quadruple-dose regimens were collected on day 4 (96 h after infection).

The survival time in days was also recorded up to 30 days after infection. A compound was considered curative if the animal survived to day 30 after infection with no detectable parasites by slide reading.

In vivo studies conducted at the Swiss TPH, Basel were approved by the veterinary authorities of the Canton Basel-Stadt (Permit No. 1731) based on Swiss Cantonal (Verordnung Veterinäramt Basel-Stadt) and National Regulations (The Swiss Animal Protection Law, Tierschutzgesetz).

In Vivo Pharmacokinetic Studies

Ethical Statement: All studies and procedures were conducted with prior approval of the animal ethics committee of the University of Cape Town (approval numbers 022_004) in accordance with

the South African National Standard (SANS 10386:008) for the Care and Use of Animals for Scientific Purposes[15] and guidelines from the Department of Health[16].

Animal studies: Female BALB/c mice were bred at the University of Cape Town Research Animal Facility, Cape Town, South Africa. Compound **19** was administered intravenously (0.5 mg/kg) to female BALB/c mice (n=3) as a bolus of 5% (v/v) dimethylacetamide (DMA), 30% (v/v) propylene glycol, 15% polyethyleneglycol (PEG) 400 and 50% water. The oral dose (1 mg/kg) was administered to mice (n=3) as an aqueous suspension containing 0.5% (w/v) hydroxypropylmethylcellulose (HPMC). Mice were not fasted overnight and were allowed to eat ad libitum. Animals were permitted access ad libitum to water.

Sample analysis: Blood samples were collected from the tail vein of mice into heparinized microcentrifugation tubes at pre-determined timepoints stored frozen (-80°C) until analysis.

Bioanalytical method: The concentration of **19** was determined by LC-MS/MS using an AB Sciex API5500 triple quadrupole instrument. Chromatographic separation was conducted using an Agilent 1200 series HPLC. Blood samples and calibration standards (prepared in drug-free whole blood of the relevant species) were prepared using protein precipitation with cold acetonitrile, followed by centrifugation and analysis of the supernatant fraction. The analytical limit of quantitation (LOQ) was 2 ng/mL. The accuracy, precision, and recovery for each study were within acceptable limits. Mouse pharmacokinetic (PK) parameters were calculated by non-compartmental analysis using PK Solutions 2.0 (Summit Research Services, Montrose, CO, USA) with a method based on curve stripping.

5. Supplementary Tables for Biological, Enzymatic and Solubility Data

Chemical structures, and biological and solubility data of amides and sulfonamides

Table S1. *In vitro* Asexual Blood Stage Activity Against *Pf* NF54, *in vitro* *Pv*PI4Kβ and *Pf*PKG inhibition, and solubility results

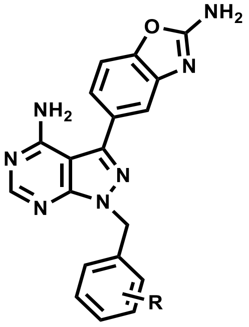

Code	R	*Pf*NF54 IC_{50} (SD), μM	*Pf*K1 IC_{50} (SD), μM	*Pv*PI4Kβ IC_{50} (SD), μM[a]	*Pf*PKG IC_{50} (SD), μM[b]	Solubility, μM[c]
**46a**	4-$CONH_2$	1.5 (0.5)	-	0.012 (0.003)	0.78 (0.63)	20
**46b**	3-$CONH_2$	0.66 (0.15)	0.54 (0.04)	0.004 (0.001)	0.13 (0.09)	15
**46c**	2-$CONH_2$	1.4 (0.2)	1.2 (0.01)	0.004 (0.002)	0.30 (0.14)	180
**46d**	4-SO_2NH_2	>6	-	0.012 (0.002)	0.35 (-)	-
**46e**	4-SO_2NHMe	0.65 (0.15)	3.5 (0.3)	0.013 (0.004)	0.25 (-)	10
**46f**	4-$SO_2N(Me)_2$	4.9 (0.0)	0.69 (0.03)	0.011 (0.002)	0.80 (0.37)	<5
**46g**	3-$SO_2N(Me)_2$	0.40 (0.01)	-	0.004 (0.001)	0.18 (0.01)	<5
**46h**	3-SO_2NHMe	3.5 (0.09)	-	0.002 (0.001)	0.22 (0.05)	5

[a]*In vitro* *Pv*PI4Kβ inhibition at 10 μM ATP. [b]*In vitro* *Pf*PKG inhibition at 10 μM ATP. Results are from N ≥ 2 independent experiments. [c]Determined at pH 6.5, using an HPLC-based miniaturized shake flask method. "-" = data not generated.

Table S2. *In vitro* human mTOR and PI4Kβ inhibition results for carboxamides and sulfonamides

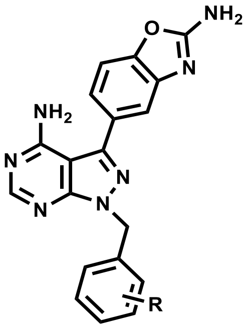

Code	R	mTOR IC$_{50}$ (µM)	HuPI4Kβ IC$_{50}$ (µM)
**46a**	4-CONH$_2$	0.013	>10
**46b**	3-CONH$_2$	0.002	>10
**46c**	2-CONH$_2$	0.009	>10
**46d**	4-SO$_2$NH$_2$	0.277	>10
**46e**	4-SO$_2$NHMe	0.051	>10
**46f**	4-SO$_2$N(Me)$_2$	0.038	>10
**46g**	3-SO$_2$N(Me)$_2$	0.004	>10
**46h**	3-SO$_2$NHMe	0.008	>10

Cytotoxicity data in the Chinese Hamster Ovarian (CHO) and HepG2 Cell lines

Table S3. *In vitro* cytotoxicity activity of other sapanisertib compounds

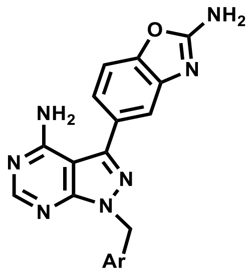

Ar	R	Code	[a]CHO IC$_{50}$, µM	[b]SI	[c]% Inh.HepG2 @ 2 µM (SD)
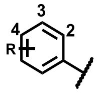	4-Cl	7	1.19	40	10
	4-F	8	9.04	108	15
	4-CF$_3$	9	> 50	> 226	6
	4-NH$_2$	10	-	-	9
	3-F	11	> 50	> 168	21
	3-CF$_3$	12	> 50	> 262	8
	3-Cl	13	> 50	> 388	7.5
	3-NH$_2$	14	-	-	39
	3-N(Me)$_2$	15	1.57	4	48
	3,4-Cl	20	> 50	>1250	7
	2-F, 4-Cl	22	1.17	12	147
	3-F, 4-Cl	19	1.98	99	9
	2-F, 4-F	23	0.61	3	8
	3-Cl, 4-F	25	24.0	267	8
	3-F, 4-F	26	0.62	15	11
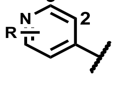	3-Cl	32	40.8	408	3
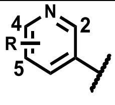	4-CF$_3$	27	>50	> 133	-
	5-Me	34	39.85	210	41
	5-Cl	35	0.60	4	69

	5-F	36	0.69	2	52
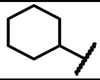	H<br>4-*di* F	37<br>38	5.67<br>0.22	15<br>1.2	20<br>72

[c]Percentage (%) inhibition determined for one biological replicate (N = 1) with technical duplicates

Gametocytocidal activity against immature and late-stage gametocytes

Table S4. *In vitro* gametocytocidal activity of other compounds against *Pf* immature (iGc)- and late-stage gametocytes (LGc)

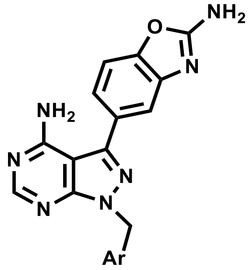

Ar	R	Code	[a]% Inh. iGc @ 1 and 5 μM		[a]% Inh. LGc @ 1 and 5 μM	
			**1**	**5**	**1**	**5**
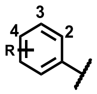	4-Cl<br>4-F<br>4-CF_3<br>4-NH_2<br>3-F<br>3-CF_3<br>3-Cl<br>3-NH_2<br>3-N$(Me)_2$<br>3,4-Cl<br>2-F, 4-Cl<br>3-F, 4-Cl<br>2-F, 4-F<br>3-Cl, 4-F<br>3-F, 4-F	7<br>8<br>9<br>10<br>11<br>12<br>13<br>14<br>15<br>20<br>22<br>19<br>23<br>25<br>26	8.1<br>0.0<br>0.0<br>0.0<br>0.0<br>5.1<br>0.0<br>0.0<br>0.0<br>0.0<br>7.1<br>0.4<br>0.0<br>2.3<br>5.5	39.5<br>0.0<br>0.0<br>5.6<br>23.8<br>0.0<br>14.6<br>0.0<br>0.0<br>4.7<br>18.6<br>50.5<br>1.6<br>49.2<br>61.9	68.3<br>1.2<br>2.7<br>0.0<br>9.2<br>0.0<br>5.4<br>12.5<br>0.0<br>34.7<br>45.6<br>71.9<br>18.4<br>31.5<br>55.0	84.8<br>76.9<br>57.4<br>0.0<br>57.4<br>68.7<br>68.6<br>39.0<br>5.8<br>80.7<br>88.2<br>88.5<br>78.7<br>68.0<br>76.0
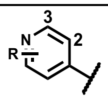	3-Cl	32	0.0	0.0	23.9	73.1
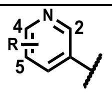	5-Me<br>5-Cl<br>5-F	34<br>35<br>36	0.0<br>0.0<br>0.0	0.0<br>0.0<br>0.0	22.5<br>51.0<br>17.8	70.9<br>79.3<br>71.6

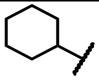	H	37	0.0	56.6	8.4	61.3
	4-*di* F	38	0.0	0.0	29.4	73.0

[a]Percentage (%) inhibition of *P. falciparum* immature (iGc) and late-stage gametocytes (LGc), N = 1, with technical triplicates.

6. Associated References

(1) Fienberg, S.; Eyermann, C. J.; Arendse, L. B.; Basarab, G. S.; McPhail, J. A.; Burke, J. E.; Chibale, K. Structural Basis for Inhibitor Potency and Selectivity of Plasmodium Falciparum Phosphatidylinositol 4-Kinase Inhibitors. *ACS Infect. Dis.* **2020**, *6* (11), 3048–3063. https://doi.org/10.1021/acsinfecdis.0c00566.

(2) Arendse, L. B.; Murithi, J. M.; Qahash, T.; Pasaje, C. F. A.; Godoy, L. C.; Dey, S.; Gibhard, L.; Ghidelli-Disse, S.; Drewes, G.; Bantscheff, M.; Lafuente-Monasterio, M. J.; Fienberg, S.; Wambua, L.; Gachuhi, S.; Coertzen, D.; van der Watt, M.; Reader, J.; Aswat, A. S.; Erlank, E.; Venter, N.; Mittal, N.; Luth, M. R.; Ottilie, S.; Winzeler, E. A.; Koekemoer, L. L.; Birkholtz, L. M.; Niles, J. C.; Llinás, M.; Fidock, D. A.; Chibale, K. The Anticancer Human MTOR Inhibitor Sapanisertib Potently Inhibits Multiple Plasmodium Kinases and Life Cycle Stages. *Sci. Transl. Med.* **2022**, *14* (667), eabo7219. https://doi.org/10.1126/scitranslmed.abo7219.

(3) Snyder, C.; Chollet, J.; Santo-Tomas, J.; Scheurer, C.; Wittlin, S. In Vitro and in Vivo Interaction of Synthetic Peroxide RBx11160 (OZ277) with Piperaquine in Plasmodium Models. *Exp. Parasitol.* **2007**, *115* (3), 296–300. https://doi.org/10.1016/j.exppara.2006.09.016.

(4) Reader, J.; Botha, M.; Theron, A.; Lauterbach, S. B.; Rossouw, C.; Engelbrecht, D.; Wepener, M.; Smit, A.; Leroy, D.; Mancama, D.; Coetzer, T. L.; Birkholtz, L. M. Nowhere to Hide: Interrogating Different Metabolic Parameters of Plasmodium Falciparum Gametocytes in a Transmission Blocking Drug Discovery Pipeline towards Malaria Elimination. *Malar. J.* **2015**, *14* (1), 1–17. https://doi.org/10.1186/s12936-015-0718-z.

(5) Reader, J.; van der Watt, M. E.; Birkholtz, L. M. Streamlined and Robust Stage-Specific Profiling of Gametocytocidal Compounds Against Plasmodium Falciparum. *Front. Cell. Infect. Microbiol.* **2022**, *12* (June), 1–11. https://doi.org/10.3389/fcimb.2022.926460.

(6) McNamara, C. W.; Lee, M. C. S.; Lim, C. S.; Lim, S. H.; Roland, J.; Nagle, A.; Simon, O.; Yeung, B. K. S.; Chatterjee, A. K.; McCormack, S. L.; Manary, M. J.; Zeeman, A. M.; Dechering, K. J.; Kumar, T. R. S.; Henrich, P. P.; Gagaring, K.; Ibanez, M.; Kato, N.; Kuhen, K. L.; Fischli, C.; Rottmann, M.; Plouffe, D. M.; Bursulaya, B.; Meister, S.; Rameh, L.; Trappe, J.; Haasen, D.; Timmerman, M.; Sauerwein, R. W.; Suwanarusk, R.; Russell, B.; Renia, L.; Nosten, F.; Tully, D. C.; Kocken, C. H. M.; Glynne, R. J.; Bodenreider, C.; Fidock, D. A.; Diagana, T. T.; Winzeler, E. A. Targeting Plasmodium PI(4)K to Eliminate Malaria. *Nature* **2013**, *504* (7479), 248–253. https://doi.org/10.1038/nature12782.

(7) Cheuka, P.; Centani, L.; Arendse, L.; Fienberg, S.; Wambua, L.; Renga, S.; Dziwornu, G.; Kumar, M, Lawrence, N.; Taylor, D.; Wittlin, S.; Coertzen, D.; Reader, J.; Van Der Watt, M.; Birkholtz, L.; Chibale, K. New Amidated 3,6-Diphenylated Imidazopyridazines with Potent Antiplasmodium Activity Are Dual Inhibitors of Plasmodium Phosphatidylinositol-4-Kinase and CGMP-Dependent Protein Kinase. *ACS Infect. Dis.* **2021**, *7* (1), 34–46. https://doi.org/10.1021/acsinfecdis.0c00481.

(8) Vanaerschot, M.; Murithi, J. M.; Pasaje, C. F. A.; Lee, M. C. S.; Niles, J. C.; Fidock, D. A.; Vanaerschot, M.; Murithi, J. M.; Pasaje, C. F. A.; Ghidelli-disse, S.; Dwomoh, L.; Bird, M.; Spottiswoode, N.; Mittal, N.; Arendse, L. B.; Owen, E. S.; Wicht, K. J.; Siciliano, G.; Tobin, A. B.; Doerig, C.; Winzeler, E. A.; Lee, M. C. S.; Niles, J. C. Inhibition of Resistance-Refractory P . Falciparum Kinase PKG Delivers Prophylactic, Blood Stage, and Transmission-Blocking Antiplasmodial Activity. **2020**, 1–11. https://doi.org/10.1016/j.chembiol.2020.04.001.

(9) Liu, Y.; Peterson, D. a; Kimura, H.; Schubert, D. Mechanism of Cellular 3-(4,5-Dimethylthiazol-2-Yl)-2,5- Diphenyltetrazolium Bromide (MTT) Reduction. *J. Neurochem.* **1997**, *69* (2), 581–593.

(10) Kandepedu, N.; Gonzàlez Cabrera, D.; Eedubilli, S.; Taylor, D.; Brunschwig, C.; Gibhard, L.; Njoroge, M.; Lawrence, N.; Paquet, T.; Eyermann, C. J.; Spangenberg, T.; Basarab, G. S.; Street, L. J.; Chibale, K. Identification, Characterization, and Optimization of 2,8-Disubstituted-1,5-Naphthyridines as Novel Plasmodium Falciparum Phosphatidylinositol-4-Kinase Inhibitors with in Vivo Efficacy in a Humanized Mouse Model of Malaria. *J. Med. Chem.* **2018**, *61* (13), 5692–5703. https://doi.org/10.1021/acs.jmedchem.8b00648.

(11) Gibhard, L.; Pravin, K.; Abay, E.; Wilhelm, A.; Swart, K.; Lawrence, N.; Khoury, R.; Van Der Westhuizen, J.; Smith, P.; Wiesner, L. In Vitro and in Vivo Pharmacokinetics of Aminoalkylated Diarylpropanes NP085 and NP102. *Antimicrob. Agents Chemother.* **2016**, *60* (5), 3065–3069. https://doi.org/10.1128/AAC.02104-15.

(12) Houtmann, S.; Schombert, B.; Sanson, C.; Partiseti, M.; Bohme, G. A. Automated Patch-Clamp Methods for the HERG Cardiac Potassium Channel. In *Drug Safety Evaluation: Methods and Protocols, Methods in Molecular Biology,*; 2017; Vol. 1641, pp 187–199. https://doi.org/10.1007/978-1-4939-7172-5.

(13) Glomme, A.; März, J.; Dressman, J. B. Comparison of a Miniaturized Shake-Flask Solubility Method with Automated Potentiometric Acid/Base Titrations and Calculated Solubilities. *J. Pharm. Sci.* **2005**, *94* (1), 1–16. https://doi.org/10.1002/jps.20212.

(14) González Cabrera, D.; Douelle, F.; Younis, Y.; Feng, T. S.; Le Manach, C.; Nchinda, A. T.; Street, L. J.; Scheurer, C.; Kamber, J.; White, K. L.; Montagnat, O. D.; Ryan, E.; Katneni, K.; Zabiulla, K. M.; Joseph, J. T.; Bashyam, S.; Waterson, D.; Witty, M. J.; Charman, S. A.; Wittlin, S.; Chibale, K. Structure-Activity Relationship Studies of Orally Active Antimalarial 3,5-Substituted 2-Aminopyridines. *J. Med. Chem.* **2012**, *55* (24), 11022–11030. https://doi.org/10.1021/jm301476b.

(15) SABS Standards Division. *The Care and Use of Animals for Scientific Purposes*; **2008**.

(16) RSA Department of Health. Ethics in Health Research Principles, Processes and Structures, A Long and Healthy Life for All South Africans. **2015**, 1–65.
